# Message Design Choices Don't Make Much Difference to Persuasiveness and Can't Be Counted On—Not Even When Moderating Conditions Are Specified

**DOI:** 10.3389/fpsyg.2021.664160

**Published:** 2021-06-29

**Authors:** Daniel J. O'Keefe, Hans Hoeken

**Affiliations:** ^1^Department of Communication Studies, Northwestern University, Evanston, IL, United States; ^2^Department of Languages, Literature, and Communication, Utrecht University, Utrecht, Netherlands

**Keywords:** behavior change, message design, message variables, message variations, meta-analysis, persuasion, persuasive communication, prediction intervals

## Abstract

Persuaders face many message design choices: narrative or non-narrative format, gain-framed or loss-framed appeals, one-sided or two-sided messages, and so on. But a review of 1,149 studies of 30 such message variations reveals that, although there are statistically significant differences in persuasiveness between message forms, it doesn't make much difference to persuasiveness which option is chosen (as evidenced by small mean effect sizes, that is, small differences in persuasiveness: median mean *r*s of about 0.10); moreover, choosing the on-average-more-effective option does not consistently confer a persuasive advantage (as evidenced by 95% prediction intervals that include both positive and negative values). Strikingly, these results obtain even when multiple moderating conditions are specified. Implications for persuasive message research and practice are discussed.

Designing effective persuasive messages is an important communicative task. Encouraging people to exercise regularly, vote for a candidate, buy a product, reduce home energy consumption, donate to a charity, be screened for a disease, alter their diet, give blood, wear seat belts—all these purposes can potentially be advanced through persuasive messages.

Considerable experimental research has explored the effects of message design choices that persuaders might face, such as using gain-framed or loss-framed messages, narrative or non-narrative formats, strong or weak threat appeals, and so on. In an individual experiment, the size and direction of the difference in persuasiveness between the two message forms being compared can be represented by an effect size index such as *d* (standardized mean difference) or *r* (correlation). A large effect size indicates a large difference in the persuasiveness of the two message forms being compared; a small effect size indicates little difference in persuasiveness. For many message design choices (message variables), meta-analytic reviews have provided a good evidentiary basis for conclusions about how such choices affect relative message persuasiveness.

Two properties of these effects are naturally of interest to message designers. One is the size of the effect, that is, the mean effect size: How large a persuasive advantage is conferred on average by choosing the message form that is generally more effective? The other is the consistency of the effect across studies: How much does the persuasive advantage vary from one application to another? From a message designer's point of view, of course, the ideal would be to know of large, consistent effects—message design choices that would dependably produce large differences in relative persuasiveness.

The broad purpose of this paper is to describe the size and consistency of the effects of persuasive message design choices, using evidence from extant meta-analyses. Previous review discussions of such meta-analyses have focused on the average size of the main (simple, non-contingent) effects of design choices (message variations). The present review aims to deepen such analyses in two ways. First, it considers not only the main effects of design choices but also contingent effects, that is, effects observed under moderating conditions. Second, it considers not only the size but also the consistency of both main and contingent message-variation effects. The next section elaborates these purposes and provides a more careful specification of the approach taken.

## Background

### Previous Reviews

Summaries of the relevant meta-analytic evidence have consistently concluded that the mean effect sizes associated with persuasive message design choices are rather small. For example, over 20 years ago, Dillard (1998) reviewed nine such meta-analyses, reporting that the mean effect size (expressed as *r*) was only 0.18. Weber and Popova (2012) extracted 206 effect sizes from “persuasion effects” meta-analyses; the median *r* was 0.11 (similarly, see Rains et al., 2018).

Such previous summaries are useful but are limited in two ways. First, these summaries have focused on the main (simple, non-contingent) effects of persuasive message design choices. The finding that such effects are not large is perhaps unsurprising. A common expectation seems to be that the effects of message design choices are inevitably contingent on various moderating factors. Crudely put, there might be “interaction effects all the way down” (Vivalt, 2015, p. 468). As Rains et al. (2018, p. 121) pointed out, their focus on main effects might make for a misleading picture because larger effects could occur under specific moderating conditions. So even if on average a given design choice enhances persuasion only a little, more substantial increases might be observed under the right set of moderating conditions.

Second, previous summaries are limited by having focused on the *size* of the effects, with little attention given to effect *variability* (heterogeneity). Attending to heterogeneity can be important for various purposes, such as serving as a goad to theoretical progress. For example, Linden and Hönekopp (2021) have argued that in psychological research, unexplained heterogeneity is a sign that the phenomenon is poorly understood, and hence reduction of such heterogeneity is a marker of research progress (see also Kenny and Judd, 2019). Where these purposes are central, an interest in moderator variables will lead to questions about whether moderators can explain variability.

In the present project, however, our primary interest in the heterogeneity of message-variation effect sizes has a different purpose, namely, to consider the implications of such variability for message design practices. The consistency of effect sizes associated with a given message variation is especially relevant to persuasive message design recommendations.

Imagine, for example, two message variations with identical mean effect sizes—but for one message variation the observed effect sizes all cluster closely around that mean (little variability from study to study in the size of the persuasive advantage conferred) whereas for the other message variation the observed effect sizes are quite variable. One would have rather more confidence in the expected effects of the first design choice than in those of the second. As another illustration: Even if a given message design variation produces only a small mean effect size (only a small average persuasive advantage from choosing the more effective message form), that small advantage may be especially valuable if it can be obtained consistently.

And, plainly, considerations of effect-size variability are relevant to both main effects and contingent effects. It is important to understand how consistently a given message design choice yields a persuasive advantage, whether in general or when moderating conditions are specified.

Briefly, then: Where previous summaries of message-variation meta-analyses have focused on the size of main (non-contingent) effects, the present report examines both the size and the variability of both main effects and contingent effects, that is, effects observed under specified moderating conditions. Comparisons between main effects and contingent effects are of special interest. It seems natural to expect that as moderating conditions are taken into account, the size of the persuasive advantage enjoyed by the more effective message form can increase (i.e., under appropriate moderating conditions, effect sizes will increase) and the persuasive advantage can be obtained more consistently (i.e., under appropriate moderating conditions, effect sizes will become more consistent), compared to what is observed when examining only main effects.

Describing the *size* of message-variation effects is familiar and straightforward: one examines the mean effect size, that is, the average difference in persuasiveness between the two message forms. However, effect-size *variability* has not yet been the focus of much explicit attention, so the next section provides some background.

### Describing Effect-Size Variability

For describing the variability of effect sizes, one might initially think of various meta-analytic heterogeneity indices such as *I*^2^, *Q, H*, Birge's R, and the like (for some discussions of such indices, see Birge, 1932; Higgins and Thompson, 2002; Higgins et al., 2003; Huedo-Medina et al., 2006; Card, 2012, p. 184–191). But these heterogeneity indices do not capture the desired property. Broadly speaking, these indices express observed heterogeneity as a proportion of, or a ratio involving, the heterogeneity that might be expected from human sampling variation alone. For example, Birge's R is the ratio of observed to expected variation; *I*^2^ describes the percentage of observed variation that is not attributable to chance (Higgins et al., 2003, p. 558).

The implication is that such indices do not provide the desired information because the indices do not describe how effect sizes vary in absolute terms. This point is easily misunderstood, which can lead to erroneous interpretations of values of indices such as *I*^2^ (e.g., thinking that if *I*^2^ is large then the effect sizes vary considerably from study to study). In fact, as Borenstein et al. (2017) have explained, because *I*^2^ is a proportion rather than an absolute value, it cannot indicate how much effect sizes vary (see also Rücker et al., 2008).

However, the desired property can be described using prediction intervals. Expressed in terms of the context of present interest, a prediction interval specifies the plausible range of effect sizes to be observed in the next application (e.g., the next study). Thus, “a prediction interval provides a description of the plausible range of effects if the treatment is applied in a new study or a new population similar to those included in the meta-analysis” (Partlett and Riley, 2017, p. 302). “A 95% prediction interval estimates where the true effects are to be expected for 95% of similar (exchangeable) studies that might be conducted in the future” (IntHout et al., 2016, p. 2).

Prediction intervals (PIs) are not to be confused with confidence intervals (CIs), as these address different questions (see Borenstein, 2019, p. 94–96). The 95% CI around a meta-analytic mean effect size gives the range of plausible population (mean) values; the 95% PI gives the range of plausible future individual effect sizes. The relationship of PIs and CIs is sometimes misunderstood because of a misapprehension about CIs—specifically, a belief that the CI describes the dispersion of effects in individual studies. It does not. The CI describes the range within which the *mean* effect (the population effect) is likely to be found. But answering the question “where is the mean effect likely to be?” is different from answering the question “where is the effect size in an individual study likely to be?”

Thus, PIs are not a replacement for, or an alternative to, or a competitor with, CIs. Rather, PIs provide information that CIs do not and hence are useful adjuncts to CIs. Specifically, the PI gives the range of plausible future *individual* effect sizes, that is, the range of plausible results in individual studies. If one wants to know where an effect size might fall in a future application, the PI is informative but the CI is not.

Without prediction-interval information, one cannot be sure just how confidently to recommend a given message design choice. For example, suppose there is a statistically significant mean effect favoring message form A over message form B (either in general or under some specific set of moderating conditions); that is, the 95% CI around the meta-analytic mean effect excludes zero. Knowing that the *mean* effect favors form A does not necessarily imply that every future *individual* application will favor form A; it's a separate question how consistently form A delivers some advantage over form B. That question cannot be answered by the CI; to see the plausible range of individual effect sizes, the PI is wanted.

So if the 95% PI for that comparison of form A and form B *also* excludes zero, then choosing form A is pretty much a sure thing; it's very unlikely that message form B would ever turn out to be more effective in the relevant circumstances. On the other hand, if the 95% PI straddles zero and so includes both positive and negative values, then one can't count on form A being the better choice; sometimes form B will turn out to be more effective (despite the statistically significant *mean* effect, that is, even though the 95% CI excludes zero).

In short, prediction intervals provide exactly the sort of representation of effect consistency that is wanted for the present application (For some discussion of prediction intervals, see Higgins et al., 2009; Riley et al., 2011; IntHout et al., 2016; Borenstein, 2019, p. 85–93; Nagashima et al., 2019). To be sure, PIs are imperfect; for example, with fewer than ten cases, PIs will understandably be imprecise (see Meeker et al., 2017, p. 180, Figure 10.1). But for speaking to the question of the consistency of expected future effect sizes, PIs provide information that neither familiar heterogeneity indices nor CIs do.

### Summary

Persuasive message designers would hope to learn of message design choices that consistently make for large differences in persuasiveness, either in general (main effects) or contingently (when moderating conditions are specified). Existing meta-analytic evidence is relevant to identifying such choices, but previous reviews of those meta-analyses have focused only on the size of main effects. There has not been systematic examination of the variability of main-effect effect sizes or of the magnitude and variability of effect sizes when moderating conditions are taken into account. The research reported here aims to remedy that lack.

But we are at some pains to emphasize: Our research questions concern the relative persuasiveness of alternative message forms, not the absolute persuasiveness of a message or message form. The data of interest in the present project—the effect sizes—do not address questions of how effective persuasive messages are or can be. Some readers have thought that (e.g.,) small effect sizes in the studies under review here indicate that persuasive messages are not very effective. This reflects a misapprehension. In a study comparing the effectiveness of two persuasive message forms (e.g., narrative vs. non-narrative), the effect size of interest describes the *difference* in persuasive effectiveness between the two messages—not the effectiveness of either message individually or the average effectiveness of the two messages (O'Keefe, 2017). If two messages are both highly effective and so don't differ much in effectiveness, the effect size will be small even though each message was in absolute terms quite effective. To address the questions of interest here—questions about the size and consistency of the *differences* in persuasiveness associated with message design choices—the effect sizes analyzed provide exactly the sort of data needed.

## Methods

### Overview

Existing relevant meta-analyses of persuasive message variations were identified. To provide a uniform basis for comparison, each meta-analysis's set of effect sizes (ESs) was analyzed using random-effects procedures to provide a mean ES, a 95% CI, and a 95% PI—both for the main effect of the message variation and for contingent effects (within levels of moderator variables).

### Literature Search and Inclusion Criteria

Meta-analyses of potential interest were identified by searches of the PsycEXTRA, ProQuest Dissertations and Theses, PsycINFO, ERIC, Medline, and Web of Science databases, combining *meta-analysis* with such terms as *persuasion, message*, and *attitude*, through November 2019. Relevant reviews of meta-analyses were also examined to identify potential candidates (e.g., Eisend and Tarrahi, 2016; Hornik et al., 2016, 2017; Rains et al., 2018). Additional candidates were located through examination of textbooks and through personal knowledge of the literature.

The meta-analyses of interest were ones that reviewed experimental studies comparing two versions of a message that varied with respect to some specified message property, where relative persuasiveness was assessed; the ESs of interest thus represented relative effects on belief, attitude, intention, or behavior outcomes, or composite relative effects on more than one of these (as when a meta-analysis reported composite ESs, e.g., combining attitude and intention effects). Broadly put, meta-analyses were excluded if they did not speak to the effects of persuasive message design choices.

Specifically: Meta-analyses were excluded if the review concerned studies that examined the effects of non-message variations (e.g., Kumkale et al., 2010; Boster et al., 2016) or the presence (vs. absence) of some preceding message (e.g., Dillard et al., 1984; De Leeuw et al., 2007; Feeley et al., 2012), compared a message form against a no-message control condition (e.g., Finitsis et al., 2014; Braddock and Dillard, 2016; Chan et al., 2017), examined outcomes other than belief, attitude, intention, or behavior (e.g., O'Keefe and Jensen, 2008; Portnoy et al., 2014; Lee et al., 2017), or were not exclusively experimental studies (e.g., Keller and Lehmann, 2008; Tukachinsky and Tokunaga, 2013).

Similarly, meta-analyses were excluded if the studies reviewed concerned the effects of communication campaigns (e.g., Snyder et al., 2004; Feeley and Moon, 2009; Werb et al., 2011), intervention programs (e.g., Seo and Sa, 2010; Head et al., 2013; Griffiths et al., 2018), or psychological states such as transportation, guilt, or anger (e.g., Van Laer et al., 2014; Xu and Guo, 2018; Walter et al., 2019).

Meta-analyses were excluded if information about the effect sizes and associated sample sizes was unavailable even after correspondence with authors (e.g., Compeau and Grewal, 1998; Reinard, 1998; Floyd et al., 2000; Piñon and Gambara, 2005; Freling et al., 2014). If multiple meta-analyses of a given message variation were available, the one with the largest number of ESs was selected; any meta-analysis based on fewer ESs would, ceteris paribus, provide less accurate estimates of the properties of interest^1^. For similar reasons, if a meta-analysis of a given message variation reported results separately for different persuasion outcomes (e.g., separate results for attitude and for intention), the outcome with the largest number of ESs was selected; different persuasion outcomes have been found to yield equivalent mean effect sizes (O'Keefe, 2013) and hence could be treated as interchangeable. Because “when the analysis includes at least ten studies, the prediction interval is likely to be accurate enough to be useful” (Borenstein, 2019, p. 93), we excluded meta-analyses with fewer than 10 ESs (e.g., Burrell and Koper, 1998; Bigsby and Wang, 2015; Lull and Bushman, 2015, concerning violent content; O'Keefe, 1998, concerning quantification; O'Keefe, 2000, concerning guilt)^2^. Finally, we excluded a meta-analysis if the list of ESs included multiple ESs based on the same message pair or set of participants, as when separate ESs were entered for two different attitude measures from the same participants (e.g., Eisend, 2006).

A list of excluded meta-analyses (with reasons for exclusion) is provided in Appendix 1. But to illustrate the application of these principles, a few specific examples may be useful.

Braddock and Dillard's (2016) meta-analysis of narrative messages was excluded because the studies reviewed did not have an appropriate comparison condition. This meta-analysis included only studies that compared a narrative message against a control condition lacking the narrative message or any message like it (e.g., no-message controls and irrelevant-message controls). Such studies speak to the question of whether using a narrative message is more persuasive than staying silent on the subject. The present project is focused on a different question: Given that some message is to be used and hence some message design choices faced, which design options are relatively more persuasive? Thus, for example, Shen's et al. (2015) meta-analysis, which reviewed studies comparing the persuasiveness of narrative and non-narrative messages, was included in the present analysis; such studies speak to the question of whether a message designer should favor a narrative or a non-narrative format.

Tannenbaum's et al. (2015) meta-analysis was excluded because it compared fear appeals against a combination of a number of different control conditions, including no-message control conditions (see Tannenbaum's et al., 2015, p. 1183). Because effect sizes were collapsed across a variety of different control conditions (high-fear message vs. no-message control, high-fear message vs. low-fear message, etc.), the reported mean effect sizes could not be interpreted straightforwardly as bearing on the present research questions. However, White and Albarracín (2018) reported meta-analytic results for a subset of cases concerning specifically the high-fear-vs.-low-fear message contrast; that meta-analysis was included in the present analysis.

Noar's et al. (2016) meta-analysis of pictorial vs. text-only cigarette pack warnings was excluded because no reported relevant outcome had a sufficiently large number of cases. This meta-analysis reported results for 17 different outcome variables (see p. 347, Table 3), but only one specific outcome had more than 10 effect sizes (the minimum required for inclusion in the present project): “negative affective reactions” such as disgust or fear, which are not outcomes of interest in the present analysis. Relevant outcomes (such as negative smoking attitudes and intentions to quit smoking) had fewer than 10 effect sizes.

### Included Meta-Analyses

These inclusion criteria yielded a total of 30 meta-analyses concerning the effects of diverse persuasive message design choices. Details about each message variation and its corresponding meta-analytic data are provided in Appendix 2 of the Supplementary Materials.

Briefly, the message variations were: appeal framing (gain vs. loss; ESs from O'Keefe and Jensen, 2006); argument explicitness (explicit vs. implicit; O'Keefe, 1998); argument strength (strong vs. weak; Carpenter, 2015); “but you are free” (included vs. omitted; Carpenter, 2013); conclusion (included vs. omitted; O'Keefe, 2002); cultural tailoring (deep-tailored vs. not-tailored; Hornikx and O'Keefe, 2009); depicted response efficacy (high vs. low; Witte and Allen, 2000); depicted self-efficacy (high vs. low; Witte and Allen, 2000); depicted threat severity (high vs. low; De Hoog et al., 2007); depicted threat vulnerability (high vs. low; De Hoog et al., 2007); disrupt-then-reframe (vs. reframe-only; Carpenter and Boster, 2009); evidence amount (high vs. low; Stiff, 1985, 1986); evidence type (narrative vs. statistical; Allen and Preiss, 1997); humor (humorous vs. non-humorous; Walter et al., 2018); information-source identification (included vs. omitted; O'Keefe, 1998); language intensity (high vs. low; Hamilton and Hunter, 1998); legitimizing paltry contributions (included vs. omitted; Bolkan and Rains, 2017); metaphorical (vs. non-metaphorical; Brugman et al., 2019); narrative (vs. non-narrative; Shen et al., 2015); political advertising tone (negative vs. positive; Lau et al., 2007); recommendation specificity (specific vs. general; O'Keefe, 2002); rhetorical questions (vs. statements; Gayle et al., 1998); sexual content (vs. non-sexual; Lull and Bushman, 2015); sidedness (one-sided vs. two-sided; O'Keefe, 1999); speaking rate (faster vs. slower; Preiss et al., 2014); “that's not all” (included vs. omitted; Lee et al., 2019); threat appeal strength (strong vs. weak; White and Albarracín, 2018); victim description (identifiable vs. non-identifiable; Lee and Feeley, 2016); visual material (text-plus-visual vs. text-only; Seo, 2020, and Seo and Kim, 2018); and vividness (vivid vs. pallid; Blondé and Girandola, 2016).

### Analysis

Each meta-analysis identified by these inclusion criteria provided a set of effect sizes and associated sample sizes for a given message variation (design choice). The ESs were accepted as given in each meta-analytic dataset, without being adjusted, deleted, recomputed, or otherwise altered, save for converting all ESs to correlations (*r*s) for analysis. The ESs were analyzed in three ways.

#### Main-Effects Analysis

First, each meta-analysis's set of effect sizes was analyzed using random-effects methods to provide an estimate of the mean effect and the associated 95% CI (Borenstein et al., 2005). The 95% PI was obtained using procedures described by Borenstein et al. (2017; see also Borenstein, 2019, p. 85–93; Borenstein et al., 2009, p. 127–133). When PI widths were analyzed, the width was computed as the simple difference between the upper and lower bounds of the PI.

#### One-Moderator Analysis

Second, for each set of effect sizes, results for any reported categorical moderator variables were also examined. Because of our interest in guidance for message design, we examined moderators concerning attributes of messages (e.g., content variations) and audiences (e.g., sex) and excluded those concerning study characteristics (e.g., publication outlet). If a level of such a moderator variable had at least 10 ESs, the mean effect size, 95% CI, and 95% PI were computed for those ESs. For example, in Lee's et al. (2019) meta-analysis of 18 ESs concerning the “that's not all” technique, one moderator examined was the nature of the target request—whether the request concerned product purchase (*k* = 14) as opposed to volunteering time or donating money (*k* = 4). We analyzed the product-purchase ESs to see the size and consistency of the persuasive advantage given that contingency, but (because of the small number of cases) did not analyze ESs at the other level of that moderator.

#### Two-Moderator Analysis

Third, for each set of effect sizes and moderator variables, results for combinations of two moderating variables were also examined. If such a combination of moderator variables had at least 10 ESs, the mean effect size, 95% CI, and 95% PI were computed for those ESs. For example, in Walter's et al. (2018) meta-analysis concerning the use of humor, 10 ESs were obtained under conditions where the audience's involvement was low and the humor style was satire.

## Results

The 30 message-variation meta-analyses were based on 1,149 studies with 280,591 participants. The mean number of studies per meta-analysis was 38.3; the median was 29. The mean number of participants per meta-analysis was 9,353; the median was 4,422.

A total of 337 mean effect sizes were analyzed: 30 representing main effects of the message variations, 93 representing effects of those variations under one moderating condition, and 214 representing effects under a combination of two moderating conditions.

Table 1 provides results for the main effect of each of the 30 message variations. For moderator effects, parallel results appear in the Supplementary Materials, in Appendix 3 (for one-moderator effects) and Appendix 4 (two-moderator effects)^3^. Appendix 5 gives results for the single largest mean ES for each message variation's one-moderator and two-moderator contingencies, regardless of whether the mean ES was statistically significant; Appendices 6, 7 provide results for statistically significant mean ESs for each message variation for the one-moderator (Appendix 6) and two-moderator (Appendix 7) contingencies, regardless of the size of the mean ES.

**Table 1:** Main effects: mean effect sizes, confidence intervals, and prediction intervals^a^.

**Message variation**	***k***	***N***	**mean *r***	**[95% CI]**	**95% PI**
Sidedness (two-sided vs. one-sided)	107	20,111	−0.002	[−0.035, 0.030]	−0.277, 0.273
Political advertising tone (positive vs. negative)	27	29,035	−0.010	[−0.093, 0.074]	−0.423, 0.406
Appeal framing (gain vs. loss)	165	50,780	0.016	[−0.003, 0.035]	−0.149, 0.180
Language intensity (high vs. low)	15	3,864	0.018	[−0.043, 0.079]	−0.195, 0.230
Sexual content (sexual vs. non-sexual)	11	2,370	0.020	[−0.071, 0.111]	−0.293, 0.329
Evidence type (statistical vs. narrative)	16	1,836	0.044	[−0.051, 0.139]	−0.307, 0.385
Victim description (identifiable vs. non-identifiable)	41	15,967	0.052	[0.003, 0.102]	−0.174, 0.273
Visual material (text-plus-visual vs. text-only)	20	2,452	0.056	[−0.019, 0.131]	−0.240, 0.342
Rhetorical questions (vs. statements)	18	1,950	0.059	[0.001, 0.116]	−0.103, 0.218
Narrative (narrative vs. non-narrative)	34	9,330	0.067	[0.028, 0.105]	−0.107, 0.237
Speaking rate (faster vs. slower)	44	5,645	0.067	[−0.009, 0.143]	−0.387, 0.495
Metaphorical (vs. non-metaphorical)	91	34,783	0.070	[0.047, 0.094]	−0.119, 0.254
Information-source identification (included vs. omitted)	13	2,106	0.072	[0.010, 0.134]	−0.112, 0.251
Cultural tailoring (deep-tailored vs. not-tailored)	67	6,755	0.073	[0.029, 0.118]	−0.223, 0.357
Threat appeal strength (strong vs. weak)	48	6,432	0.100	[0.020, 0.178]	−0.396, 0.551
Recommendation specificity (specific vs. general)	18	11,105	0.101	[0.037, 0.164]	−0.158, 0.347
Conclusion (included vs. omitted)	17	3,110	0.102	[0.028, 0.175]	−0.185, 0.373
Depicted threat severity (high vs. low)	55	8,814	0.116	[0.080, 0.153]	−0.106, 0.327
Humor (humorous vs. non-humorous)	58	10,398	0.119	[0.060, 0.178]	−0.300, 0.500
Argument explicitness (explicit vs. implicit)	18	2,845	0.138	[0.072, 0.202]	−0.113, 0.372
Vividness (vivid vs. pallid)	37	4,468	0.150	[0.090, 0.210]	−0.168, 0.440
“That's not all” (included vs. omitted)	18	937	0.158	[0.054, 0.258]	−0.198, 477
Depicted threat vulnerability (high vs. low)	32	4,376	0.159	[0.085, 0.231]	−0.245, 0.516
“But you are free” (included vs. omitted)	42	22,233	0.175	[0.140, 0.209]	−0.008, 0.346
Argument strength (strong vs. weak)	13	1,684	0.190	[0.109, 0.268]	−0.084, 0.437
Depicted response efficacy (high vs. low)	24	4,348	0.198	[0.126, 0.268]	−0.148, 0.501
Depicted self-efficacy (high vs. low)	21	3,873	0.199	[0.124, 0.272]	−0.138, 0.495
Legitimizing paltry contributions (included vs. omitted)	34	3,181	0.222	[0.170, 0.273]	−0.013, 0.434
Evidence amount (high vs. low)	31	4,697	0.225	[0.182, 0.268]	0.040, 0.395
Disrupt-then-reframe (vs. reframe-only)	14	1,106	0.287	[0.230, 0.341]	0.225, 0.347

a *Note: k, number of effect sizes; CI, confidence interval; PI, prediction interval*.

The magnitudes of the mean ESs are summarized in several ways in Table 2, which reports values for main-effect ESs (all such effects and the statistically significant effects), one-moderator ESs (all such effects, the largest mean ESs for a single moderator level, and the statistically significant effects), and two-moderator ESs (all such effects, the largest mean ESs for a combination of two moderator levels, and the statistically significant effects). For each of those categories, the values reported are the simple unweighted mean absolute-value mean effect size, the simple unweighted median absolute-value mean effect size, the mean upper limit of the 95% CI, and the median upper limit of the 95% CI^4^. These latter two properties are of interest because the upper limit of the 95% CI provides an upper bound on plausible population values.

**Table 2:** Effect size magnitudes: properties of mean effect sizes and confidence intervals^a^.

	***N***	**Mean mean ES (*r*)**	**Median mean ES (*r*)**	**Mean 95% CI upper limit**	**Median 95% CI upper limit**
**Main effects**					
All effects	30	0.109	0.101	[0.169]	[0.159]
Significant mean ESs	22	0.138	0.129	[0.196]	[0.190]
**One moderator**					
All effects	93	0.084	0.065	[0.155]	[0.136]
Significant mean ESs	52	0.121	0.101	[0.187]	[0.160]
Largest mean ESs	15	0.174	0.127	[0.249]	[0.225]
Narrowest PIs	15	0.121	0.078	[0.176]	[0.148]
**Two moderators**					
All effects	214	0.071	0.060	[0.156]	[0.143]
Significant mean ESs	72	0.119	0.103	[0.198]	[0.182]
Largest mean ESs	9	0.154	0.125	[0.234]	[0.216]
Narrowest PIs	9	0.051	0.029	[0.101]	[0.094]

a *Note: N, number of mean effect sizes; ES, effect size; CI, confidence interval*.

The consistency of the ESs (as indicated by PIs) is summarized in several ways in Table 3, which reports values for main-effect ESs (all such effects and the statistically significant effects), one-moderator ESs (all such effects, the largest effects, the statistically significant effects, and the narrowest PIs), and two-moderator ESs (all such effects, the largest effects, the statistically significant effects, and the narrowest PIs). Appendix 8 gives detailed results that specify the narrowest PIs for each message variation's one-moderator and two-moderator contingencies.

**Table 3:** Effect size consistency: properties of prediction intervals^a^.

	***N***	**Mean 95% PI width**	**Median 95% PI width**	**Proportion of zero-straddling PIs**
**Main effects**				
All cases	30	0.540	0.536	0.933
Significant mean ESs	22	0.525	0.513	0.909
**One moderator**				
All cases	93	0.545	0.527	0.968
Significant mean ESs	52	0.516	0.461	0.942
Largest mean ESs	15	0.526	0.560	0.867
Narrowest PIs	15	0.381	0.404	0.867
**Two moderators**				
All cases	214	0.580	0.577	0.986
Significant mean ESs	72	0.564	0.539	0.958
Largest mean ESs	9	0.556	0.583	0.889
Narrowest PIs	9	0.316	0.299	0.889

a *Note: N, number of mean effect sizes; ES, effect size; PI, prediction interval; proportion of zero-straddling PIs, proportion of PIs containing both positive and negative values*.

### Effect Size Magnitudes

#### Main Effects

The 30 main-effect mean ESs ranged from −0.002 to 0.287 (Table 1). The median mean ES for all 30 cases was 0.10, and for the 22 statistically significant cases was 0.13. The corresponding median upper limits of the 95% CI were 0.16 and 0.19 (Table 2).

#### One-Moderator Effects

The 93 one-moderator mean ESs ranged from −0.049 to 0.321 (Appendix 3). The median mean ES for all 93 cases was 0.07, with a median 95% CI upper limit of 0.14^5^.

Of the 30 message variations reviewed, only 15 had any one-moderator levels with at least 10 ESs (i.e., only 15 qualified for analysis). For each of those 15 variations, the largest one-moderator ES was identified (Appendix 5). For those 15 largest mean ESs, the median mean ES was 0.13, with a median 95% CI upper limit of 0.23. For the 52 statistically significant one-moderator mean ESs (Appendix 6), the median mean ES was 0.10, with a median 95% CI upper limit of 0.16 (Table 2). By comparison, for specifically the 15 message variations that contributed to those (largest and statistically significant) one-moderator effects, the median main-effect mean ES was 0.13, with a median 95% CI upper limit of 0.15.

The 15 largest one-moderator mean ESs were also used to get a sense of the potential for individual moderators to yield larger mean effect sizes, by comparing each such mean ES against the corresponding main-effect mean ES. The median amount of increase in the mean ES was 0.06 (e.g., from 0.11 to 0.17).

#### Two-Moderator Effects

The 214 two-moderator mean ESs ranged from −0.071 to 0.276 (Appendix 4). The median mean ES for all 214 cases was 0.06, with a median 95% CI upper limit of 0.14.

Of the 30 message variations reviewed, only nine had any two-moderator levels with at least 10 ESs (i.e., only nine qualified for analysis). For each of those nine variations, the largest two-moderator mean ES was identified (Appendix 5). For those nine largest mean ESs, the median mean ES was 0.13, with a median 95% CI upper limit of 0.22. For the 72 statistically significant two-moderator mean ESs (Appendix 7), the median mean ES was 0.10, with a median 95% CI upper limit of 0.18 (Table 2). By comparison, for specifically the nine message variations that contributed to those (largest and statistically significant) two-moderator effects, the median main-effect ES was 0.07, with a median 95% CI upper limit of 0.11; the median largest one-moderator ES was 0.12, with a median 95% CI upper limit of 0.22.

The nine largest two-moderator mean ESs were also used to get a sense of the potential for moderator combinations to yield larger mean effect sizes, by comparing each such mean ES against the corresponding main-effect mean ES. The median amount of increase in the mean ES was 0.07 (e.g., from 0.08 to 0.15).

### Effect Size Consistency

#### Main Effects

For the 30 main-effect mean ESs (detailed in Table 1), the mean 95% PI width was 0.54; the median was 0.54 (Table 3). The simple unweighted means of the lower and upper bounds of the 95% PIs were, respectively, −0.17 and 0.37; the corresponding medians were −0.16 and 0.37. Twenty-eight (93%) of the PIs included both positive and negative values.

#### One-Moderator Effects

For the 93 one-moderator mean ESs (detailed in Appendix 3), the mean 95% PI width was 0.55; the median was 0.53 (Table 3). The simple unweighted means of the lower and upper bounds of the 95% PIs were, respectively, −0.20 and 0.34; the corresponding medians were −0.20 and 0.32. Ninety (97%) of the PIs included both positive and negative values. For the 15 cases representing the largest one-moderator ESs (detailed in Appendix 3), 13 (87%) of the PIs included both positive and negative values; for the 52 cases representing statistically significant one-moderator ESs (detailed in Appendix 6), 49 (94%) of the PIs included both positive and negative values. By comparison, for specifically the 15 variations that contributed to those one-moderator results, 14 (93%) of the PIs for the main-effect ESs included both positive and negative values.

To get a sense of the potential for individual moderators to yield narrower PIs, two additional “best case” analyses were conducted (see Table 3). First, for each message variation we compared the width of the PI from the one-moderator level that had the narrowest PI (identified in Appendix 8) against the width of the PI for the corresponding main-effect mean ES (as given in Table 1). Across 15 such cases, the median width of the 95% PI was 0.40 for one-moderator mean ESs (median limits of −0.09 and 0.30) and was 0.52 for main-effect mean ESs (median limits of −0.17 and 0.35).

Second, we compared the width of the PI from one-moderator levels that had statistically significant mean ESs (identified in Appendix 6) against the width of the PI for the corresponding main-effect mean ESs (Table 1). Across 52 such cases, the median width of the 95% PI was 0.46 for one-moderator mean ESs (median limits of −0.14 and 0.34) and was 0.52 for main-effect mean ESs (median limits of −0.17 and 0.35).

#### Two-Moderator Effects

For the 214 two-moderator mean ESs (detailed in Appendix 4), the mean 95% PI width was 0.58; the median was 0.58 (Table 3). The simple unweighted means of the lower and upper bounds of the 95% PIs were, respectively, −0.23 and 0.35; the corresponding medians were −0.23 and 0.34. Two hundred eleven (99%) of the PIs included both positive and negative values. For the nine cases representing the largest two-moderator ESs (detailed in Appendix 5), eight (89%) of the PIs included both positive and negative values; for the 72 cases representing statistically significant two-moderator ESs (detailed in Appendix 7), 69 (96%) of the PIs included both positive and negative values. By comparison, for specifically the nine variations that contributed to those two-moderator results, nine (100%) of the PIs for the main-effect ESs included both positive and negative values (Table 1); for the largest one-moderator ESs among those nine variations, eight (89%) of the PIs included both positive and negative values (Appendix 5).

To get a sense of the potential for moderator combinations to yield narrower PIs, two additional “best case” analyses were conducted (see Table 3). First, for each message variation we compared the width of the PI from the two-moderator combination that had the narrowest PI (detailed in Appendix 8) against the width of the PI for the corresponding main-effect mean ES (in Table 1). Across nine such cases, the median width of the 95% PI was 0.30 for two-moderator mean ESs (median limits of −0.12 and 0.18) and was 0.45 for main-effect mean ESs (median limits of −0.17 and 0.27).

Second, we compared the width of the PI from two-moderator levels that had statistically significant mean ESs (identified in Appendix 7) against the width of the PI for the corresponding main-effect mean ESs (Table 1). Across 72 such cases, the median width of the 95% PI was 0.54 for two-moderator mean ESs (median limits of −0.17 and 0.36) and was 0.45 for main-effect mean ESs (median limits of −0.17 and 0.27).

## Discussion

These results support three broad conclusions, which in turn yield two sets of implications.

### Conclusions

Briefly: Message design choices don't make much difference to persuasiveness (message-variation mean effect sizes are small), the effect of a given design choice varies considerably from one application to another (message-variation prediction intervals are wide), and moderator variables have little impact on the size and variability of effects.

#### Small Mean Effects

Persuasive message design choices yield rather modest differences in persuasive effectiveness. The magnitude of the main effects observed in the present analysis (median mean *r* = 0.10) is consistent with the parallel results reported in other reviews: median *r*s of 0.11 (Weber and Popova, 2012) and 0.13 (Rains et al., 2018). But—strikingly—similarly small effects are seen when examining all of the one-moderator effect sizes (median mean *r* = 0.07), the largest one-moderator effect sizes (0.13), the statistically significant one-moderator effect sizes (0.10), all of the two-moderator effect sizes (0.06), the largest two-moderator effect sizes (0.13), and the statistically significant two-moderator effect sizes (0.10)^6^.

Examination of the upper bounds of the 95% CIs is also illuminating, because the upper bound sets a limit on plausible mean (population) values. These results suggest that large mean effects are not to be expected; median upper-bound values never exceed *r* = 0.23. And that's the case no matter whether one looks at main effects (median upper bound of *r* = 0.16), all of the one-moderator effect sizes (0.14), the largest one-moderator effect sizes (0.23), the statistically significant one-moderator effect sizes (0.16), all of the two-moderator effect sizes (0.14), the largest two-moderator effect sizes (0.22), or the statistically significant two-moderator effect sizes (0.18).

And it should not pass unnoticed that the mean effect sizes reported here—as small as they typically are—might nevertheless be inflated. For example, if the studies reviewed in these meta-analyses were affected by outcome-biased reporting (such that studies with smaller effects were less likely to have been available to be included in the meta-analyses), then these mean effect sizes will exaggerate the actual effects (Friese and Frankenbach, 2020; Kvarven et al., 2020; on the difficulties of detecting such biases, see Renkewitz and Keiner, 2019).

In short, there is no evidence that message design choices will characteristically make for large differences in persuasive effectiveness—not even when moderating variables are taken into account.

#### Substantial Variability in Effects

For persuasion message variations, the associated 95% PIs are quite wide, usually including both positive and negative values (i.e., straddling zero). And that's the case no matter whether one looks at all of the main effects (where 93% of the PIs straddle zero), the statistically significant main effects (91%), all of the one-moderator effect sizes (97%), the largest one-moderator effect sizes (87%), the statistically significant one-moderator effect sizes (94%), all of the two-moderator effect sizes (99%), the largest two-moderator effect sizes (89%), or the statistically significant two-moderator effect sizes (96%).

It is important not to be misled here by statistical significance. Knowing that a given mean ES is statistically significantly different from zero (i.e., knowing that its 95% CI excludes zero) does not provide information about the variability of ESs from one implementation to another. A statistically significant mean ES presumably indicates that there is a genuine non-zero population effect—but that speaks only to the location of the average effect, not to the dispersion of individual effect sizes around that mean.

An illustrative example is provided by the message variation contrasting narrative and non-narrative message forms. Under six different one-moderator conditions, there was a statistically significant mean ES favoring narrative messages. But for each of those six, the PI includes negative values—in fact, negative values greater in absolute terms than the positive mean effect (Appendix 6). For instance, when the medium is print, the mean persuasive advantage for narrative forms corresponds to *r* = 0.055, but the lower bound of the PI is −0.127. And the same pattern is observed for the four statistically significant two-moderator mean ESs for this message variation: for each of those four two-moderator combinations, the PI includes negative values greater in absolute terms than the positive mean effect (Appendix 7).

When a PI includes both positive and negative values, the data in hand are consistent with seeing both positive and negative effect sizes in future applications. As these data make clear, it is common for persuasion message-variation PIs to include both positive and negative values. The implication is that one should not be surprised to see one study in which message form A is more persuasive than message form B and another study in which the opposite effect obtains.

In short, there is no evidence that message design choices will characteristically yield effects that are consistent in direction from application to application—not even when moderating variables are taken into account.

#### Minimal Effects of Moderating Factors

One might have imagined that as moderating conditions are specified, the size and consistency of the effects of design choices would noticeably increase. However, as just discussed, these data indicate moderating variables do not have much effect on either the size or the consistency of such effects.

##### Moderators and Effect-Size Magnitudes

To obtain a basis for realistic expectations about the maximum degree to which the consideration of moderating factors might increase the size of the effects of persuasive message design choices, the largest mean ESs observed under moderating conditions are instructive. In that “best case” analysis, there was not much increase in the mean effect size beyond that observed for main effects: when single moderators were considered, the median increase in *r* was 0.06; for combinations of two moderators the median increase was 0.07. And this is the most that might be expected, because these are the largest mean ESs observed under moderating conditions.

Expressed another way: The largest mean ESs found under moderating conditions were indeed numerically larger than those for main effects. But even when two moderators were considered jointly, the median increase (in those largest-mean-ES cases) corresponds to roughly the difference between a mean ES of *r* = 0.10 and one of *r* = 0.17. That is, even the largest ESs observed under moderating conditions are typically not dramatically larger (nor dramatically large)—and these are the effects for the atypically large moderating-factor mean ESs.

To contextualize these best-case mean effects, consider the median mean ESs associated with the moderator conditions that produced the narrowest PIs (Table 2) as compared to the median mean ESs for the corresponding main effects (Table 1). When single moderators were considered, across 15 cases the median mean ES decreased by 0.04 (median mean ESs of 0.08 for the one-moderator cases, 0.12 for the corresponding main effects). When two moderators were considered, across nine cases the median mean ES decreased by 0.04 (median mean ES of 0.03 for the two-moderator cases, 0.07 for the corresponding main effects). That is, the moderating conditions that had the most consistent effect sizes had smaller mean ESs (compared to the mean ESs observed for main effects).

##### Moderators and Effect-Size Consistency

To obtain a basis for realistic expectations about the maximum degree to which the consideration of moderating factors might reduce the width of prediction intervals, the narrowest PIs observed under moderating conditions are instructive. In that “best case” analysis, the width of the narrowest PIs under moderating conditions (Table 3) did not decrease much compared to that observed for main effects (Table 1). When single moderators were considered, across 15 cases the median width was reduced by 0.12 (median widths of 0.40 for the one-moderator cases, 0.52 for the corresponding main effects). When two moderators were considered, across nine cases the median width was reduced by 0.17 (median widths of 0.30 for the two-moderator cases, 0.47 for the corresponding main effects). And this is the most decrease that might be expected, because these are the narrowest PIs observed under moderating conditions.

Expressed another way: The narrowest PIs found under moderating conditions were indeed narrower than those for main effects. But even when two moderators are considered jointly, the median decrease (in those narrowest-PI cases) corresponds to roughly the difference between (say) a PI with limits of −0.18 and 0.28 and a PI with limits of −0.10 and 0.20. That is, even the narrowest PIs observed under moderating conditions are typically not dramatically narrower (or dramatically narrow)—and these are the effects for the atypically narrow moderating-factor PIs^7^.

To contextualize these best-case PI widths, consider the width of the PIs associated with the moderator conditions that produced the largest mean ESs (Table 3) as compared to the width of the PIs for the corresponding main effects (Table 1). When single moderators were considered, across 15 cases the median width increased by 0.04 (median widths of 0.56 for the one-moderator cases, 0.52 for the corresponding main effects). When two moderators were considered, across nine cases the median width increased by 0.11 (median widths of 0.58 for the two-moderator cases,0.47 for the corresponding main effects). That is, the moderating conditions that produced the largest mean ESs had less consistent effect sizes (compared to the consistency observed for main effects).

##### Effects of Joint Moderators

Notably, considering two moderators jointly did not make for substantially larger mean ESs or substantially more consistent effect sizes compared to what is seen when only a single moderator is considered. For example, for each message variation, the largest mean ESs had a median value of 0.13 when one moderator is considered and 0.13 when two moderators are considered (Table 2). Similarly, for each message variation, the narrowest PIs had a median width of 0.40 when one moderator is considered and 0.30 when two moderators are considered (Table 3).

Now perhaps researchers have not (yet) identified the *right* moderator variables, that is, ones that permit identification of circumstances under which large consistent effects can be expected. The moderators that are characteristically explored in the meta-analyses reviewed here are, in a sense, surface-level moderators—ones easily coded given the information provided in research reports. But it may be that more subtle moderators are actually at work.

Consider, for example, theorizing about gain-loss message framing effects. Rothman and Salovey (1997) suggested that the relative persuasiveness of the two message forms would be influenced by the nature of the advocated behavior: where the advocacy subject is disease detection behaviors, loss-framed appeals were predicted to generally be more persuasive than gain-framed appeals, but where the advocacy subject is disease prevention behaviors, gain-framed appeals were predicted to generally be more persuasive than loss-framed appeals.

But subsequently rather subtler approaches have been advanced, such as Bartels's et al. (2010) suggestion that the perceived risk of the behavior, not the type of behavior, moderates the effects of gain-loss message framing. The hypothesis is that when perceived risk is low, gain-framed appeals will be more persuasive than loss-framed appeals, but that when perceived risk is high, loss-framed appeals will have the persuasive advantage. It's not clear just how many relevant studies have been conducted concerning this hypothesis (see Updegraff and Rothman, 2013, p. 671), and at a minimum the evidence is not unequivocally supportive (e.g., Van't Riet et al.'s, 2014; for a review, see Van't Riet et al., 2016). But this hypothesis is an example of how the present conclusions about effect-size magnitudes and consistency might need revision were evidence to accumulate concerning the effects of more refined moderating factors.

Along similar lines: Perhaps large consistent effects are not to be found unless one simultaneously considers three or four or six moderators. This possibility, too, cannot be discounted; the present analysis considers at most two simultaneous moderators and thus is unable to speak to whether large consistent effects appear in other circumstances. Were evidence to accumulate about the effects of more complex moderating conditions, the present conclusions might want modification.

So: The present results are inconsistent with any expectation that consideration of moderating variables will easily identify conditions under which design choices will consistently yield large persuasive advantages. Even when one takes into account the moderating variables that have been examined, message-variation mean effects do not noticeably increase in size and effect sizes do not noticeably increase in consistency. But one should be open to the possibility that future research might underwrite different conclusions.

#### Summary

Persuasion message variations have small and highly variable effects. This might lead some to be discouraged or dispirited, but such reactions would bespeak expectations that turned out to be unrealistic. No one is disappointed to learn that the mass of the electron is extraordinarily small—not unless they expected it would be larger. Similarly here: Any feelings of disappointment reflect (implicit) expectations that have turned out to not be aligned with reality.

We do want to emphasize: The effect sizes analyzed here describe the relative persuasiveness of two messages, not the absolute persuasiveness of a single message. These results do not speak to questions about whether persuasive messages are or can be effective, but rather to questions about the differences between messages in persuasiveness—and thus to the consequences of message design choices.

And it's no good turning away from the apparent facts about the effects of message design choices. The differences in persuasiveness between alternative message forms are rather small and the individual effect sizes are quite variable, even when moderating factors are taken into account. And that, in turn, has implications both for message design and for persuasion research.

### Implications

#### Implications for Message Design

##### Realistic Expectations

Message designers should have realistic beliefs about just how much they can improve effectiveness by their choices. It certainly is possible that in a given application, a message design choice might make a very large difference to effectiveness. But that very same design choice in another application might produce not just a weaker effect, but a *negative* (opposite) effect. And that's true even when the message variation has a statistically significant positive mean effect, and even under well-specified moderating conditions. Message designers should expect that their choices might on average provide incremental improvements, but not consistent dramatic ones.

Similarly, those advising message designers should be modest, cautious, humble. If message-variation effects were substantial and entirely consistent, one could be unreservedly confident in one's recommendations about persuasive message design: “Always choose message form A rather than message form B. Not only will A *always* be more persuasive, it will be a *lot* more persuasive.”

But given the results reported here, advisers will want to be rather restrained, even if there is a statistically significant meta-analytic mean difference in persuasiveness between the message kinds: “You should probably choose message form A rather than message form B, because on average A is more persuasive. However, A is likely to be only a little more persuasive than B, not enormously so. And A will not always be more effective than B—sometimes B will turn out to have been the better choice. So my advice is that you choose A, because you should play the odds. But it's not a sure thing, and it probably won't make a huge difference to persuasiveness.”

Thus, our claim is not that persuasive message design choices don't matter at all. On the contrary, design choices *do* make a difference. After all, there are statistically significant differences between the persuasiveness of various message forms; that is, there are genuine (non-random) differences here. Our point is that the difference made—the difference in persuasiveness between two design options—is not large.

And although message design choices don't make for large differences in persuasiveness, even small differences might, in the right circumstances, be quite consequential (for a classic treatment, see Abelson, 1985; see also Prentice and Miller, 1992). For example, in close elections, a small effect on a small number of voters can be quite decisive (Neuman and Guggenheim, 2011, p. 172–173). More generally, small effects can have significant consequences when examined over time and at scale (Götz et al., 2021). So persuasive message design choices can be important, even though—demonstrably—they make only a small difference to message persuasiveness.

##### Combining Design Features

Imagine a circumstance in which, on average, two-sided messages are more persuasive than one-sided messages *and* gain-framed appeals are more persuasive than loss-framed appeals *and* narratives are more persuasive than non-narratives. Even if each feature individually doesn't boost persuasion that much, a message designer might hope that a two-sided gain-framed narrative could yield a rather large persuasive advantage over other combinations (especially a one-sided loss-framed non-narrative).

However, there is no guarantee that the effects of design features will combine in a simple additive fashion. Direct empirical evidence on this question does not appear to be in hand, but related research—concerning the effects of combining different kinds of interventions (e.g., different behavior change techniques)—suggests a complicated picture. Compared to single interventions, combinations have been found to be more effective (e.g., Huis et al., 2012; Griffiths et al., 2018), less effective (e.g., Jakicic et al., 2016; Wildeboer et al., 2016), and not different in effectiveness (e.g., Luszczynska et al., 2007; Brandes et al., 2019). So it might be the case that at least sometimes, combining message design features will yield larger persuasive advantages, but in the absence of direct evidence, enthusiasm for this prospect should be limited.

#### Implications for Persuasion Research

##### Primary Research

Researchers studying persuasive message effects should design primary research that accommodates these results in two ways. First, researchers will want to plan for larger sample sizes. Only much larger samples will provide sufficient statistical power for detecting the likely (small) population effects. For example, across the 30 main-effect mean ESs, the median effect size (*r*) was 0.10. To have statistical power of 0.80 (two-tailed test, 0.05 alpha) given a population effect of *r* = 0.10 requires 780 participants (Cohen, 1988, p. 93). In the studies included in the meta-analyses reviewed here, the median sample size was 161.

Even if one anticipates larger effect sizes under some moderating condition, a substantial number of participants will be needed. For example, with a population effect of *r* = 0.20, obtaining power of 0.80 (two-tailed test, 0.05 alpha) requires 195 participants. So if one expects an effect size of 0.20 when (say) involvement is high, a design with both high-involvement and low-involvement conditions will require a total of 390 participants. And if one expects to find that size of effect only when (say) involvement is high *and* communicator credibility is high, the design will require 780 participants.

Larger samples are also wanted for another reason. Although it is now widely understood that small sample sizes reduce the chances of finding genuine population effects, it seems not so well-appreciated that low power also increases the chances of false-positive findings (Christley, 2010; see also Button et al., 2013). Thus, a small-sample study that produces a statistically significant effect might well be misleading. In short, both to enhance statistical power and to minimize the chances of false-positive results, persuasion researchers need larger samples.

Second, more studies are needed—especially ones addressing moderating conditions. For all that moderator variables are commonly assumed to be important influences on persuasion message-variation effects, there is remarkably little good evidence concerning moderator effects for most message design choices. Of the 30 message variations reviewed here, only 15 (50%) had sufficient data (*k* ≥ 10) to assess the potential role of single moderators, and only nine (30%) had sufficient data to assess the potential role of two moderators considered jointly. It appears that even among relatively well-studied message variations—sufficiently well-studied to have merited meta-analytic attention—there is commonly not sufficient evidence in hand to speak with any confidence about the role of moderating factors.

The importance of better evidence about moderating conditions is underscored by the commonality with which message-variation 95% PIs include both positive and negative values. To illustrate, consider a biomedical parallel. Suppose a new medical treatment, on average, improves patients' health (there's a statistically significant mean positive effect), but some patients are harmed by the treatment. In such a situation, researchers would presumably want to figure out exactly what leads to those negative outcomes—what conditions foster such results—so as to be able to better indicate when the treatment should be used and when it should be avoided.

Similarly here: Given that the PIs associated with persuasive message variations commonly include both positive and negative values, sound decisions about persuasive message design will require developing an understanding of the conditions that foster the different results (in which message form A is generally more persuasive than message form B, but sometimes the opposite effect occurs). And if thoroughly consistent effects—as represented by a PI that does not straddle zero—are likely to be found only when multiple moderating conditions are specified, then acquisition of better research evidence will be crucial.

##### Replication and Research Synthesis

Much attention has been given in recent years to apparent failures to replicate previous social-scientific research findings (e.g., Open Science Collaboration, 2015), with replication failures sometimes being interpreted as an indication that the originally claimed effect does not really exist. However, the present results suggest that replication failures should be expected to occur routinely in persuasion message effects research. De Boeck and Jeon (2018, p. 766) put it succinctly: “if effects vary from study to study, then replication failures are no surprise” (see, relatedly, Patil et al., 2016). Indeed, given the frequency with which the prediction intervals reported here encompass both positive and negative effects, it would be astonishing if apparent replication failures did *not* occur.

Unfortunately, current research design practices do not appear to acknowledge, or be well-adapted to, this state of affairs. Experimental studies of persuasive message variations typically use a single concrete message to represent an abstract message category. So, for example, an experiment comparing gain-framed and loss-framed appeals will typically have just one example of each (a “single-message” design). But such a research design obviously cannot provide good evidence about whether any observed effects generalize across messages.

Consider the parallel: A researcher hypothesizes that on average men and women differ with respect to some attribute, but designs a study that compares one particular man and one particular woman; that design is plainly not well-suited to provide relevant evidence, because claims about a general category of people require evidence from multiple instances of that category. Similarly: A researcher hypothesizes that on average gain-framed and loss-framed messages differ in persuasiveness, but designs a study that compares one particular gain-framed appeal and one particular loss-framed appeal; that design is plainly not well-suited to provide relevant evidence, because claims about a general message category require evidence from multiple instances. Such single-message designs invite apparent replication failures.

Thus, these results point to the value of multiple-message designs, that is, designs with multiple message pairs representing the contrast of interest (for some discussion, see Kay and Richter, 1977; Jackson and Jacobs, 1983; Thorson et al., 2012; Slater et al., 2015; Reeves et al., 2016). Multiple-message designs effectively have built-in replications of messages, providing a stronger basis for dependable generalizations. Data from such designs can be analyzed in ways that parallel meta-analytic methods, such as treating message as a random factor (Clark, 1973; Fontenelle et al., 1985; Jackson, 1992; Judd et al., 2012, 2017).

Multiple-message designs offer some protection against the possibility that observed effects do not generalize across messages, but they cannot address other potential limitations (e.g., using human samples that are limited in some ways; Henrich et al., 2010). Even so, greater use of such designs plainly could accelerate the process of reaching dependable conclusions about persuasive message effects.

### Summary

Persuasive message designers would like to know of message design choices that will consistently produce a large increase in persuasiveness—either in general (main effects) or contingently (under specified moderating conditions). It's been known for some time that general persuasion message-variation mean effect sizes are not large. The current results suggest that even under well-specified moderator conditions, choosing one message form over another characteristically makes for only a small average difference in persuasiveness. Moreover, such choices do not produce a persuasive advantage consistently—neither generally nor contingently. Message designers and researchers should plan accordingly.

## Data Availability Statement

The original contributions presented in the study are included in the article/Supplementary Material, further inquiries can be directed to the corresponding author/s.

## Author Contributions

DO'K and HH contributed equally to the conception and design of the project, acquisition of data, and analysis and interpretation of data. DO'K wrote the initial draft. DO'K and HH contributed equally to revisions. All authors contributed to the article and approved the submitted version.

## Conflict of Interest

The authors declare that the research was conducted in the absence of any commercial or financial relationships that could be construed as a potential conflict of interest.

## Footnotes

^1^Imagine a circumstance in which (for the same message variation) a meta-analysis with a small number of cases showed a very large mean effect size, while one with a larger number of studies showed a much smaller mean effect size. Everything else being equal, the latter results would enjoy some presumption.

^2^Lull and Bushman (2015) and O'Keefe (1998) each reported more than one meta-analysis. The two specified here were excluded by virtue of an insufficient number of effect sizes, but other meta-analyses from these reports were included.

^3^Only selected moderator results are reported here for two reasons. First, moderator cases are maldistributed across the message variations. Only 15 of the 30 meta-analyses had a moderator-variable level with at least 10 studies and so could contribute mean ESs to the one-moderator analysis. Only nine could contribute to the two-moderator analysis—and of those nine, two provided 135 (63%) of the 214 two-moderator mean ESs (28 from one-sided vs. two-sided messages, 107 from identifiable vs. non-identifiable victim descriptions). Second, the set of all moderator cases has considerable redundancy: multiple different mean ESs were based on the same set of effect sizes. For example, in the meta-analysis of variation of victim descriptions, the 10 effect sizes that contributed to the mean ES for the conjunction of a study location in the United States and the presence of a picture of the victim (*N* = 737, mean ES = 0.116) were the same 10 effect sizes that contributed to five other two-moderator mean ESs (e.g., for a U.S. study location and the absence of a specified monetary goal in the message, for depictions of female victims who belonged to an out-group, and so on).

^4^Absolute-value mean effect sizes were analyzed so as to accommodate the appearance of negative mean effect sizes, as with political advertising tone (Table 1) and non-refutational two-sided messages (Appendix 6).

^5^The median (or mean) mean ESs for moderator effects (whether one moderator or two) are reported for completeness but are arguably uninformative. For example, the mean one-moderator effect for a given message variation will commonly roughly equal the main effect for that variable. (To see this concretely: Imagine the main effect mean ES for a given message variation is *r* = 0.15. And suppose the individual studies divide equally across the two levels of a moderator, with mean ESs of 0.10 and 0.20 for the two levels. Ceteris paribus, the average across those will be about 0.15.) However, examination of subsets of moderator effects—such as the largest ones or the statistically significant ones—is instructive.

^6^Imagine (counterfactually) a measure of the absolute persuasiveness of a given message that was scaled as IQ scores commonly are: *M* = 100, sd = 15. An effect size of *r* = 0.10 corresponds to *d* = 0.20, which is a difference of 3 points on such a scale. That's the difference between scores of 133 and 136, or 81 and 84, or 165 and 168. These are not large differences.

^7^The width of the PI is affected by the number of cases (*k*), such that smaller numbers of cases produce wider PIs. One might therefore suspect that the substantial width of these PIs when moderators are considered is simply a consequence of the inevitably smaller number of cases on which moderator effects are based. However, the relative width of 95% PIs does not change much once 10 cases are in hand (see Meeker et al., 2017, p. 180, Figure 10.1). Given that all the moderator analyses reported here had at least 10 cases, the substantial width of persuasion message variable PIs when moderators are considered is unlikely to be entirely ascribable to the smaller number of cases.
