# Supplementary material for: Message Design Choices Don't Make Much Difference to Persuasiveness and Can't Be Counted On—Not Even When Moderating Conditions Are Specified
Source: Front Psychol. 2021 Jun 29;12:664160. doi: 10.3389/fpsyg.2021.664160 (PMC8275937; doi:10.3389/fpsyg.2021.664160)
Supplement: Supplementary file 1 [file Data_Sheet_1.docx]

**Message Design Choices Don’t Make Much Difference to Persuasiveness and Can’t Be Counted On—Not Even When Moderating Conditions Are Specified**

**SUPPLEMENTAL MATERIALS**

**Appendix 1**: List of excluded meta-analyses

**Appendix 2**: Detailed description of included meta-analyses

**Appendix 3**: All one-moderator effect sizes: Means, confidence intervals, and prediction intervals

**Appendix 4**: All two-moderator effect sizes: Means, confidence intervals, and prediction intervals

**Appendix 5**: Largest one-moderator and two-moderator effect sizes: Mean effect sizes, confidence intervals, and prediction intervals

**Appendix 6**: Statistically significant one-moderator effect sizes: Mean effect sizes, confidence intervals, and prediction intervals

**Appendix 7**: Statistically significant two-moderator effect sizes: Mean effect sizes, confidence intervals, and prediction intervals

**Appendix 8**: Most consistent moderator effects: Mean effect sizes, confidence intervals, and prediction intervals

**APPENDIX 1**

LIST OF EXCLUDED META-ANALYSES

This appendix provides a list of meta-analyses considered for, but excluded from, the analysis. For each meta-analysis, only one reason for exclusion is provided, although some meta-analyses failed to meet multiple inclusion criteria. The reason for exclusion is given as a numerical code, as follows:

**/1/ No appropriate message design variation:** The meta-analyses of interest were ones that reviewed studies in which two alternative message forms were compared (e.g., narrative vs. non-narrative format). Meta-analyses were excluded if the studies reviewed did not examine such comparisons. For example, meta-analyses of communication campaign or intervention effects (e.g., comparing pre- and post-campaign attitudes), meta-analyses of the effects of psychological states (e.g., anger, guilt), and meta-analyses of the effects of communicator variations (e.g., credibility) were all excluded.

**/2/** **No relevant dependent variable:** The dependent variables of interest were outcomes straightforwardly relevant to assessing relative persuasiveness (attitude, intention, behavior). Other outcomes such as affective states (e.g., guilt, fear) or message recall were not relevant.

**/3/ Inappropriate comparison condition:** The studies of interest compared one message form against an alternative message form (e.g., gain-framed vs. loss-framed appeals). Meta-analyses were excluded if their effect sizes did not reflect such comparisons. For example, meta-analyses of studies comparing a message form against a no-message control condition, or of studies comparing a message form against a combination of control conditions (combining, say, no-message controls and alternative-message-form controls) were excluded.

**/4/ Effect sizes not available:** If effect sizes were not available in the report of the meta-analysis or after making contact with the authors, the meta-analysis was excluded.

**/5/** **Too few effect sizes:** A minimum of 10 effect sizes was required for inclusion.

**/6/ Fewer effect sizes than another (included) meta-analysis:** When more than one meta-analysis was available for a given message variable, the one with the largest number of effect sizes was included.

**/7/ Another report of the meta-analysis was available**: Some meta-analyses appeared in multiple reports (e.g., a conference paper and a journal article). As needed, information was combined across such reports. A report is marked here as excluded if some better report (more recent, with more cases, with more detail given, more easily accessed) was available.

Abernethy, A. M., and Franke, G. R. (1996). The information content of advertising: A meta-analysis*. J. Advert*. 25(2), 1-17. doi:10.1080/00913367.1996.10673496 **/2/**

Abrahamse, W., and Steg, L. (2013). Social influence approaches to encourage resource conservation: A meta-analysis. *Glob. Environ. Change*. 23, 1773-1785. doi:10.1016/j.gloenvcha.2013.07.029 **/3/**

Afef, A., Jamel-eddine, G., and Claude, F. (2010). Meta-analysis of incidental advertising and consumer choice. *Int. J. E-Bus. Res*. 10(2), 172-184. **/1/**

Akl, E. A., Oxman, A. D., Herrin, J., Vist, G. E., Terrenato, I., Sperati, F., Costiniuk, C., Blank, D., and Schünemann, H. (2011). Framing of health information messages. *Cochrane Database Syst. Rev.* 2011 Issue 12 (article no. CD006777). doi:10.1002/14651858.CD006777.pub2 **/6/**

Albarracin, D., Johnson, B. T., Fishbein, M., and Muellerleile, P. A. (2001). Theories of reasoned action and planned behavior as models of condom use: A meta-analysis. *Psych. Bull*. 127(1), 142-161. doi:10.1037//0033-2909.127.1.142 **/1/**

Albarracín, D., Kumkale, G. T., and Johnson, B. T. (2004). Influences of social power and normative support on condom use decisions: A research synthesis. *AIDS Care.* 16(6), 700-723. doi:doi:10.1080/09540120412331269558 **/1/**

Allen, M. (1991). Meta-analysis comparing the persuasiveness of one-sided and two-sided messages. *West. J. Commun.* 55, 390-404. doi:10.1080/10570319109374395 **/6/**

Allen, M. (1994). “The persuasive effects of one and two sided messages,” in *Prospects and Precautions in the Use of Meta-Analysis*, ed. M. Allen and R. W. Preiss (Dubuque, IA: Brown and Benchmark), 101-125. **/6/**

Allen, M. (1998). “Comparing the persuasive effectiveness one- and two-sided message,” in *Persuasion: Advances through Meta-Analysis*, ed. M. Allen and R. W. Preiss (New York: Hampton Press), 87-98. **/6/**

Allen, M., and Preiss, R. W. (1990). Using meta-analyses to evaluate curriculum: An examination of selected college textbooks. *Commun. Educ.* 39(2), 103-116. doi:10.1080/03634529009378793 **/2/**

Allen, M., and Preiss, R. G. (1996, May). Comparing the persuasiveness of narrative and statistical evidence using meta-analysis. [Paper presentation]. Intl. Commun. Assoc. annual meeting, Chicago, IL. **/7/**

Allen, M., Preiss, R. W., and Gayle, B. M. (2006). Meta-analytic examination of the base-rate fallacy. *Comm. Res. Rep*. 23(1), 45-51. doi:10.1080/17464090500535863 **/1/**

Allen, M., and Stiff, J. (1994). “An analysis of the sleeper effect,” in *Prospects and Precautions in the Use of Meta-Analysis*, ed. M. Allen and R. W. Preiss (Dubuque, IA: Brown and Benchmark), 185-204. **/7/**

Allen, M., and Stiff, J. B. (1998). “An analysis of the sleeper effect,” in *Persuasion: Advances through Meta-Analysis*, ed. M. Allen and R. W. Preiss (New York: Hampton Press), 175-188. **/1/**

Amos, C., Holmes, G., and Strutton, D. (2008). Exploring the relationship between celebrity endorser effects and advertising effectiveness: A quantitative synthesis of effect size. *Int. J. Advert.* 27(2), 209-234. doi:10.1080/02650487.2008.11073052 **/1/**

Anderson, J. N. (2013, June). Empowering women to increase condom use: A meta-analysis of empowerment HIV prevention intervention effectiveness. [Paper presentation]. Intl. Commun. Assoc. annual meeting, London, UK. **/1/**

Andrews, K. R., Carpenter, C. J., Shaw, A. S., and Boster, F. J. (2008). The legitimization of paltry favors effects: A review and meta-analysis. *Commun. Rep*. 21(2), 59-69. doi:10.1080/08934210802305028 **/6/**

Anker, A., Feeley, T. H., and McCracken, B. (2015, November). Measuring the effectiveness of health campaigns through meta-analysis. [Paper presentation]. Natl. Commun. Assoc. annual meeting, Las Vegas, NV. **/7/**

Anker, A. E., Feeley, T. H., McCracken, B., and Lagoe, C. A. (2016). Measuring the effectiveness of mass-mediated health campaigns through meta-analysis. *J. Health Commun.* 21(4), 439-456. doi:10.1080/10810730.2015.1095820 **/1/**

Argo, J. J., and Main, K. J. (2004). Meta-analyses of the effectiveness of warning labels. *J. Public Policy Mark.* 23(2), 193-208. doi:10.1509/jppm.23.2.193.51400 **/1/**

Armanasco, A. A., Miller, Y. D., Fjeldsoe, B. S., and Marshall, A. L. (2017). Preventive health behavior change text message interventions: A meta-analysis. *Am. J. Prev. Med*. 52(3), 391-402. doi:10.1016/j.amepre.2016.10.042 **/3/**

Aulbach, M. B., Knittle, K., and Haukkala, A. (2019). Implicit process interventions in eating behaviour: A meta-analysis examining mediators and moderators. *Health Psychol. Rev.* 13, 179-208. doi:10.1080/17437199.2019.1571933 **/1/**

Avtgis, T. A. (1998). Locus of control and persuasion, social influence, and conformity: A meta-analytic review. *Psychol. Rep*. 83(3), 899-903. doi:10.2466/pr0.1998.83.3.899 **/1/**

Baker, S. B., Swisher, J. D., Nadenichek, P. E., and Popowicz, C. L. (1984). Measured effects of primary prevention strategies. *Pers. Guid. J.* 62, 459-464. doi:10.1111/j.2164-4918.1984.tb00255.x **/1/**

Bangert-Drowns, R. L. (1988). The effects of school-based substance abuse education: A meta-analysis. *J. Drug. Educ*. 18, 243-264. doi:10.2190/8U40-WP3D-FFWC-YF1U **/1/**

Bauman, K. E. (1997). The effectiveness of family planning programs evaluated with true experimental designs. *Am. J. Public. Health*. 87(4), 666-669. doi:10.2105/AJPH.87.4.666 **/1/**

Beaman, A. L., Cole, C. M., Preston, M., Klentz, B., and Steblay, N. M. (1983). Fifteen years of foot-in-the-door research: A meta-analysis. *Pers. Soc. Psychol. Bull*. 9(2), 181-196. doi:10.1177/0146167283092002 **/1/**

Becker, B. J. (1986). “Influence again: An examination of reviews and studies of gender differences in social influence,” in *The Psychology of Gender: Advances through Meta-Analysis*, ed. J. S. Hyde and M. C. Lynn (Baltimore: Johns Hopkins University Press), 178-209. **/1/**

Benoit, W. L. (1991, November). A meta-analysis of the effects of forewarning on persuasive messages. [Paper presentation]. Speech Commun. Assoc. annual convention, Atlanta, GA. **/7/**

Benoit, W. L. (1994). “Forewarning and persuasion,” in *Prospects and Precautions in the Use of Meta-Analysis*, ed. M. Allen and R. W. Preiss (Dubuque, IA: Brown and Benchmark), 159-184. **/7/**

Benoit, W. L. (1998). “Forewarning and persuasion,” in *Persuasion: Advances through Meta-Analysis*, ed. M. Allen and R. W. Preiss (New York: Hampton Press), 139-154. **/1/**

Benoit, W. L., Hansen, G. J., and Verser, R. M. (2003). A meta-analysis of the effects of watching U. S. presidential debates. *Commun. Monog*. 70(4), 335-350. doi:10.1080/0363775032000179133 **/1/**

Bigman, C. A., Bigsby, E., and Martinez Gonzalez, A. (2018, May). A meta-analysis of the effects of exemplars on emotional, attitudinal, and behavioral intention responses to messages. [Paper presentation]. Intl. Commun. Assoc. annual meeting, Prague, Czech Republic. **/3/**

Bigsby, E., Sanchez, K., and Albarracín, D. (2019, May). Arguments about the value and expectancy of a behavior in fear appeals: Using a model of goals to understand a longstanding problem. [Paper presentation]. Intl. Commun. Assoc. annual meeting, Washington, DC. **/3/**

Bigsby, E., and Wang, N. (2015, May). Message sensation value: A meta-analysis of main effects. [Paper presentation]. Intl. Commun. Assoc. annual meeting, San Juan, Puerto Rico. **/5/**

Bolkan, S., and Rains, S. A. (2015, November). The legitimization of paltry contributions (LPC) as a compliance gaining technique: A meta-analysis testing three explanations. [Paper presentation]. Natl. Commun. Assoc. annual meeting, Las Vegas, NV. **/7/**

Boster, F. J., Cruz, S., Manata, B., DeAngelis, B. N., and Zhuang, J. (2016). A meta-analytic review of the effect of guilt on compliance. *Soc. Influ*. 11(1), 54-67*.* doi:10.1080/15534510.2016.1142892 **/1/**

Boster, F. J., and Levine, K. J. (1997, May). The impact of the channel variable on persuasive messages: A meta-analytic review. [Paper presentation]. Intl. Commun. Assoc. annual meeting, Montreal, Canada. **/1/**

Boster, F. J., and Mongeau, P. (1984). Fear-arousing persuasive messages. *Ann. Int. Commun. Assoc.* 8*,* 330-375. doi:10.1080/23808985.1984.11678581

[also cited as: Boster, F. J., and Mongeau, P. (1984). Fear-arousing persuasive messages. *Communication Yearbook, 8*, 330-375.]

[also cited as: Boster, F. J., and Mongeau, P. (1984). “Fear-arousing persuasive messages,” in *Communication Yearbook 8*, ed. R. N. Bostrom (Beverly Hills, CA: Sage), 330-375.] **/6/**

Braddock, K., and Dillard, J. P. (2012, November). The effect of narrative on beliefs, attitudes, and intentions: A meta-analysis. [Paper presentation]. Natl. Commun. Assoc. annual meeting, Orlando, FL. **/7/**

Braddock, K. H., and Dillard, J. P. (2015, May). The effect of narrative on beliefs, attitudes, intentions, and behavior: A meta-analysis. [Paper presentation]. Intl. Commun. Assoc. annual meeting, San Juan, Puerto Rico. **/7/**

Braddock, K., and Dillard, J. P. (2016). Meta-analytic evidence for the persuasive effect of narratives on beliefs, attitudes, intentions, and behaviors. *Commun. Monogr.* 83(4), 446-467*.* doi:10.1080/03637751.2015.1128555 **/3/**

Brentar, J. E. (2001). The role of music and affect in persuasion. [Doctoral dissertation]. [Madison, (WI)]: University of Wisconsin – Madison). ProQuest no. 3012562. **/1/**

Brentar, J. E., Dillard, J. P., and Smith, B. A. (1997, May). Message-irrelevant affect and persuasion: A meta-analysis. [Paper presentation]. Intl. Commun. Assoc. annual meeting, Montreal, Canada. **/1/**

Brown, S. P., and Stayman, D. M. (1992). Antecedents and consequences of attitude toward the ad: A meta-analysis. *J. Consum. Res*. 19(1), 34-51. doi:10.1086/209284 **/1/**

Brown, S. P., Homer, P. M., and Inman, J. J. (1998). A meta-analysis of relationships between ad-evoked feelings and advertising responses. *J. Mark. Res*. 35(1), 114-126. doi:10.1177/002224379803500111 **/1/**

Brugman, B. C., Burgers, C., and Vis, B. (2018, May). Metaphorical framing and political decision-making: A meta-analysis. [Paper presentation]. Intl. Commun. Assoc. annual meeting, Prague, Czech Republic. **/7/**

Bruvold, W. H. (1993). A meta-analysis of adolescent smoking prevention programs. *Am. J. Public Health* 83(6), 872-880. doi:10.2105/AJPH.83.6.872 **/1/**

Buller, D. B. (1986). Distraction during persuasive communication: A meta-analytic review. *Commun. Monogr*. 53(2), 91-114. doi:10.1080/03637758609376130 **/1/**

Buller, D. B., and Hall, J. R. (1994). “The effects of distraction during persuasion,” in *Prospects and Precautions in the Use of Meta-Analysis*, ed. M. Allen and R. W. Preiss (Dubuque, IA: Brown and Benchmark Eds), 205-234. **/7/**

Buller, D. B., and Hall, J. R. (1998). “The effects of distraction during persuasion,” in *Persuasion: Advances through Meta-Analysis*, ed. M. Allen and R. W. Preiss (New York: Hampton Press), 155-173. **/1/**

Burger, J. M. (1999). The foot-in-the-door compliance procedure: A multiple-process analysis and review. *Pers. Soc. Psychol. Rev*. 3(4), 303-325. doi:10.1207/s15327957pspr0304_2 **/1/**

Burger, J. M., and Caputo, D. (2015). The low-ball compliance procedure: A meta-analysis. *Soc. Influ*. 10(4), 214-220. doi:10.1080/15534510.2015.1049203 **/1/**

Burrell, N. A., and Koper, R. J. (1994). “The efficacy of powerful/powerless language on persuasiveness/credibility: A meta-analytic review,” in *Prospects and Precautions in the Use of Meta-Analysis*, ed. M. Allen and R. W. Preiss (Dubuque, IA: Brown and Benchmark), 235-255. **/7/**

Burrell, N. A., and Koper, R. J. (1998). “The efficacy of powerful/powerless language on attitudes and source credibility,” in *Persuasion: Advances through Meta-Analysis*, ed. M. Allen and R. W. Preiss (New York: Hampton Press), 203-215. **/5/**

Carey, K. B., Scott-Sheldon, L. A. J., Carey, M. P., and DeMartini, K. S. (2007). Individual-level interventions to reduce college student drinking: A meta-analytic review. *Addict. Behav*. 32(11), 2469–2494. doi:10.1016/j.addbeh.2007.05.004 **/1/**

Carey, K. B., Scott-Sheldon, L. A., Elliott, J. C., Garey, L., and Carey, M. P. (2012). Face-to-face versus computer-delivered alcohol interventions for college drinkers: A meta-analytic review, 1998 to 2010. *Clin. Psychol. Rev.* 32(8), 690-703. doi:10.1016/j.cpr.2012.08.001 **/1/**

Carey, R. (2014). The impact of threat appeals on risky driving behaviours. [Doctoral dissertation]. [Galway, Ireland]: National University of Ireland. **/3/**

Carey, R. N., McDermott, D. T., and Sarma, K. M. (2013). The impact of threat appeals on fear arousal and driver behavior: A meta-analysis of experimental research 1990–2011. *PLoS One* 8(5): e62821. doi:10.1371/journal.pone.0062821 **/3/**

Carpenter, C. J. (2009, April). A meta-analysis of the effectiveness of the disrupt-then-reframe compliance gaining technique. [Paper presentation]. Centr. Stat. Commun. Assoc. annual meeting, St. Louis, MO. **/7/**

Carpenter, C. J. (2011, November). A meta-analysis of the functional matching effect based on functional attitude theory. [Paper presentation]. Natl. Commun. Assoc. annual meeting, New Orleans, LA. **/7/**

Carpenter, C. J. (2012, November). A meta-analysis of the effectiveness of the “but you are free” compliance-gaining technique. [Paper presentation]. Natl. Commun. Assoc. annual meeting, Orlando, FL. **/7/**

Carpenter, C. J. (2012). A meta-analysis of the functional matching effect based on functional attitude theory. *South. Commun. J.* 77(5), 438-451. doi:10.1080/1041794X.2012.699989 **/1/**

Carr, R. M., Prestwich, A., Kwasnicka, D., Thøgersen-Ntoumani, C., Gucciardi, D. F., Quested, E., Hall, L. H., and Ntoumanis, N. (2019). Dyadic interventions to promote physical activity and reduce sedentary behaviour: Systematic review and meta-analysis. *Health Psychol. Rev*. 13(1), 91-109. doi:10.1080/17437199.2018.1532312 **/1/**

Carron, A. V., Hausenblas, H. A., and Mack, D. (1996). Social influence and exercise: A meta-analysis. *J. Sport Exerc. Psychol*. 18(1), 1-16. doi:10.1123/jsep.18.1.1 **/1/**

Carveth, R., and Desmond, R. (1999, May). The effects of advertising on children and adolescents: A meta-analysis. [Paper presentation]. Intl. Commun. Assoc. annual meeting, San Francisco, CA. **/1/**

Casey, M., Timmerman, L. M., Allen, M., Krahn, S., and Laplant, K. (2007, November). A meta-analysis examining the relationship between self and response efficacy of condom use and the attitudes, behavioral intentions, and condom-use behaviors. [Paper presentation]. Natl. Commun. Assoc. annual meeting, Chicago, IL. **/7/**

Casey, M. K., Timmermann, L., Allen, M., Krahn, S., and Turkiewicz, K. L. (2009). Response and self-efficacy of condom use: A meta-analysis of this important element of AIDS education and prevention. *South. Commun. J*. 74(1), 57-78. doi:10.1080/10417940802335953 **/1/**

Chan, M.-P. S., Jones, C. R., Jamieson, K. H., and Albarracín, D. (2017). Debunking: A meta-analysis of the psychological efficacy of messages countering misinformation. *Psychol. Sci.* 28(11), 1531-1546. doi:10.1177/0956797617714579 **/1/**

Chon, K. Y., and Lee, J.-G. (2015). A meta-analysis of humor in advertising in Korea. *Korean J Journal. Commun. Stud*. 59(6), 477-504. **/4/**

Chu, A. H. Y. (2018). Physical activity, sedentary behaviour and health: From measurements to recommendations. [Doctoral dissertation]. [Singapore]: National University of Singapore. ProQuest no. 10907388. **/7/**

Chu, A. H. Y., Ng, S. H. X., Tan, C. S., Win, A. M., Koh, D. and Müller-Riemenschneider F. (2016). A systematic review and meta-analysis of workplace intervention strategies to reduce sedentary time in white-collar workers. *Obes. Rev*. 17(5), 467-481. doi:10.1111/obr.12388 **/1/**

Collins, D., Johnson, K., and Becker, B. J. (2007) A meta-analysis of direct and mediating effects of community coalitions that implemented science-based substance abuse prevention interventions. *Subst. Use Misuse*. 42(6), 985-1007. doi:10.1080/10826080701373238 **/1/**

Compeau, L. D., and Grewal, D. (1998). Comparative price advertising: An integrative review. *J. Public Policy Mark*. 17(2), 257-273. https://www.jstor.org/stable/30000775 **/4/**

Conn, V. S., Hafdahl, A. R., LeMaster, J. W., Ruppar, T. M., Cochran, J. E., and Nielsen, P. J. (2008). Meta-analysis of health behavior change interventions in type 1 diabetes. *Am. J. Health Behav.* 32, 315-329. doi:10.5993/AJHB.32.3.9 **/1/**

Conn, V. S., Hafdahl, A. R., and Mehr, D. R. (2011). Interventions to increase physical activity among healthy adults. *Am. J Public Health* 101, 751-758. doi:10.2105/AJPH.2010.194381 **/1/**

Conn, V. S., Valentine, J. C., and Cooper, H. M. (2002). Interventions to increase physical activity among aging adults: A meta-analysis. *Ann. Behav. Med*. 24, 190-200. doi:10.1207/S15324796ABM2403_04 **/1/**

Conroy, D., and Hagger, M. S. (2018). Imagery interventions in health behavior: A meta-analysis. *Health Psychol*. 37*,* 668-679. doi:10.1037/hea0000625 **/1/**

Costley, C. L. (1988). Meta-analysis of involvement research. *Adv. Consum. Res.* 15, 554-562. [no doi] **/1/**

Covey, J., Rosenthal-Stott, H. E., and Howell, S. J. (2016). A synthesis of meta-analytic evidence of behavioral interventions to reduce HIV/STIs. *J. Behav. Med*. 39, 371-385. doi:10.1007/s10865-016-9714-1 **/1/**

Cox, E. P., III, Wogalter, M. S., Stokes, S. L., and Murff, E. J. T. (1997). Do product warnings increase safe behavior? A meta-analysis. *J. Public Policy Mark*. 16, 195-204. doi:10.1177//074391569701600201 **/3/**

Cross, A., and Sheffield, D. (2019). Mental contrasting for health behaviour change: A systematic review and meta-analysis of effects and moderator variables. *Health Psychol. Rev*. 13, 209-225. doi:10.1080/17437199.2019.1594332 **/1/**

Cruz, M. G. (1998). “Explicit and implicit conclusions in persuasive messages,” in *Persuasion: Advances through Meta-Analysis*, ed. M. Allen and R. W. Preiss (New York: Hampton Press), 217-230. **/6/**

Cugelman, B., Thelwall, M., and Dawes, P. (2011). Online interventions for social marketing health behavior change campaigns: A meta-analysis of psychological architectures and adherence factors. *J. Med. Internet Res*. 13, 84-107. doi:10.2196/jmir.1367 **/1/**

Cuijpers, P. (2002). Peer-led and adult-led school drug prevention: A meta-analytic comparison. *J. Drug Educ*. 32, 107-119. doi:10.2190//LPN9-KBDC-HPVB-JPTM **/1/**

Cushing, C. C. and Steele R. G. (2010). A meta-analytic review of eHealth interventions for pediatric health promoting and maintaining behaviors. *J. Pediatr. Psychol*. 35*,* 937-949. doi:10.1093/jpepsy/jsq023 **/1/**

Dai, M., and Harrington, N. G. (2018, May). Does the internet help health campaigns? A meta-analysis of the effects of internet supplemented health campaigns on behavior change in the United States. [Paper presentation]. Intl. Commun. Assoc. annual meeting, Prague, Czech Republic. **/1/**

De Leeuw, E., Callegaro, M., Hox, J., Korendijk, E., and Lensvelt-Mulders, G. (2007). The influence of advance letters on response in telephone surveys: A meta-analysis. *Public Opin. Q*. 71, 413-443. doi:10.1093/poq/nfm014 **/1/**

Derzon, J. H., and Lipsey, M. W. (2002). A meta-analysis of the effectiveness of mass-communication for changing substance-use knowledge, attitudes, and behavior, in *Mass Media and Drug Prevention: Classic and Contemporary Theories and Research*, ed. W. D. Crano and M. Burgoon (Mahwah, NJ: Lawrence Erlbaum), 231-258. **/1/**

Dillard, J. P., Hunter, J. E., and Burgoon, M. (1984). Sequential-request strategies: Meta-analysis of foot-in-the-door and door-in-the-face. *Hum. Commun. Res*. 10, 461-488. doi:10.1111/j.1468-2958.1984.tb00028.x **/1/**

Dillard, J. P., and Li, S. S. (2019, May). How scary are threat appeals? Evaluating the intensity of fear in experimental research. Intl. Commun. Assoc. annual meeting, Washington, DC. **/2/**

Dunst, C. J. (2014). Meta-analysis of the effects of puppet shows on attitudes toward and knowledge of individuals with disabilities. *Except. Child*. 80, 136-148. doi:10.1177/001440291408000201 **/1/**

Durantini, M. R., Albarracín, D., and Mitchell, A. L. (2006). Conceptualizing the influence of social agents of behavior change: A meta-analysis of the effectiveness of HIV-prevention interventionists for different groups. *Psychol. Bull*. 132, 212-248. doi:10.1037/0033-2909.132.2.212 **/1/**

Eagly, A. H., and Carli, L. L. (1981). Sex of researchers and sex-typed communications as determinants of sex differences in influenceability: A meta-analysis of social influence studies. *Psychol. Bull*. 90, 1-20. doi:10.1037/0033-2909.90.1.1 **/1/**

Earl, A., and Albarracín, D. (2007). Nature, decay, and spiraling of the effects of fear-inducing arguments and HIV counseling and testing: A meta-analysis of the short- and long-term outcomes of HIV-prevention interventions. *Health Psychol*. 26(4), 496-506. doi:10.1037/0278-6133.26.4.496 **/3/**

Eckes, T., and Six, B. (1994). Fakten und fikition in der einstellungs-verhalten-forschung: Eine meta-analyse [Fact and fiction in attitude-behavior research: A meta-analysis]. *Z. Soz.psychol.* 25, 253-271. [no doi] **/1/**

Eisend, M. (2006). Two-sided advertising: A meta-analysis. *Int. J. Res. Mark*. 23, 187-198. doi:10.1016/j.ijresmar.2005.11.001 **/3/**

Eisend, M. (2007). Understanding two-sided persuasion: An empirical assessment of theoretical approaches. *Psychol. Mark*. 24, 615-640. doi:10.1002/mar.20176 **/3/**

Eisend, M. (2007). A meta-analysis of humor effects in advertising. *North Am. Adv. Consum. Res* 34 320-323. **/7/**

Eisend, M. (2009). A meta-analysis of humor in advertising. *J Acad. Mark. Sci*. 37, 191-203. doi:10.1007/s11747-008-0096-y **/6/**

Eisend, M. (2010). A meta-analysis of gender roles in advertising. *J. Acad. Mark. Sci*. 38, 418-440. doi:10.1007/s11747-009-0181-x **/2/**

Eisend, M. (2017). The third-person effect in advertising: A meta-analysis. *J. Advert*. 46, 377-394. doi:10.1080/00913367.2017.1292481 **/1/**

Eisend, M., and Hermann, E. (2019). Consumer responses to homosexual imagery in advertising: A meta-analysis. *J. Advert*. 48(4), 380-400. doi:10.1080/00913367.2019.1628676 **/3/**

Eisend, M., and Kuster, F. (2011). The effectiveness of publicity versus advertising: A meta-analytic investigation of its moderators, *J. Acad. Mark. Sci*. 39*,* 906-921. doi:10.1007/s11747-010-0224-3 **/1/**

Ennett, S. T., Tobler, N. S., Ringwald, C. L. , and Flowelling, R. L. (1994). How effective is drug abuse resistance education? A meta-analysis of Project DARE outcome evaluations. *Am. J.* *Public Health* 84, 1394-1401. doi:10.2105/AJPH.84.9.1394 **/1/**

Feeley, T. H., Anker, A. E., and Aloe, A. M. (2012). The door-in-the-face persuasive message strategy: A meta-analysis of the first 35 years. *Commun. Monogr.* 79, 316-343. doi:10.1080/03637751.2012.697631 **/1/**

Feeley, T. H., and Moon, S. (2009, May). A meta-analysis of the effects of public education campaigns on the promotion of organ donation. [Paper presentation]. Intl. Commun. Assoc. annual meeting, Chicago, IL. **/7/**

Feeley, T. H., and Moon, S. (2009). A meta-analytic review of communication campaigns to promote organ donation. *Commun. Rep*. 22, 63-73. doi:10.1080/08934210903258852 **/1/**

Fern, E. F., Monroe, K. B., and Avila, R. A. (1986). Effectiveness of multiple request strategies: A synthesis of research results. *J. Mark. Res*. 23, 144‑152. doi:10.1177%2F002224378602300206 **/1/**

Finitsis, D. J., Pellowski, J. A., and Johnson, B. T. (2014). Text message intervention designs to promote adherence to antiretroviral therapy (ART): a meta-analysis of randomized controlled trials. *PLoS One* 9, article no.e88166. doi:10.1371/journal.pone.0088166 **/1/**

Fischer, M., and Huber, F. (2015). “The picture communication effect: A meta-analysis,” in *Global perspectives in marketing for the 21st century: Proceedings of the 1999 World Marketing Congress*, ed. A. K. Manrai and H. L. Meadow (Cham, Switzerland: Springer), 65-68, doi:10.1007/978-3-319-17356-6_20 **/4/**

Flores, S. A., and Hartlaub, M. G. (1998). Reducing rape-myth acceptance in male college students: A meta-analysis of intervention studies. *J. Coll. Stud. Dev*. 39, 438-448. [no doi] **/1/**

Floyd, K., Freling, R., Alhoquil, S. A., Cho, H. Y., and Freling, T. (2014). How online product reviews affect retail sales: A meta-analysis. *J. Retail*. 90(2), 217-232. doi:10.1016/j.jretai.2014.04.004 **/1/**

Floyd, D. L., Prentice-Dunn, S., and Rogers, R. W. (2000). A meta-analysis of research on protection motivation theory. *J. Appl. Soc. Psychol*. 30, 407-429. doi:10.1111/j.1559-1816.2000.tb02323.x **/4/**

Folkvord, F., and van 't Riet, J. (2018). The persuasive effect of advergames promoting unhealthy foods among children: A meta-analysis. *Appetite* 129, 245-251. doi:10.1016/j.appet.2018.07.020 **/1/**

Franklin, C., Grant, D., Corcoran, J., Miller, P. O., and Bultman, L. (1997). Effectiveness of prevention programs for adolescent pregnancy: A meta-analysis. *J. Marriage Fam*. 59, 551-567. doi:10.2307/353945 **/1/**

Freling, R. E. (2017). Understanding influences and biases in decision making: Three meta-analyses on information processing. [Doctoral dissertation]. [Dallas (TX)]: University of Texas at Dallas. ProQuest no. 10759978. **/4/**

Freling, T. H., Vincent, L. H., and Henard, D. H. (2014). When not to accentuate the positive: Re-examining valence effects in attribute framing. *Organ. Behav. Hum. Decis. Process*. 124(2), 95-109. doi:10.1016/j.obhdp.2013.12.007 **/4/**

Gallagher, K. M., and Updegraff, J. A. (2012). Health message framing effects on attitudes, intentions, and behavior: A meta-analytic review. *Ann. Behav. Med*. 43, 101-116. doi.10.1007/s12160-011-9308-7 **/6/**

Gayle, B. M., Preiss, R. W., and Allen, M. (1994). “The persuasive effects of rhetorical questions,” in *Prospects and Precautions in the Use of Meta-Analysis*, ed. M. Allen and R. W. Preiss (Dubuque, IA: Brown and Benchmark), 315-337. **/7/**

Gillison, F. B., Rouse, P., Standage, M., Sebire, S. J., and Ryan, R. M. (2019). A meta-analysis of techniques to promote motivation for health behaviour change from a self-determination theory perspective. *Health Psychol. Rev*. 13, 110-130. doi:10.1080/17437199.2018.1534071 **/1/**

Good, A. and Abraham, C. (2007). Measuring defensive responses to threatening messages: A meta-analysis of measures. *Health Psychol. Rev*. 1, 208-229. doi:10.1080/17437190802280889 **/2/**

Gordon, R. A. (1996). Impact of ingratiation on judgments and evaluations: a meta-analytic investigation. *J. Pers. Soc. Psychol*. 71, 54–70. doi:10.1037/0022-3514.71.1.54 **/1/**

Grewal, D., Kavanoor, S., Fern, E. F., Costley, C., and Barnes, J. (1997). Comparative versus noncomparative advertising: A meta-analysis. *J. Market*. 61(4), 1-15. doi:10.1177/002224299706100401 **/4/**

Griffiths, S. E., Parsons, J., Naughton, F., Fulton, E. A., Tombor, I., and Brown, K. E. (2018). Are digital interventions for smoking cessation in pregnancy effective? A systematic review and meta-analysis. *Health Psychol. Rev*. 12*,* 333-356. doi:10.1080/17437199.2018.1488602 **/1/**

Guadagno, R. E., Okdie, B. M., Sagarin, B. J., DeCoster, J., and Rhoads, K. V. L. (2010, January). A meta-analysis on the impact of vividness on persuasion. [Poster presentation]. Soc. Pers. Soc. Psychol. annual meeting, Las Vegas, NV. **/7/**

Guadagno, R. E., Okdie, B. M., Sagarin, B. J., DeCoster, J., and Rhoads, K. V. L. (2013). Understanding the elusive vividness effect: A meta-analysis on vividness and persuasion. Unpublished manuscript. **/4/**

Hagenzieker, M. P., Bijleveld, F. D., and Davidse, R. J. (1997). Effects of incentive programs to stimulate safety belt use: A meta-analysis. *Accid. Anal. Prev*. 29, 759-777. doi:10.1016/S0001-4575(97)00045-6 **/1/**

Hagger, M. S., Chatzisarantis, N. L. D., and Biddle, S. J. H. (2002). A meta-analytic review of the theories of reasoned action and planned behavior in physical activity: Predictive validity and the contribution of additional variables. *J. Sport Exerc. Psychol*. 24, 3-32. [no doi] **/1/**

Hale, S. L. (1998, April). Attack messages and their effects on judgments of political candidates: A random-effects meta-analytic review. [Paper presentation]. Midwest Polit. Sci. Assoc. annual meeting, Chicago, IL. **/6/**

Hamilton, M. A., and Hunter, J. E. (1994). “The effect of language intensity on receiver attitudes toward message, source, and topic,” in *Prospects and Precautions in the Use of Meta-Analysis*, ed. M. Allen and R. W. Preiss (Dubuque, IA: Brown and Benchmark), 257-314. **/7/**

Hausenblas, H. A., Carron, A. V., and Mack, D. E. (1997). Application of the theories of reasoned action and planned behavior to exercise behavior: A meta-analysis. *J. Sport Exerc. Psychol*. 19, 36-51. [no doi] **/1/**

He, X. (2016). The impact of self-affirmation on defensive processing of health messages: A meta-analysis. [Doctoral dissertation]. [Twin Cities (MN)]: University of Minnesota. ProQuest no. 1807951064. **/1/**

Head, K. J., Noar, S. M., Iannarino, N. T., and Harrington, N. G. (2012, November). Efficacy of text messaging-based interventions for health promotion: A meta-analysis. [Paper presentation]. Natl. Commun. Assoc. annual meeting, Orlando, FL. **/7/**

Head, K. J., Noar, S. M., Iannarino, N. T., and Harrington, N. G. (2013). Efficacy of text messaging-based interventions for health promotion: A meta-analysis. *Soc. Sci. Med.* 97, 41-48. doi:10.1016/j.socscimed.2013.08.003 **/1/**

Higgins, C. A., Judge, T. A., and Ferris, G. R. (2003). Influence tactics and work outcomes: A meta-analysis. *J. Organ. Behav*. 24, 89-106. doi:10.1002/job.181 **/1/**

Huang, Y., and Shen, F. (2016). Effects of cultural tailoring on persuasion in cancer communication: A meta-analysis. *J. Commun*. 66, 694-715. doi.10.1111/jcom.12243 **/6/**

Huang, Z., Wang, M., Fu, L., Fang, Y., Hao, J., Tao, F., and Tu, C. (2013). Intervention to increase condom use and HIV testing among men who have sex with men in China: A meta-analysis. *AIDS Res. Hum. Retroviruses* 29, 441-448. doi:10.1089/aid.2012.0151 **/1/**

Huis, A., van Achterberg, T., de Bruin, M., Grol, R., Schoonhoven, L., and Hulscher, M. (2012). A systematic review of hand hygiene improvement strategies: A behavioural approach. *Implement. Sci*. 7*,* 92. doi:10.1186/1748-5908-7-92 **/1/**

Hullett, C. R. (2005). The impact of mood on persuasion: A meta-analysis. *Commun. Res. 32*, 423-442. doi:10.1177/0093650205277317 **/1/**

Hung, C.-L. (2017). A meta-analysis of the evaluations of social marketing interventions addressing smoking, alcohol drinking, physical activity, and eating. [Doctoral dissertation]. [Bloomington (IN)]: Indiana University. ProQuest no. 10272371. **/1/**

Iverson, B. K., and Levy, S. R. (1982). Using meta-analysis in health education research. *J. Sch Health* 52, 234-239. [mo doi] **/1/**

Jeong, M., and Bae, E. R. (2015, May). A meta-analysis of the effect of interpersonal communication on health outcomes in the context of mass media campaigns. [Paper presentation]. Intl. Commun. Assoc. annual meeting, San Juan, Puerto Rico. **/1/**

Johnson, B. T., and Eagly, A. H. (1989). Effects of involvement on persuasion: A meta-analysis. *Psychol. Bull*. 106, 290-314. doi:10.1037/0033-2909.106.2.290 **/1/**

Johnson, B. T., Michie, S., and Snyder, L. B. (2014). Effects of behavioral intervention content on HIV prevention outcomes: A meta-review of meta-analyses. *J. Acquir. Immune Defic. Syndr.* 66(Suppl. 3), S259-S270. doi:10.1097/QAI.0000000000000235 **/1/**

Kalichman, S. C., Carey, M. P., and Johnson, B. T. (1996). Prevention of sexually transmitted HIV infection: A meta-analytic review of the behavioral outcome literature. Ann. Behav. Med., 18, 6-15. https://doi-org.proxy.library.uu.nl/10.1007/BF02903934 **/1/**

Kang, M., Marshall, S. J., Barreira, T. V., and Lee, J. O. (2009). Effect of pedometer-based physical activity interventions: A meta-analysis. *Res. Q. Exerc. Sport* 80, 648-655. doi:10.1080/02701367.2009.10599604 **/1/**

Kareklas, I. (2010). A quantitative review and extension of racial similarity effects in advertising. [Doctoral dissertation]. [Mansfield (CT)]: University of Connecticut. AAT 3415548 **/1/**

Karlin, B., Zinger, J. F., and Ford, R. (2015). The effects of feedback on energy conservation: A meta-analysis. *Psychol. Bull*. 141, 1205-1227. doi:10.1037/a0039650 **/3/**

Kassavou, A., and Sutton, S. (2018). Automated telecommunication interventions to promote adherence to cardio-metabolic medications: Meta-analysis of effectiveness and meta-regression of behaviour change techniques. *Health Psychol. Rev*. 12, 25-42. doi:10.1080/17437199.2017.1365617 **/1/**

Keller, P. A., and Lehman, D. R. (2008). Designing effective health communications: A meta-analysis. *J. Public Policy Market*. 27, 117-130. doi:10.1509/jppm.27.2.117 **/3/**

Kelley, K., Bond, R., and Abraham, C. (2001). Effective approaches to persuading pregnant women to quit smoking: A meta-analysis of intervention evaluation studies. *Br. J. Health Psychol*. 6, 207-228. doi:10.1348/135910701169160 **/1/**

Kelley, K., Bond, R., and Abraham, C. (2001). Effective approaches to persuading pregnant women to quit smoking: A meta-analysis of intervention evaluation studies. *Br. J. Health Psychol.* 6, 207-228. doi:10.1348/135910701169160 **/1/**

Kim, M.-S., and Hunter, J. E. (1993). Attitude-behavior relations: A meta-analysis of attitudinal relevance and topic. *J. Commun.* 43(1), 101-142. doi:10.1111/j.1460-2466.1993.tb01251.x **/1/**

Kim, N., Stanton, B., Li, X., Dickersin, K., and Galbraith, J. (1997). Effectiveness of the 40 adolescent AIDS-risk reduction interventions: A quantitative review. *J. Adolesc. Health* 20, 204-215. doi:10.1016/S1054-139X(96)00169-3 **/1/**

Kim, S. Y., Allen, M., Preiss, R. W., and Peterson, B. (2014). Meta-analysis of counterattitudinal advocacy data: Evidence for an additive cues model. *Commun. Q.* 62, 607–620. doi:10.1080/01463373.2014.949385 **/1/**

Kirby, D., Short, L., Collins, J., Rugg, D., Kolbe, L., Howard, M., Miller, B., Sonenstein, F., and Zabin, L. S. (1994). School-based programmes to reduce sexual risk behaviors: A review of effectiveness. *Public Health Rep*. 109, 339-360. https://www.jstor.org/stable/4597597 **/1/**

Knittle, K., Nurmi, J., Crutzen, R., Hankonen, N., Beattie, M., and Dombrowski, S. U. (2018). How can interventions increase motivation for physical activity? A systematic review and meta-analysis. *Health Psychol. Rev*. 12, 211-230. doi:10.1080/17437199.2018.1435299 **/1/**

Knoll, J., and Matthes, J. (2017). The effectiveness of celebrity endorsements: A meta-analysis. *J. Acad. Market. Sci*. 45, 55-75. doi:10.1007/s11747-016-0503-8 **/1/**

Kok, G., van den Borne, B., and Mullen, P. D. (1997). Effectiveness of health education and health promotion: Meta-analyses of effect studies and determinants of effectiveness. *Patient Educ. Couns.* 30, 19-27. doi:10.1016/S0738-3991(96)00953-6 **/1/**

Kuhberger, A. (1998). The influence of framing on risky decisions: A meta-analysis. *Organ. Behav. Hum. Decis. Process*. 75, 23-55. doi:10.1006/obhd.1998.2781 **/6/**

Kumkale, G. T., Albarracin, D., and Seignourel, P. J. (2010). The effects of source credibility in the presence or absence of prior attitudes: Implications for the design of persuasive communication campaigns. *J. Appl. Soc. Psychol*. 40*,* 1325-1356. doi:10.1111/j.1559-1816.2010.00620.x **/1/**

Kyriakaki, M. (2007). Promotion of physical health behaviours: “Framing” the persuasive message. [Doctoral dissertation]. [Colchester (UK)]: University of Essex. **/6/**

LaCroix, J. M., Snyder, L. B., Huedo-Medina, T. B., and Johnson, B. T. (2014). Effectiveness of mass media interventions for HIV prevention, 1986–2013: A meta-analysis. *J Acquir. Immune Defic. Syndr*. 66(S3), S329–S340. doi:10.1097/QAI.0000000000000230 **/1/**

Lau, R. R., Sigelman, L., Heldman, C., and Babbitt, P. (1999). The effects of negative political advertisements: A meta-analytic assessment. *Am. Political Sci. Rev*. 93, 851-875.

doi:10.2307/2586117 **/7/**

Lee, S. (2016). Examining causes and boundary conditions of the identifiable victim effect. [Doctoral dissertation]. [Buffalo (NY)]: State University of New York. ProQuest no. 10163910. **/7/**

Lee, S., and Feeley, T. H. (2017). A meta-analysis of the pique technique of compliance. *Soc. Influ.* 12, 15-28. doi:10.1080/15534510.2017.1305986 **/1/**

Lee, S., Moon, S.-I., and Feeley, T. H. (2014, November). A meta-analytical review of the legitimization of paltry favors effect. [Paper presentation]. Natl. Commun. Assoc. annual meeting, Chicago, IL. **/7/**

Lee, S., Han, S., Cheong, M., Kim, S. L., and Yun, S. (2017). How do I get my way? A meta-analytic review of research on influence tactics. *Leadersh. Q*. 28, 210-228. doi:10.1016/j.leaqua.2016.11.001 **/2/**

Leung, M. M., Agaronov, A., Grytsenko, K., and Yeh, M. C. (2012). Intervening to reduce sedentary behaviors and childhood obesity among school-age youth: A systematic review of randomized trials. *J. Obes.* Vol. 2012, article ID 685430. doi:10.1155/2012/685430 **/1/**

Li, N., and Su, L. Y.-F. (2017, August). Understanding the effects of emphasis frames on public engagement with climate change: Evidence from a meta-analysis. [Paper presentation]. Assoc. Educ. Journal. Mass Commun. annual conference, Chicago, IL. **/3/**

Lokhorst, A. M., Werner, C., Staats, H., van Dijk, E., and Gale, J. L. (2013). Commitment and behavior change: A meta-analysis and critical review of commitment-making strategies in environmental research. *Environ. Behav*. 45, 3-34. doi:10.1177/0013916511411477 **/1/**

Lull, R. B., and Bushman, B. J. (2015). Do sex and violence sell? A meta-analytic review of the effects of sexual and violent media and ad content on memory, attitudes, and buying intentions. *Psychol. Bull*. 141(5), 1022-1048. doi:10.1037/bul0000018 **/5/** (for violent-content variation; sexual-content variation included)

Lunt, D. (2016). Willing to help, but lacking discernment: The effects of victim group size on donation behaviors. [Doctoral dissertation]. [Arlington (TX)]: University of Texas at Arlington. ProQuest no. 10302039. **/4/**

Lustria, M. L. A., Noar, S. M., Cortese, J., van Stee, S., Glueckauf, R., and Lee, J. A. (2010, April). A meta-analysis of tailored behavior change interventions delivered via the web: Effects and moderators of efficacy. [Paper presentation]. Kentucky Conf. Health Commun., Lexington, KY. **/7/**

Lustria, M. L. A., Noar, S. M., Cortese, J., Van Stee, S. K., Glueckauf, R. L., and Lee, J. (2013). A meta-analysis of web-delivered tailored health behavior change interventions. *J. Health Commun*. 18, 1039-1069. doi:10.1080/10810730.2013.768727 **/1/**

Ma, L., and Zhan, M. (2016). Effects of attributed responsibility and response strategies on organizational reputation: A meta-analysis of situational crisis communication theory research. *J. Public Relat. Res.* 28, 102-119. doi:10.1080/1062726X.2016.1166367 **/2/**

Martin, M. C. (1997). Children’s understanding of the intent of advertising: A meta-analysis. *J. Public Policy Market*. 16, 205-216. doi:10.1177/074391569701600202 **/2/**

McMahon, N., Thomson, K., Kaner, E., and Bambra, C. (2019). Effects of prevention and harm reduction interventions on gambling behaviours and gambling related harm: An umbrella review. *Addict. Behav*. 90, 380-388. doi:10.1016/j.addbeh.2018.11.048 **/1/**

McRoberts, D. A., and Larson-Casselton, C. (2006). Humor in public address, health care and the workplace: Summarizing humor’s use using meta-analysis. *North Dakota Speech Theatre J.* 19, 26-33. [no doi] **/3/**

Michie, S., Abraham, C., Whittington, C., McAteer, J., and Gupta, S. (2009). Effective techniques in healthy eating and physical activity interventions: A meta-regression. *Health Psychol*. 28, 690-701. doi:10.1037/a0016136 **/1/**

Milne, S., Sheeran, P., and Orbell, S. (2000). Prediction and intervention in health-related behavior: A meta-analytic review of protection motivation theory. *J. Appl. Soc. Psychol*. 30, 106-143. doi:10.1111/j.1559-1816.2000.tb02308. **/2/**

Miller, L. S., and Gramzow, R. (2014, February). The efficacy of self-determination interventions on physical activity: A multi-level meta-analysis. [Paper presentation]. Soc. Pers. Soc. Psychol. annual conference, DK location. **/1/**

Mongeau, P. A. (1994). “Another look at fear-arousing persuasive appeals,” in *Prospects and Precautions in the Use of Mmeta-Analysis*, ed. M. Allen and R. W. Preiss (Dubuque, IA: Brown and Benchmark Eds), 75-100. **/7/**

Mongeau, P. A. (1998). “Another look at fear-arousing persuasive appeals,” in *Persuasion: Advances through Meta-Analysis*, ed. M. Allen and R. W. Preiss (New York: Hampton Press), 53-68. **/6/**

Motyka, S., Grewal, D., Puccinelli, N. M., Roggeveen, A. L., Avnet, T., Daryanto, A., de Ruyter, K., and Wetzels, M. (2014). Regulatory fit: A meta-analytic synthesis. *J. Consum. Psychol*. 24*,* 394-410. doi:10.1016/j.jcps.2013.11.004 **/1/**

Mullen, P. D., Simons-Morton, D. G., Ramirez, G., Frankowski, R. F., Green, L. W., and Mains, D. A. (1997). A meta-analysis of trials evaluating patient education and counseling for three groups of preventive health behaviors. *Patient Educ. Couns.* 32, 157-173. doi:10.1016/S0738-3991(97)00037-2 **/1/**

Nabi, R., Walter, N., Oshidary, N., Endacott, C., Lew, Z., Aune, A., and Love-Nichols, J.

(2019, May). Can emotions capture the elusive gain/loss framing effect? A meta-analysis. [Paper presentation]. Intl. Commun. Assoc. annual meeting, Washington, DC. **/6/**

Naslund, J. A. (2017). Digital technology for health promotion among individuals with serious mental illness. [Doctoral dissertation]. [Hanover (NH)]: Dartmouth College. ProQuest no. 10287765. **/7/**

Naslund, J. A., Whiteman, K. L., McHugo, G. J., Aschbrenner, K. A., Marsch, L. A., and Bartels, S. J. (2017). Lifestyle interventions for weight loss among overweight and obese adults with serious mental illness: a systematic review and meta-analysis. *Gen. Hosp. Psychiatry*, 47, 83-102. doi:10.1016/j.genhosppsych.2017.04.003 **/1/**

Nelson, J. P. (2011). Alcohol marketing, adolescent drinking and publication bias in longitudinal studies: A critical survey using meta-analysis. *J. Econ. Surv.* 25*,* 191-232. doi:10.1111/j.1467-6419.2010.00627.x **/1/**

Noar, S. M., Benac, C. N., and Harris, M. S. (2007). Does tailoring matter? Meta-analytic review of tailored print health behavior change interventions. *Psychol. Bull.* 133, 673-693. doi:10.1037/0033-2909.133.4.673 **/1/**

Noar, S. M., Black, H. G., and Pierce, L. B. (2009). Efficacy of computer technology-based HIV prevention interventions: A meta-analysis. *AIDS,* 23, 107-115. doi:10.1097/QAD.0b013e32831c5500 **/1/**

Noar, S. M., Hall, M. G,, Francis, D. B., Ribisl, K. M., Pepper, J. K., and Brewer, N. T. (2016). Pictorial cigarette pack warnings: A meta-analysis of experimental studies. *Tob. Control* 25, 341-354. doi.10.1136/tobaccocontrol-2014-051978 **/5/**

Noar, S. M., Pierce, L. B., and Black, H. G. (2010). Can computer-mediated interventions change theoretical mediators of safer sex? A meta-analysis. *Hum. Commun. Res.* 36, 261-297. doi:10.1111/j.1468-2958.2010.01376.x **/1/**

Noar, S., Rohde, J., Barker, J., Hall, M., and Brewer, N. (2019, May). How do pictorial cigarette pack warnings communicate risk? A meta-analysis. [Paper presentation]. Intl. Commun. Assoc. annual meeting, Washington, DC.

Noguchi, K., Albarracín, D., Durantini, M. R., and Glasman, L. R. (2007). Who participates in which health promotion programs? A meta-analysis of motivations underlying enrollment and retention in HIV-prevention interventions. *Psychol. Bull. 133*, 955-975. doi:10.1037/0033-2909.133.6.955 **/1/**

O’Keefe, D. J. (1987). The persuasive effects of delaying identification of high- and low-credibility communicators: A meta-analytic review. *Cent. States Speech J*. 38, 63-72. doi:10.1080/10510978709368231 **/1/**

O’Keefe, D. J. (2000). Guilt and social influence. Ann. Int. Commun. Assoc. 23(1), 67-101. doi:10.1080/23808985.2000.11678970 **/5/**

[also cited as a book chapter: In M. E. Roloff (Ed.), *Communication yearbook 23*, ed. M. E. Roloff (Thousand Oaks, CA: Sage), 67-101.]

[also cited as: *Commun. Yearbook, 23*, 67-101.]

O’Keefe, D. J., and Jensen, J. D. (2007). The relative persuasiveness of gain-framed and loss-framed messages for encouraging disease prevention behaviors: A meta-analytic review. *J. Health Commun*. 12, 623-644. doi.10.1080/10810730701615198 **/6/**

O'Keefe, D. J., and Jensen, J. D. (2008, May). The persuasiveness of gain- and loss-framed messages for encouraging disease detection: A meta-analytic review. [Paper presentation]. Intl. Commun. Assoc. annual meeting, Montreal, Quebec, Canada. **/7/**

O’Keefe, D. J., and Jensen, J. D. (2009). The relative persuasiveness of gain-framed and loss-framed messages for encouraging disease detection behaviors: A meta-analytic review. *J. Commun*. 59, 296-316. doi.10.1111/j.1460-2466.2009.01417.x **/6/**

O’Keefe, D. J., and Hale, S. L. (1998). The door-in-the-face influence strategy: A random-effects meta-analytic review. *Ann. Int. Commun. Assoc.* 21, 1-33. **/1/** https://doi:10.1080/23808985.1998.11678947

[also cited as: *Commun. Yearbook, 21*, 1-33.]

[also cited as a book chapter: in *Communication Yearbook 21,* ed. M. E. Roloff (Thousand Oaks, CA: Sage), 1-33.]

O’Keefe, D. J., and Hale, S. L. (2001). An odds-ratio-based meta-analysis of research on the door-in-the-face influence strategy. *Commun. Rep.* 14, 31-38. doi:10.1080/08934210109367734 **/1/**

O’Keefe, D. J., and Jensen, J. D. (2008). Do loss-framed persuasive messages engender greater message processing than do gain-framed messages? A meta-analytic review. *Commun. Stud.* 59, 51-67. doi:10.1080/10510970701849388 **/2/**

Orr, J. A., and King, R. J. (2015). Mobile phone SMS messages can enhance healthy behaviour: a meta-analysis of randomised controlled trials. *Health Psychol. Rev.* 9, 397-416. doi:10.1080/17437199.2015.1022847 **/1/**

Oschatz, C., and Marker, C. (2019, May). Long-term persuasive effects in narrative communication research: A meta-analysis [Paper presentation]. Intl. Commun. Assoc. annual meeting, Washington, DC. **/7/**

Oschatz, C., and Marker, C. (2020). Long-term persuasive effects in narrative communication research: A meta-analysis. *J. Commun.* 70, 473–496. doi:10.1093/joc/jqaa017 **/6/**

Ouellette, J. A., and Wood, W. (1998). Habit and intention in everyday life: The multiple processes by which past behavior predicts future behavior. *Psychol. Bull.* 124, 54-74. doi:10.1037/0033-2909.124.1.54 **/1/**

Parcell, L. M., Kwon, J., Miron, D., and Bryant, J. (2007). “An analysis of media health campaigns for children and adolescents: Do they work?” in *Mass Media Effects Research: Advances through Meta-Analysis*, ed. R. W. Preiss, B. M. Gayle, N. Burrell, M. Allen, and J. Bryant (Mahwah, NJ: Lawrence Erlbaum), 345-361. **/1/**

Parsons, J. E., Newby, K. V., and French, D. P. (2018). Do interventions containing risk messages increase risk appraisal and the subsequent vaccination intentions and uptake? A systematic review and meta‐analysis. *Br. J. Health Psychol.* 23, 1084-1106. doi:10.1111/bjhp.12340 **/1/**

Peters, G.-J. Y., Ruiter, R. A. C., and Kok, G. (2013). Threatening communication: A critical re-analysis and a revised meta-analytic test of fear appeal theory. *Health Psychol. Rev*. 7(suppl. 1), S8–S31. doi.10.1080/17437199.2012.703527 **/5/**

Phillips, R. O., Ulleberg, P., and Vaa, T. (2011). Meta-analysis of the effect of road safety campaigns on accidents. *Accid. Anal. Prev.* 43*,* 1204-1218. doi:10.1016/j.aap.2011.01.002 **/1/**

Piñon, A., and Gambara, H. (2005). A meta-analytic review of framing effect: Risky, attribute and goal framing. *Psicothema* 17, 325-331. [no doi] **/4/**

Pohl, G. M. (1991, November). Anxiety and message-induced persuasion: A meta-analytical approach. [Paper presentation]. Speech Commun. Assoc. annual convention, Atlanta, GA. **/1/**

Porath-Waller, A. J., Beasley, E., and Beirness, D. J. (2010). A meta-analytic review of school-based prevention for cannabis use. *Health Educ. Behav.* 37*,* 709-23. doi:10.1177/1090198110361315 **/1/**

Portnoy, D. B., Ferrer, R. A., Bergman, H. E., and Klein, W. M. P. (2014). Changing deliberative and affective responses to health risk: A meta-analysis. *Health Psychol. Rev.* 8, 296-318. doi:10.1080/17437199.2013.798829 **/2/**

Posavac, E. J., Kattapong, K. R., and Dew, D. E., Jr. (1999). Peer-based interventions to influence health-related behaviors and attitudes: A meta-analysis. *Psychol. Rep.* 85, 1179-1194. doi:10.2466/pr0.1999.85.3f.1179 **/1/**

Posavac, E. J., Sinacore, J. M., Brotherton, S. E., Helford, M. C., and Turpin, R. S. (1985). Increasing compliance to medical treatment regimens: A meta-analysis of program evaluation. *Eval Health Prof. 3*, 47-62. doi:10.1177/016327878500800102 **/1/**

Preiss, R. W., and Allen, M. (1994). “The persuasive effects of incentives to perform counterattitudinal advocacy,” in *Prospects and Precautions in the Use of Meta-Analysis*, ed. M. Allen and R. W. Preiss (Dubuque, IA: Brown and Benchmark), 339-361. **/7/**

Preiss, R. W., and Allen, M. (1998). “Performing counterattitudinal advocacy: The persuasive impact of incentives,” in *Persuasion: Advances through Meta-Analysis*, ed. M. Allen and R. W. Preiss (New York: Hampton Press), 231-242. **/1/**

Purmehdi, M. (2016). Two essays on customer protection initiatives. [Doctoral dissertation]. [Montreal, Canada]: HEC. ProQuest no. 10684308. **/3/**

Quinn, J. M., and Wood, W. (2004). “Forewarnings of influence appeals: Inducing resistance and acceptance,” in *Resistance and Persuasion*, ed. E. S. Knowles and J. A. Linn (Mahwah, NJ: Lawrence Erlbaum), 193-213. **/2/**

Ratcliff, C. (2017, August). Do narratives attenuate message resistance? A meta-analysis. [Paper presentation]. Assoc. Educ. Journal. Mass Commun. annual conference, Chicago, IL. **/7/**

Ratcliff, C., and Sun, Y. (2018, May). How do narratives reduce resistance? Meta-analyzing potential moderators. [Paper presentation]. Intl. Commun. Assoc. annual meeting, Prague, Czech Republic. **/2/**

Reinard, J. C. (1994). “The persuasive effects of assertive evidence,” in *Prospects and Precautions in the Use of Meta-Analysis*, ed. M. Allen and R. W. Preiss (Dubuque, IA: Brown and Benchmark), 127-157. **/7/**

Reinard, J. C. (1998). “The persuasive effects of testimonial assertion evidence,” in *Persuasion: Advances through Meta-Analysis*, ed. M. Allen and R. W. Preiss (New York: Hampton Press), 69-86. **/4/**

Reinhart, A. M. (2006). Comparing the persuasive effects of narrative versus statistical messages: A meta-analytic review. [Doctoral dissertation]. [Buffalo (NY)]: State University of New York at Buffalo. ProQuest no. 3213634. **/7/**

Reinhart, A. M., and Feeley, T. (2007, November). Comparing the persuasive effects of narrative versus statistical messages: A meta-analytic review. [Paper presentation]. Natl. Commun. Assoc. annual meeting, Chicago, IL. **/6/**

Rhodes, N., Hunt, D. M., and Radford, S. (2013, June). Fear and death: A meta-analytic review of fear appeals from a terror management perspective. [Paper presentation]. Intl. Commun. Assoc. annual meeting, London, UK. **/3/**

Rhodes, N., and Wood, W. (1992). Self-esteem and intelligence affect influenceability: The mediating role of message reception. *Psychol. Bull.* 111, 156-171. **/1/**

Rhodes, R. E., Gray, S. M., and Husband, C. (2019). Experimental manipulation of affective judgments about physical activity: A systematic review and meta-analysis of adults. *Health Psychol. Rev.* 13, 18-34. doi:10.1080/17437199.2018.1530067 **/1/**

Rodrigues, A., Sniehotta, F. F., and Araujo-Soares, V. (2013). Are interventions to promote sun-protective behaviors in recreational and tourist settings effective? A systematic review with meta-analysis and moderator analysis. *Ann. Behav. Med.* 45, 224-238. doi:10.1007/s12160-012-9444-8 **/1/**

Rogers, D., Snyder, L., and Rego, M. (2019, May). The impact of mass media-delivered family planning campaigns in low- and middle-income countries: A meta-analysis of advertising and entertainment-education format effects. [Paper presentation]. Intl. Commun. Assoc. annual meeting, Washington, DC. **/1/**

Rooney, B. L., and Murray, D. M. (1996). A meta-analysis of smoking prevention programs after adjustment for errors in the unit of analysis. *Health Educ. Q.* 23, 48-64. doi:10.1177/109019819602300104 **/1/**

Roter, D. L., Hall, J. A., Merisca, R., Nordstrom, B., Cretin, D., and Svarstad, B. (1998). Effectiveness of interventions to improve patient compliance: A meta-analysis. *Med. Care* 36, 1138-1161. https://www.jstor.org/stable/3766882 **/1/**

Rundall, T. G., and Bruvold, W. H. (1988). A meta-analysis of school-based smoking and alcohol use prevention programs. *Health Educ. Q.* 15, 317-334. doi:10.1177/109019818801500306

Sangalang, A., and Kim, M. (2017, May). When similarity is dissimilar: A meta-analysis of the dimensions of manipulated and perceived character-audience similarity on engagement and persuasion. [Paper presentation]. Intl. Commun. Assoc. annual meeting, San Diego, CA. **/4/**

Sarnoff, R., and Rundall, T. (1998). Meta-analysis of effectiveness of interventions to increase immunization rates among high-risk population groups. *Med. Care Res. Rev.* 55, 432-456. doi:10.1177/107755879805500403 **/1/**

Schermer, J. (1988). Visual media, attitude formation, and attitude change in nursing education. *Educ. Commun. Techno. J.* 36, 197-210. doi:10.1007/BF02768352 **/1/**

Schmidt, S., and Eisend, M. (2015). Advertising repetition: A meta-analysis on effective frequency in advertising. *J. Advert.* 44, 415-428. doi:10.1080/00913367.2015.1018460 **/1/**

Schumacher, A. C. (2017). Humor in public health messaging: Past, present, future. [Doctoral dissertation]. [Iowa City (IA)]: University of Iowa. ProQuest no. 10259441. **/6/**

Scott-Sheldon, L. A. J., DeMartini, K. S., Carey, K. B., and Carey, M. P. (2009). Alcohol interventions for college students improves antecedents of behavioral change: Results from a meta-analysis of 34 randomized controlled trials. *J. Soc. Clin. Psychol.* 28, 799-823. doi:10.1521/jscp.2009.28.7.799 **/1/**

Segrin, C. (1993). The effects of nonverbal behavior on outcomes of compliance gaining attempts. *Commun. Stud.* 44*,* 169-187. doi:10.1080/10510979309368393 **/1/**

Seo, K., and Kim, N. Y. (2018, May). Does adding images to texts influence persuasion? A meta-analysis of visual image effects on persuasive texts. [Paper presentation]. Intl. Commun. Assoc. annual meeting, Prague, Czech Republic. **/7/**

Seo, D.C., and Sa, J. (2010). A meta-analysis of obesity interventions among U.S. minority children. *J. Adolesc. Health* 46, 309-323. doi:10.1016/j.jadohealth.2009.11.202 **/1/**

Shaikh, S. J. (2017, May). Does “easy” really do it? Processing fluency and its effects on liking: A meta-analysis. [Paper presentation]. Intl. Commun. Assoc. annual meeting, San Diego, CA. **/1/**

Shea, S., DuMouchel, W., and Bahamonde, L. (1996). A meta-analysis of 16 randomized controlled trials to evaluate computer-based clinical reminder systems for preventive care in the ambulatory setting. *J. Am. Med. Inform. Assoc.* 3, 399-409. doi:10.1136/jamia.1996.97084513 **/1/**

Sheeran, P., Harris, P. R., and Epton, T. (2014). Does heightening risk appraisals change people’s intentions and behavior? A meta-analysis of experimental studies. *Psychol. Bull.* 140, 511-543*.* doi:10.1037/a0033065 **/1/**

Sheeran, P., and Taylor, S. (1999). Predicting intentions to use condoms: A meta-analysis and comparison of the theories of reasoned action and planned behavior. *J. Appl. Soc. Psychol.* 29, 1624-1675. doi:10.1111/j.1559-1816.1999.tb02045.x **/1/**

Shen, F. Y., and Han, J. X. (2014). Effectiveness of entertainment education in communicating health information: A systematic review. *Asian J. Commun.* 24, 605-616. doi:10.1080/01292986.2014.927895 **/3/**

Sierra, J. J., Hyman, M. R., and Heiser, R. S. (2012). Ethnic identity in advertising: A review and meta-analysis. *J. Promot. Manag.* 18, 489-513. doi:10.1080/10496491.2012.715123 **/1/**

Siopis, G., Chey, T., and Allman-Farinelli, M. (2015). A systematic review and meta-analysis of interventions for weight management using text messaging. *J. Hum. Nutr. Diet.* 28(S1), 1-15. doi:10.1111/jhn.12207 **/1/**

Six, B., and Eckes, T. (1996). Metaanalysen in der Einstellungs-Verhaltens-Forschung [Meta-analyses in attitude-behavior research.] *Z. Sozpsychol.* 27, 7-17. [no doi] **/1/**

Snyder, L. B. (2007). “Meta-analyses of mediated health campaigns,” in *Mass Media Effects Research: Advances through Meta-Analysis,* ed. R. W. Preiss, B. M. Gayle, N. Burrell, M. Allen, and J. Bryant (Mahwah, NJ: Lawrence Erlbaum), 327-344. **/1/**

Snyder, L., Diop-Sidibe, N., and Badiane, L. (2003, May). A meta-analysis of the effectiveness of family planning campaigns in less developed countries. [Paper presentation]. Intl. Commun. Assoc. annual meeting, San Diego, CA. **/1/**

Snyder, L. B., and Hamilton, M. A. (2002). “A meta-analysis of U. S. health campaign effects on behavior: Emphasize enforcement, exposure, and new information, and beware the secular trend,” in *Public Health Communication: Evidence for Behavior Change*, ed. R. C. Hornik (Mahwah, NJ: Lawrence Erlbaum), 357-383. **/1/**

Snyder, L. B., Hamilton, M. A., Mitchell, E. W., Kiwanuka-Tondo, J., Fleming-Milici, F., Proctor, D., and Freimuth, A. (1999, May). Meta-analysis of health communication campaigns to change behavior: Methodological and behavioral factors affecting success. [Paper presentation]. Intl. Commun. Assoc. annual meeting, San Francisco, CA. **/7/**

Snyder, L. B., Hamilton, M. A., Mitchell, E. W., Kiwanuka-Tondo, J., Fleming-Milici, F., and Proctor, D. (2004). A meta-analysis of the effect of mediated health communication campaigns on behavior change in the United States. *J. Health Commun. 9*(Supplement 1), 71-97. doi:10.1080/10810730490271548. **/1/**

Snyder, L., Johnston, B. T., Huedo-Medina, T., LaCroix, J. M., Smoak, N. D., and Cistulli, M. (2009, May). Effectiveness of media interventions to prevent HIV, 1986-2006: A meta-analysis. [Paper presentation]. Intl. Commun. Assoc. annual meeting, Chicago, IL. **/1/**

Sopory, P., and Dillard, J. P. (1996, May). The effects of metaphor on persuasion: A meta-analysis. [Paper presentation]. Intl. Commun. Assoc. annual meeting, Chicago, IL. **/7/**

Sopory, P., and Dillard, J. P. (2002). The persuasive effects of metaphor: A meta-analysis. *Hum. Commun. Res*. 28, 382-419. doi.10.1111/j.1468-2958.2002.tb00813.x **/6/**

Sun, W. H., Miu, H. Y. H., Wong, C. K. H., Tucker, J. D., and Wong. W. C. W. (2018). Assessing participation and effectiveness of the peer-led approach in youth sexual health education: Systematic review and meta-analysis in more developed countries. *J. Sex Res.* 55, 31-44. doi:10.1080/00224499.2016.1247779 **/1/**

Sutton, S. R. (1982). “Fear-arousing communications: A critical examination of theory and research,” in *Social Psychology and Behavioral Medicine*, ed. J. R. Eiser (New York: Wiley), 303-337. **/6/**

Tannenbaum, M. B., Hepler, J., Zimmerman, R. S., and Albarracin, D. (2014, February). A meta-analysis of information exposure strategies and scare tactics: Selective exposure moderates the efficacy of fear appeals. [Paper presentation]. Soc. Pers. Soc. Psychol., annual conference, DK location. **/7/**

Tannenbaum, M. (2015). Appealing to fear: A meta-analysis of fear appeal effectiveness and theories. [Doctoral dissertation]. [Urbana Champaign (IL)]: University of Illinois at Urbana Champaign. **/7/**

Tannenbaum, M. B., Hepler, J., Zimmerman, R. S., Saul, L., Jacobs, S., Wilson, K., and Albarracín, D. (2015). Appealing to fear: A meta-analysis of fear appeal effectiveness and theories. *Psychol. Bull*. 141, 1178-1204. doi:10.1037/a0039729 **/3/**

Tanner-Smith, E. E., and Risser, M. D. (2016). A meta-analysis of brief alcohol interventions for adolescents and young adults: Variability in effects across alcohol measures.

*Am. J. Drug Alcohol Abuse* 42*,* 140-151. doi:10.3109/00952990.2015.1136638 **/1/**

Tobler, N. S. (1986). Meta-analysis of 143 adolescent drug prevention programs: Quantitative outcome results of program participants compared to a control or comparison group. *J. Drug Issues* 16, 537-567. doi:10.1177/002204268601600405 **/1/**

Townson, C. (2019). The anchoring effect: A meta-analysis. [Doctoral dissertation]. [East Lansing (MI)]: Michigan State University. ProQuest no. 22615195. **/1/**

Trappey, C. (1996). A meta-analysis of consumer choice and subliminal advertising. *Psychol. Mark.* 13, 517-530. doi:10.1002/(SICI)1520-6793(199608)13:5%3C517::AID-MAR5%3E3.0.CO;2-C **/1/**

Tukachinsky, R., and Tokunaga, R. S. (2013). The effects of engagement with entertainment. *Ann. Int. Commun. Assoc.* 37, 287-322. doi:10.1080/23808985.2013.11679153 **/1/**

[also cited as a book chapter: in *Communication Yearbook 37,* ed. E. L. Cohen (New York: Routledge), 287-321.]

van der Lee, A., and van den Putte, B. (2001, June). From fame to fortune: A meta-analytic review of celebrity endorsements in persuasive communication. [Paper presentation]. Eur. Assoc. Consum. Res., annual meeting, Berlin, Germany. **/1/**

van Grieken, A., Ezendam, N.P., Paulis, W.D., van der Wouden, J.C., and Raat, H. (2012). Primary prevention of overweight in children and adolescents: A meta-analysis of the effectiveness of interventions aiming to decrease sedentary behaviour. *Int. J. Behav. Nutr. Phys. Act.* 9(61). doi:10.1186/1479-5868-9-61 **/1/**

van Laer, T., de Ruyter, K., Visconti, L. M., and Wetzels, M. (2014). The extended transportation-imagery model: A meta-analysis of the antecedents and consequences of consumers’ narrative transportation. *J. Consum. Res.* 40, 797-817. doi:10.1086/673383 **/1/**

Van Stee, S. K. (2018). Meta-analysis of the persuasive effects of metaphorical vs. literal messages. *Commun. Stud*. 69, 545-566. doi.10.1080/10510974.2018.1457553 **/6/**

Walter, N., Cody, M. J., Xu, L. Z., and Murphy, S. T. (2017, May). Meta-analysis of humor effects on persuasion. [Paper presentation]. Intl. Commun. Assoc. annual meeting, San Diego, CA. **/7/**

Walter, N., Tukachinsky, R., Nabi, R. L., and Pelled, A. (2018, November). Meta-analysis of anger and persuasion: An empirical integration of three models. [Paper presentation]. Natl. Commun. Assoc. annual meeting, Salt Lake City, UT. **/7/**

Walter, N., Tukachinsky, R., Pelled, A., and Nabi, R. (2019). Meta-analysis of anger and persuasion: An empirical integration of four models. *J. Comm.* 69, 73-93. doi:10.1093/joc/jqy054 **/1/**

Wanyonyi, K. L., Themessl-Huber, M., Humphris, G., and Freeman, R. (2011). A systematic review and meta-analysis of face-to-face communication of tailored health messages: Implications for practice. *Patient Educ. Couns*. 85*,* 348-355. doi:10.1016/j.pec.2011.02.006 **/1/**

Webb, T. L., Joseph, J., Yardley, L., and Michie, S. (2010). Using the Internet to promote health behavior change: A systematic review and meta-analysis of the impact of theoretical basis, use of behavior change techniques, and mode of delivery on efficacy. *J. Medical Internet Res.* 12*,* e4. doi:10.2196/jmir.1376 **/1/**

Werb, D., Mills, E. J., DeBeck, K., Kerr, T., Montaner, J. S. G., and Wood, E. (2011) The effectiveness of anti-illicit-drug public-service announcements: A systematic review and meta-analysis. *J. Epidemiology Community Health* 65*,* 834-840. doi:10.1136/jech.2010.125195 **/1/**

Wildeboer, G., Kelders, S. M., and van Gemert-Pijnen, J. E. (2016). The relationship between persuasive technology principles, adherence and effect of web-based interventions for mental health: A meta-analysis. *Int. J. Med. Inform.* 96, 71-85*.* doi:10.1016/j.ijmedinf.2016.04.005 **/1/**

Williams, A. L., Grogan, S., Clark-Carter, D., and Buckley, E. (2013). Appearance-based interventions to reduce ultraviolet exposure and/or increase sun protection intentions and behaviours: A systematic review and meta-analyses. *Br. J. Health Psychol.* 18*,* 182–217. doi:10.1111/j.2044-8287.2012.02089.x **/3/**

Wilson, E. J., and Sherrell, D. L. (1993). Source effects in communication and persuasion research: A meta-analysis of effect size. *J. Acad. Mark. Sci.* 21, 101-112. doi:10.1007/BF02894421 **/1/**

Wilson, K., Senay, I., Durantini, M., Sanchez, F., Hennessy, M., Spring, B., and Albarracin, D. (2014, February). Do more lifestyle recommendations equal healthier outcomes? A meta-analysis of theoretical assumptions underlying the effectiveness of interventions promoting multiple-behavior change. [Paper presentation]. Soc. Pers. Soc. Psychol. conference, Austin, TX. **/1/**

Wirtz, J., Sparks, J., and Lyons, K. (2012, August). Sex sells? A meta-analysis of the effect of sexual content in advertisements on persuasive outcomes. [Paper presentation]. Assoc. Educ. Journal. Mass Commun. annual meeting, Chicago, IL. **/7/**

Wirtz, J. G., Sparks, J. V., and Zimbres, T. M. (2018). The effect of exposure to sexual appeals in advertisements on memory, attitude, and purchase intention: A meta-analytic review. *Int. J. Advert.* 37, 168-198. doi.10.1080/02650487.2017.1334996 **/4/**

Witte, K., and Allen, M. (1996, November). When do scare tactics work? A meta-analysis of fear appeals. [Paper presentation]. Speech Commun. Assoc. annual convention, San Diego, CA. **/7/**

Wood, W., and Quinn, J. M. (2002, April). Forewarned and forearmed? A meta-analytic synthesis of forewarning experiments. [Paper presentation]. University Arkansas Symp. Resistance and Persuasion, Fayetteville, AR. **/7/**

Wood, W., and Quinn, J. M. (2003). Forewarned and forearmed? Two meta-analytic syntheses of forewarnings of influence appeals. *Psychol. Bull.* 129, 119-138. doi:10.1037/0033-2909.129.1.119 **/2/**

Wood, W., Lundgren, S., Ouellette, J. A., Busceme, S., and Blackstone, T. (1994). Minority influence: A meta-analytic review of social influence processes. *Psychol. Bull.* 115, 323-345. doi:10.1037/0033-2909.115.3.323 **/1/**

Xu, A. Y. (2018, November). Effectiveness of web-based interventions for smoking cessation: A meta-analysis of randomized controlled trials. [Paper presentation]. Natl. Commun. Assoc. annual meeting, Salt Lake City, UT. **/1/**

Xu, Z. (2017, May). A meta-analysis of the effectiveness of guilt on health-related attitudes and intentions. [Paper presentation]. Intl. Commun. Assoc. annual meeting, San Diego, CA. **/7/**

Xu, Z., and Guo, H. (2018). A meta-analysis of the effectiveness of guilt on health-related attitudes and intentions. *Health Commun.* 33, 519-525. doi:10.1080/10410236.2017.1278633 **/1/**

Xu, Z., Huang, F., Kösters, M., Staiger, T., Becker, T., Thornicroft, G., and Rüsch, N. (2018). Effectiveness of interventions to promote help-seeking for mental health problems: Systematic review and meta-analysis. *Psychol. Med.* 48, 2658-2667. doi:10.1017/S0033291718001265 **/1/**

Yang, F., and Shen, F. (2015, May). Persuasion effects of Web interactivity: A meta-analysis. [Paper presentation]. Intl. Commun. Assoc. annual meeting, San Juan, Puerto Rico. **/1/**

Yang, Q. (2016, November). Are social networking sites making health behavior change interventions more effective? A meta-analytic review. [Paper presentation]. Natl. Commun. Assoc. annual meeting, Philadelphia, PA. **/7/**

Yang, Q. (2017). Are social networking sites making health behavior change interventions more effective? A meta-analytic review. *J. Health Commun.* 22, 223-233. doi:10.1080/10810730.2016.1271065 **/1/**

Yang, F., and Huang, X. (2019, May). The V-shape model of the persuasiveness of interactivity: A meta-analysis. [Paper presentation]. Intl. Commun. Assoc. annual meeting, Washington, DC. **/3/**

Yang, Q., and Millette, D. M. (2015, November). Does interactivity help? A meta-analytic review of web-based interactive health interventions. [Paper presentation]. Natl. Commun. Assoc. annual meeting, Las Vegas, NV. **/1/**

Yang, Q., and Van Stee, S. (2018, November). The comparative effectiveness of mHealth interventions in improving health outcomes: A meta-analytic review. [Paper presentation]. Natl. Commun. Assoc. annual meeting, Salt Lake City, UT. **/1/**

Yang, Q., and Van Stee, S. K. (2018, May). *U*nderstanding the effectiveness of mHealth interventions: A meta-analysis of health behavior change interventions using mobile phones. [Paper presentation]. Intl. Commun. Assoc. annual meeting, Czech Republic. **/1/**

Yang, Q., and Van Stee, S. K. (2019). The comparative effectiveness of mobile phone interventions in improving health outcomes: Meta-analytic review. *JMIR Mhealth Uhealth*, 7, e11244. doi:10.2196/11244 **/1/**

Yang, Z. J., Aloe, A. M., and Feeley, T. H. (2014). Risk information seeking and processing model: A meta-analysis. *J. Commun.* 64, 20–41. doi:10.1111/jcom.12071 **/1/**

Yin, L., Wang, N., Vermund, S. H., Shepherd, B. E., Ruan, Y., Shao, Y., and Qian, H. Z. (2014). Sexual risk reduction for HIV-infected persons: A meta-analytic review of “positive prevention” randomized clinical trials. *PLoS One 9*, e107652. doi:10.1371/journal.pone.0107652 **/1/**

Yuen, R. K. (2004). The effectiveness of culturally tailored interventions: A meta-analytic review. [Doctoral dissertation]. [Chicago (IL)]: Loyola University. ProQuest no. 3143928. **/1/**

Zebregs, S., van den Putte, B., Neijens, P., and de Graaf, A. (2015). The differential impact of statistical and narrative evidence on beliefs, attitude, and intention: A meta-analysis. *Health Commun.* 30, 282-289. doi.10.1080/10410236.2013.842528 **/6/**

**APPENDIX 2**

DETAILED DESCRIPTION OF INCLUDED META-ANALYSES

**Appeal framing (gain vs. loss)**. This message variable contrasts gain-framed messages, which emphasize the advantages of compliance with the recommended action, and loss-framed messages, which emphasize the disadvantages of not complying with the recommended action. The dataset of O’Keefe and Jensen (2006) yielded 165 persuasion ESs, a larger number of cases than available from other reviews (Akl et al., 2011; Freling, 2017; Gallagher and Updegraff, 2012; Kuhberger, 1998; Kyriakaki, 2007; O’Keefe and Jensen, 2007, 2009; Piñon and Gambara, 2005). Eleven moderating conditions were analyzed, drawn from three moderators; fifteen levels arising from combinations of two moderators were also analyzed. Positive ESs indicated a persuasive advantage for gain-framed appeals.

**Argument explicitness (explicit vs. implicit).** This message variable reflects variation in how explicitly the message spells out the support for its overall conclusion, such as variation in whether the premises or conclusions of supporting arguments were stated explicitly; the contrast thus is between relatively explicit and relatively implicit arguments. The dataset of O’Keefe (1998) provided 18 persuasion ESs. Positive ESs indicated a persuasive advantage for explicit arguments.

**Argument strength (strong vs. weak).** Argument strength is not an entirely well-defined property. Whereas some researchers have manipulated argument strength using independent criteria, the majority have identified strong and weak arguments through pretests. Most commonly, participants have been asked to list their thoughts while reflecting upon the arguments; arguments were considered strong (or weak) if they mainly evoked favorable (or unfavorable) thoughts. But when argument strength is manipulated in this way, it is not an illuminating message property; any main effect of argument strength could only be taken to show that messages that are relatively more effective in pretesting are also relatively more effective subsequently. (For additional discussion of argument strength manipulations, see Hoeken, Hornikx, and Linders, 2020.)

But Carpenter’s (2015) meta-analysis usefully distinguished studies in which argument strength was realized through such pretesting and studies in which it was manipulated by varying message properties.^3^ Only the latter cases were included here, yielding 13 attitude ESs (collapsed across central and peripheral processing conditions). Two moderating conditions were analyzed, drawn from one moderator. Positive ESs indicated a persuasive advantage for strong arguments.

**“But you are free” (included vs. omitted)**. This message variable refers to the adding of an expression that emphasizes a person’s right to say “no” to a request. The expression most frequently used is “But you are free to accept or to refuse.” The contrast is thus between a request followed by such an expression and a request without it. The meta-analysis of Carpenter (2013) was based on 42 compliance ESs. Four moderating conditions were analyzed, drawn from two moderators; two levels arising from combinations of two moderators were also analyzed. Positive ESs indicated a higher compliance rate for requests including an expression about the person’s right to refuse.

**Conclusion (included vs. omitted)**. This message variable reflects variation in the explicitness of the message’s overall conclusion; the contrast thus is between messages in which an overtly-stated conclusion is included (explicit conclusion) and messages in which the conclusion is left unstated (implicit conclusion). The meta-analysis of O’Keefe (2002) was based on 17 persuasion ESs, a larger number of cases than was that of Cruz (1998). Positive ESs indicated a persuasive advantage for messages with explicit conclusions.

**Cultural tailoring (deep-tailored vs. not-tailored)**. This message variable reflects variation in the degree to which the message is adapted to deep aspects of the audience’s cultural background, such as cultural values and religious beliefs; the contrast thus is between deep-tailored messages and not-tailored messages. The meta-analysis of Hornikx and O’Keefe (2009) provided 67 persuasion ESs, a larger number of cases than did that of Huang and Shen (2016). Five moderating conditions were analyzed, drawn from two moderators; three levels arising from combinations of two moderators were also analyzed. Positive ESs indicated a persuasive advantage for deep-tailored messages.

**Depicted response efficacy (high vs. low)**. This threat appeal-related message variable reflects variation in the message’s depiction of the efficaciousness (effectiveness or efficacy) of the recommended course of action; the contrast thus is between messages that depict the recommended action as relatively more effective (high response efficacy) and messages that depict the recommended action as relatively less effective (low response efficacy). The meta-analysis of Witte and Allen (2000) was based on 24 intention ESs, a larger number of cases than available in other meta-analyses (e.g., de Hoog, Stroebe, and de Wit, 2007; Floyd, Prentice-Dunn, and Rogers, 2000; Peters, Ruiter, and Kok, 2013). Positive ESs indicated a persuasive advantage for messages with high depicted response efficacy.

**Depicted self-efficacy (high vs. low)**. This threat appeal-related message variable reflects variation in the message’s depiction of the message recipient’s ability to adopt or engage in the advocated action; the contrast thus is between messages that depict the action as one that is relatively easy to adopt (high self-efficacy) and messages that depict the action as one that is relatively difficult to adopt (low self-efficacy). The meta-analysis of Witte and Allen (2000) provided 21 intention ESs, a larger number of cases than was available from Floyd et al. (2000) or Peters et al. (2013). Positive ESs indicated a persuasive advantage for messages with high depicted self-efficacy.

**Depicted threat severity (high vs. low)**. This threat appeal-related message variable reflects variation in the message’s depiction of the severity of a potential threat; the contrast is thus between a message suggesting that the threat is relatively more severe (high threat severity) and messages suggesting that the threat is relatively less severe (low threat severity). The meta-analysis of de Hoog et al. (2007) was based on 55 intention ESs, a larger number of cases than was provided by Floyd et al. (2000), Peters et al. (2013), or Witte and Allen (2000). Two moderating conditions were analyzed, drawn from one moderator. Positive ESs indicated a persuasive advantage for high depicted threat severity.

**Depicted threat vulnerability (high vs. low)**. This threat appeal-related message variable reflects variation in the message’s depiction of the vulnerability (susceptibility) of the message recipient to the potential threat; the contrast thus is between messages that depict the receiver as relatively more vulnerable to the threat (high threat vulnerability) and messages that depict the receiver as relatively less vulnerable to the threat (low threat vulnerability). The meta-analysis of de Hoog et al. (2007) was based on 32 intention ESs, a larger number of cases than was provided by Floyd et al. (2000), Peters et al. (2013), or Witte and Allen (2000). One moderating condition was analyzed. Positive ESs indicated a persuasive advantage for messages with high depicted threat vulnerability.

**Disrupt-then-reframe (vs. reframe-only).** This request variation examines the effects on compliance of using confusing or strange language (“disrupt”) prior to presenting a request accompanied by a reason to comply (“reframe”); the contrast thus is between a disrupt-then-reframe request and a reframe-only request. The meta-analysis of Carpenter and Boster (2009) was based on 14 compliance ESs. One moderating condition was analyzed. Positive ESs indicated a persuasive advantage for disrupt-then-reframe messages.

**Evidence amount (high vs. low).** This message variable reflects variation in the amount evidence offered to support the message’s claims; the contrast thus is between messages with higher or lower amounts of evidence. The meta-analysis of Stiff (1985, 1986) was based on 31 attitude ESs. Positive ESs indicated a persuasive advantage for high-evidence messages.

**Evidence type (narrative vs. statistical).** This message variable reflects variation in the kind of evidence offered to support the message’s claim; the contrast is specifically between narrative (e.g., an individual example) and statistical evidence. The meta-analysis of Allen and Preiss (1997) was based on 16 persuasion ESs, a larger number of cases than were those of Reinhart (2006), Reinhart and Feeley (2007), and Zebregs, van den Putte, Neijens, and de Graaf (2015). Positive ESs indicated a persuasive advantage for statistical evidence.

**Humor (humorous vs. non-humorous).**This message variable contrasts humorous and non-humorous messages. The meta-analysis of Walter, Cody, Xu, and Murphy (2018) provided 58 attitude ESs, a larger number of cases than was available from Eisend (2009) or Schumacher (2017). Fourteen moderating conditions were analyzed, drawn from seven moderators; twenty-six levels arising from combinations of two moderators were also analyzed. Positive ESs indicated a persuasive advantage for humorous messages.

**Information-source identification (included vs. omitted).** This message variable contrasted messages that cited (explicitly identified) the sources of the message’s information and messages that presented the same information without identifying the information sources. The dataset of O’Keefe (1998) provided 13 persuasion ESs. Positive ESs indicated a persuasive advantage for identifying information sources.

**Language intensity (high vs. low).** This message variable reflects variation in the degree of affect expressed linguistically, that is, the degree to which the message’s language is affectively charged; the contrast thus is between messages high or low in language intensity. The meta-analysis of Hamilton and Hunter (1998) was based on 15 attitude ESs. Positive ESs indicated a persuasive advantage for high-intensity messages.

**Legitimizing paltry contributions (included vs. omitted)**. This message variable concerns a donation-request strategy that explicitly legitimizes making a small contribution, as compared to a control-condition request without such legitimization. The meta-analysis of Bolkan and Rains (2017) was based on 34 compliance ESs, a larger number than in the reviews of Andrews, Carpenter, Shaw, and Boster (2008) and Lee, Moon, and Feeley (2016). Positive ESs indicated greater compliance in the legitimizing-paltry-contributions condition.

**Metaphorical (vs. non-metaphorical)**. This message variable concerns the use of metaphorical messages as compared to literal (non-metaphorical) counterparts. The meta-analysis of Brugman, Burgers, and Vis (2019) was based on 91 persuasion ESs, a larger number of cases than were those of Sopory and Dillard (2002) or Van Stee (2018). Eight moderating conditions were analyzed, drawn from two moderators; eighteen levels arising from combinations of two moderators were also analyzed. Positive ESs indicated a persuasive advantage for metaphorical messages.

**Narrative (narrative vs. non-narrative).** This message variable concerns the use of messages in a narrative form (e.g., stories, anecdotes, testimonials) as compared to a non-narrative message. The meta-analysis by Shen, Sheer, and Li (2015) reported 34 persuasion ESs. Six moderating conditions were analyzed, drawn from three moderators; eight levels arising from combinations of two moderators were also analyzed. Positive ESs indicated a persuasive advantage for narrative messages.

**Political advertising tone (negative vs. positive).** This message variable contrasts political messages varying in evaluative tone; the contrast thus is between negative and positive political advertising. The meta-analysis of Lau, Sigelman, and Rovner (2007) was based on 27 intention ESs, a larger number of cases than was that of Hale (1998). Positive ESs indicated a persuasive advantage for negative advertising.

**Recommendation specificity (specific vs. general).**This message variable contrasts messages on the basis of the specificity of the description of the recommended action; the contrast thus is between messages that provide only a general description of the recommended action and messages that provide a more detailed recommendation. The meta-analysis of O’Keefe (2002) was based on 18 persuasion ESs. Positive ESs indicated a persuasive advantage for messages with more specific recommendations.

**Rhetorical questions (vs. statements).** This message variable concerns the use of questions to convey assertions (by virtue of the answer being apparent; e.g., “Do you want to be healthy?”). The contrast is thus between messages containing one or more rhetorical questions and messages containing the corresponding assertion(s). The meta-analysis by Gayle, Preiss, and Allen (1998) was based on 18 persuasion ESs. Positive ESs indicated a persuasive advantage for rhetorical questions.

**Sexual content (sexual vs. non-sexual).** This message variable contrasts messages especially advertisements, varying in the presence of explicit sexual content; the contrast thus is between messages with such content and messages that lack it. The meta-analysis of Lull and Bushman (2015) provided 11 attitude ESs, a larger number of cases than were available from those of Edison (2008) and Wirtz, Sparks, and Zimbres (2018). Positive ESs indicated a persuasive advantage for messages with sexual content.

**Sidedness (one-sided vs. two-sided).** This message variable contrasts one-sided messages, which present only supporting arguments, and two-sided messages, which both present supporting arguments and discuss opposing arguments. The meta-analysis of O’Keefe (1999) was based on 107 persuasion ESs, a larger number of cases than in meta-analyses by Allen (1991) and Eisend (2006, 2007). Ten moderating conditions were analyzed, drawn from six moderators; twenty-eight levels arising from combinations of two moderators were also analyzed. Positive ESs indicated a persuasive advantage for two-sided messages.

**Speaking rate (faster vs. slower).** This message variable concerns different speaking rates; the contrast is thus between a message presented at a relatively faster rate compared to a relatively slower rate. The meta-analysis by Preiss, Allen, Gayle, and Kim (2014) was based on 44 persuasion ESs. Positive ESs indicated a persuasive advantage for faster speaking rates.

**That’s not all (included vs. omitted).** This message variable reflects variation in whether, after an initial offer, a requester improves the deal in some way; the contrast is between a that’s-not-all condition and a control condition in which the requester’s offer is identical to the final offer in the that’s-not-all condition. Lee, Moon, and Feeley’s (2019) meta-analysis was based on 14 ESs. Two moderating conditions were analyzed, drawn from two moderators. Positive ESs indicated a persuasive advantage for the that’s-not-all condition.

**Threat appeal strength (strong vs. weak)**. Threat appeal strength is not an entirely well-defined property, with the consequence that a number of different message variations appear to have been collected under this or related labels (e.g., “high-fear” vs. “low-fear” appeals). Commonly, however, messages seem to have varied in the explicitness and vividness of the depictions of the threatened consequences; the contrast is thus between strong (relatively more explicit and vivid) and weak (relatively less explicit and vivid) fear appeals. The meta-analysis of White and Albarracín (2018) was based on 48 behavior ESs, a larger number of cases than were available from other meta-analyses (e.g., Boster and Mongeau, 1984; Mongeau, 1998; Sutton, 1982; Witte and Allen, 2000). Positive ESs indicated a persuasive advantage for strong threat appeals.

**Victim description (identifiable vs. non-identifiable).** This charity-appeal variable contrasts a message that mentions an identifiable individual victim and a message that mentions only an anonymous victim(s) or statistical summaries. The meta-analysis of Lee and Feeley (2016) was based on 41 persuasion ES, a larger number of cases than was available from the related meta-analyses of Freling (2017) and Lunt (2016). Nineteen moderating conditions were analyzed, drawn from 11 moderators; one hundred seventeen levels arising from combinations of two moderators were also analyzed. Positive ESs indicated a persuasive advantage for messages with an identifiable victim.

**Visual material (text-plus-visual vs. text-only).** This message variable contrasts text-only messages and text-plus-visual-material messages. The meta-analysis of Seo and Kim (2018; see also Seo, 2020) was based on 20 persuasion ESs, a larger number of cases than in Noar, Hall, Francis, Ribisl, Pepper, and Brewer (2016) or in Noar, Rohde, Barker, Hall, and Brewer (2019). Two moderating conditions were analyzed, drawn from two moderators. Positive ESs indicated a persuasive advantage for text-plus-visual messages.

**Vividness (vivid vs. pallid).** This message variable reflects variation in the extent to which the message incorporates features such as pictures, concrete or affectively charged language, audio or video recordings; the contrast thus is between messages containing such features (vivid) and messages lacking them (pallid). The meta-analysis by Blondé and Girandola (2016) was based on 37 attitude ESs, a larger number than was available from Guadagno et al. (2013). Six moderating conditions were analyzed, drawn from four moderators; five levels arising from combinations of two moderators were also analyzed. Positive ESs indicated a persuasive advantage for vivid messages.

**References**

Akl, E. A., Oxman, A. D., Herrin, J., Vist, G. E., Terrenato, I., Sperati, F., Costiniuk, C., Blank, D., and Schünemann, H. (2011). Framing of health information messages. *Cochrane Database Syst. Rev.* 2011 Issue 12 (article no. CD006777). doi.10.1002/14651858.CD006777.pub2

Allen, M. (1991). Meta-analysis comparing the persuasiveness of one-sided and two-sided messages. *West. J. Speech Commun.* 55, 390-404. doi.10.1080/10570319109374395

Allen, M., and Preiss, R. W. (1997). Comparing the persuasiveness of narrative and statistical evidence using meta-analysis.  *Commun. Res. Rep.* 14(2), 125-131. doi:10.1080/08824099709388654

Andrews, K. R., Carpenter, C. J., Shaw, A. S., and Boster, F. J. (2008). The legitimization of paltry favors effect: A review and meta-analysis. *Commun. Rep.* 21, 59-69. doi.10.1080/08934210802305028

Blondé, J., and Girandola, F. (2016). Revealing the elusive effects of vividness: A meta-analysis of empirical evidences assessing the effect of vividness on persuasion. *Soc. Influ.* 11(2), 111-129. doi:10.1080/15534510.2016.1157096

Bolkan, S., and Rains, S. A. (2017). The legitimization of paltry contributions as a compliance-gaining technique: A meta-analysis testing three explanations. *Commun. Res.* 44(7), 976–996. doi:10.1177/0093650215602308

Boster, F. J., and Mongeau, P. (1984). Fear-arousing persuasive messages. *Ann. Int. Commun. Assoc. 8,* 330-375. doi.10.1080/23808985.1984.11678581

Brugman, B. C., Burgers, C., and Vis, B. (2019). Metaphorical framing in political discourse through words vs. concepts: A meta-analysis. *Lang. Cogn.* 11(1), 41-65. doi:10.1017/langcog.2019.5

Carpenter, C. J. (2013). A meta-analysis of the effectiveness of the “but you are free” compliance-gaining technique. *Commun. Stud.* 64(1), 6-17. doi:10.1080/10510974.2012.727941

Carpenter, C. J. (2015). A meta-analysis of the ELM’s argument quality × processing type predictions. *Hum. Commun. Res.* 41(4), 501-534. doi:10.1111/hcre.12054

Carpenter, C. J., and Boster, F. J. (2009). A meta-analysis of the effectiveness of the disrupt-then-reframe compliance gaining technique. *Commun. Rep.* 22(2), 55-62. doi:10.1080/08934210903092590

Cruz, M. G. (1998). Explicit and implicit conclusions in persuasive messages. In *Persuasion: Advances through Meta-Analysis*, ed. M. Allen and R. W. Preiss (New York: Hampton Press), 217-230.

De Hoog, N., Stroebe, W., and de Wit, J. (2007). The impact of vulnerability to and severity of a health risk on processing and acceptance of fear-arousing communications: A meta-analysis. *Rev. Gen. Psychol.* 11(3), 258-285. doi:10.1037/1089-2680.11.3.258

Edison, A. S. (2008). Does sex really sell? Research on sex in advertising: A meta-analysis. [Doctoral dissertation]. [Tuscaloosa (AL)]: University of Alabama. ProQuest no. 3385368.

Eisend, M. (2006). Two-sided advertising: A meta-analysis. *Int. J. Res. Mark.* 23(2), 187-198. doi:10.1016/j.ijresmar.2005.11.001

Eisend, M. (2007). Understanding two-sided persuasion: An empirical assessment of theoretical approaches. *Psychol. Mark.* 24, 615-640. doi.10.1002/mar.20176

Eisend, M. (2009). A meta-analysis of humor in advertising. *J Acad. Mark. Sci.* 37, 191-203. doi:10.1007/s11747-008-0096-y

Floyd, D. L., Prentice-Dunn, S., and Rogers, R. W. (2000). A meta-analysis of research on protection motivation theory. *J. Appl. Soc. Psychol.* 30(2), 407-429. doi:10.1111/j.1559-1816.2000.tb02323.x

Freling, R. E. (2017). Understanding influences and biases in decision making: Three meta-analyses on information processing. [Doctoral dissertation]. [Dallas (TX)]: University of Texas at Dallas. ProQuest no. 10759978.

Gallagher, K. M., and Updegraff, J. A. (2012). Health message framing effects on attitudes, intentions, and behavior: A meta-analytic review. *Ann. Behav. Med.* 43, 101-116. doi.10.1007/s12160-011-9308-7

Gayle, B. M., Preiss, R. W., and Allen, M. (1998). Another look at the use of rhetorical questions. In *Persuasion: Advances through Meta-Analysis*, ed. M. Allen and R. W. Preiss (New York: Hampton Press), 189-201.

Guadagno, R. E., Okdie, B. M., Sagarin, B. J., DeCoster, J., and Rhoads, K. V. L. (2013). *Understanding the elusive vividness effect: A meta-analysis on vividness and persuasion*. Unpublished manuscript.

Hale, S. L. (1998, April). Attack messages and their effects on judgments of political candidates: A random-effects meta-analytic review. [Paper presentation]. Midwest Polit. Sci. Assoc. annual meeting, Chicago, IL.

Hamilton, M. A., and Hunter, J. E. (1998). The effect of language intensity on receiver evaluations of message, source, and topic. In *Persuasion: Advances through Meta-Analysis*, ed. M. Allen and R. W. Preiss (New York: Hampton Press), 99-138.

Hoeken, H., Hornikx, J., and Linders, Y. (2020). The importance and use of normative criteria to manipulate argument quality. *J Advert.* 49(2), 195-201. doi.10.1080/00913367.2019.1663317

Hornikx, J., and O’Keefe, D. J. (2009). Adapting consumer advertising appeals to cultural values: A meta-analytic review of effects on persuasiveness and ad liking. *Ann. Int. Commun. Assoc.* 33(1), 39-71. doi:10.1080/23808985.2009.11679084

Huang, Y., and Shen, F. (2016). Effects of cultural tailoring on persuasion in cancer communication: A meta-analysis. *J. Commun.* 66, 694-715. doi.10.1111/jcom.12243

Kuhberger, A. (1998). The influence of framing on risky decisions: A meta-analysis. *Organ. Behav. Hum. Decis. Process.* 75, 23-55.

Kyriakaki, M. (2007). Promotion of physical health behaviours: “Framing” the persuasive message.  [Doctoral dissertation]. [Colchester (UK)]: University of Essex.

Lau, R. R., Sigelman, L., and Rovner, I. B. (2007). The effects of negative political campaigns: A meta-analytic reassessment. *J. Polit.* 69(4), 1176-1209. doi:10.1111/j.1468-2508.2007.00618.x

Lee, S., and Feeley, T. H. (2016). The identifiable victim effect: A meta-analytic review. *Soc. Influ.* 11(3), 199-215. doi:10.1080/15534510.2016.1216891

Lee, S., Moon, S.-I., and Feeley, T. H. (2016). A meta-analytic review of the legitimization of paltry favors compliance strategy. *Psychol. Rep.* 118, 748-771. doi.10.1177/0033294116647690

Lee, S., Moon, S.-I., and Feeley, T. H. (2019). The 'that’s-not-all' compliance-gaining technique: When does it work? *Soc. Influ.* 14(2), 25-39. doi:10.1080/15534510.2019.1634146

Lull, R. B., and Bushman, B. J. (2015). Do sex and violence sell? A meta-analytic review of the effects of sexual and violent media and ad content on memory, attitudes, and buying intentions. *Psychol. Bull.* 141(5), 1022-1048. doi:10.1037/bul0000018

Lunt, D. (2016). Willing to help, but lacking discernment: The effects of victim group size on donation behaviors. [Doctoral dissertation]. [Arlington (TX)]: University of Texas at Arlington. ProQuest no. 10302039.

Mongeau, P. A. (1998). Another look at fear-arousing persuasive appeals. In *Persuasion: Advances through Meta-Analysis*, ed. M. Allen and R. W. Preiss (New York: Hampton Press), 53-68.

Noar, S. M., Hall, M. G,, Francis, D. B., Ribisl, K. M., Pepper, J. K., and Brewer, N. T. (2016). Pictorial cigarette pack warnings: A meta-analysis of experimental studies. *Tob. Control* 25, 341-354. doi.10.1136/tobaccocontrol-2014-051978

Noar, S., Rohde, J., Barker, J., Hall, M., and Brewer, N. (2019, May). How do pictorial cigarette pack warnings communicate risk? A meta-analysis. [Paper presentation]. Int. Commun. Assoc. annual meeting, Washington, DC.

O’Keefe, D. J. (1998). Justification explicitness and persuasive effect: A meta-analytic review of the effects of varying support articulation in persuasive messages. *Argumen. Advocacy* 35(2), 61-75. doi:10.1080/00028533.1998.11951621

O’Keefe, D. J. (1999). How to handle opposing arguments in persuasive messages: A meta-analytic review of the effects of one-sided and two-sided messages. *Ann. Int. Commun. Assoc.* 22(1), 209-249. doi:10.1080/23808985.1999.11678963

O’Keefe, D. J. (2002). The persuasive effects of variation in standpoint articulation. In *Advances in Pragma-Dialectics*, ed. F. H. van Eemeren (Amsterdam: Sic Sac), 65-82.

O’Keefe, D. J., and Jensen, J. D. (2006). The advantages of compliance or the disadvantages of noncompliance? A meta-analytic review of the relative persuasive effectiveness of gain-framed and loss-framed messages. *Ann. Int. Commun. Assoc.* 30(1), 1-43. doi:10.1080/23808985.2006.11679054

O’Keefe, D. J., and Jensen, J. D. (2007). The relative persuasiveness of gain-framed and loss-framed messages for encouraging disease prevention behaviors: A meta-analytic review. *J. Health Commun.* 12*,* 623-644. doi.10.1080/10810730701615198

O’Keefe, D. J., and Jensen, J. D. (2009). The relative persuasiveness of gain-framed and loss-framed messages for encouraging disease detection behaviors: A meta-analytic review. *J. Commun.* 59, 296-316. doi.10.1111/j.1460-2466.2009.01417.x

Peters, G.-J. Y., Ruiter, R. A. C., and Kok, G. (2013). Threatening communication: A critical re-analysis and a revised meta-analytic test of fear appeal theory. *Health Psychol. Rev.* 7(suppl. 1), S8–S31. doi.10.1080/17437199.2012.703527

Piñon, A., and Gambara, H. (2005). A meta-analytic review of framing effect: Risky, attribute and goal framing. *Psicothema* 17, 325-331.

Preiss, R. W., Allen, M., Gayle, B., and Kim, S.-Y. (2014, November). Meta-analysis of the relationship between rate of delivery and message persuasiveness: Linear versus curvilinear tests. [Paper presentation]. Natl. Commun. Assoc. annual meeting, Chicago, IL.

Reinhart, A. M. (2006). Comparing the persuasive effects of narrative versus statistical messages: A meta-analytic review. [Doctoral dissertation]. [Buffalo (BY)]: State University of New York at Buffalo. ProQuest no. 3213634.

Reinhart, A. M., and Feeley, T. (2007, November). Comparing the persuasive effects of narrative versus statistical messages: A meta-analytic review. [Paper presentation]. Nat. Commun. Assoc. annual meeting, Chicago, IL.

Schumacher, A. C. (2017). Humor in public health messaging: Past, present, future. [Doctoral dissertation]. [Iowa City (IA)]: University of Iowa. ProQuest no. 10259441.

Seo, K. (2020). Meta-analysis on visual persuasion: Does adding images to texts influence persuasion? *Athens J. Mass Media Commun.* 6(3), 177-190. doi:10.30958/ajmmc.6-3-3

Seo, K., and Kim, N. Y. (2018, May). Does adding images to texts influence persuasion? A meta-analysis of visual image effects on persuasive texts. [Paper presentation]. Intl. Commun. Assoc. annual meeting, Prague, Czech Republic.

Shen, F., Sheer, V. C., and Li, R. (2015). Impact of narratives on persuasion in health communication: A meta-analysis. *J. Advert.* 44(2), 105-113. doi:10.1080/00913367.2015.1018467

Sopory, P., and Dillard, J. P. (2002). The persuasive effects of metaphor: A meta-analysis. *Hum. Commun. Res.* 28, 382-419. doi.10.1111/j.1468-2958.2002.tb00813.x

Stiff, J. B. (1985). Cognitive processing of persuasive message cues: A meta-analytic review of the effects of supporting information on attitudes. [Doctoral dissertation]. [East Lansing (MI)]: Michigan State University. ProQuest no. 8520567.

Stiff, J. B. (1986). Cognitive processing of persuasive message cues: A meta-analytic review of the effects of supporting information on attitudes. *Commun. Monog.* 53(1), 75-89. doi:10.1080/03637758609376128

Sutton, S. R. (1982). Fear-arousing communications: A critical examination of theory and research. In *Social Psychology and Behavioral Medicine,* ed. J. R. Eiser (New York: Wiley), 303-337.

Van Stee, S. K. (2018). Meta-analysis of the persuasive effects of metaphorical vs. literal messages. *Commun. Stud.*69, 545-566. doi.10.1080/10510974.2018.1457553

Walter, N., Cody, M. J., Xu, L. Z., and Murphy, S. T. (2018). A priest, a rabbi, and a minister walk into a bar: A meta-analysis of humor effects on persuasion. *Hum. Commun. Res.*44(4), 343-373. doi:10.1093/hcr/hqy005

White, B. X., and Albarracín, D. (2018). Investigating belief falsehood: Fear appeals do change behavior in experimental laboratory studies: A commentary on Kok et al. (2018). *Health Psychol. Rev.* 12(2), 147-150. doi:10.1080/17437199.2018.1448292

Wirtz, J. G., Sparks, J. V., and Zimbres, T. M. (2018). The effect of exposure to sexual appeals in advertisements on memory, attitude, and purchase intention: A meta-analytic review. *Int. J. Advert.* 37, 168-198. doi.10.1080/02650487.2017.1334996

Witte, K., and Allen, M. (2000). A meta-analysis of fear appeals: Implications for effective public health programs. *Health Educ. Behav.* 27(5), 591-615. doi:10.1177/109019810002700506

Zebregs, S., van den Putte, B., Neijens, P., and de Graaf, A. (2015). The differential impact of statistical and narrative evidence on beliefs, attitude, and intention: A meta-analysis. *Health Commun.* 30, 282-289. doi.10.1080/10410236.2013.842528

**APPENDIX 3**

ALL ONE-MODERATOR EFFECT SIZES:

MEANS, CONFIDENCE INTERVALS, AND PREDICTION INTERVALS^a^

| Variable | *k* | *N* | mean *r* | [95% CI] | 95% PI |
| --- | --- | --- | --- | --- | --- |
| Sidedness (two-sided vs. one-sided) |  |  |  |  |  |
| two-sided type: nonrefutational | 65 | 13,946 | -.049 | [-.091, .007] | -.335, .245 |
| two-sided type: refutational | 42 | 6,165 | .076 | [.031, .119] | -.140, .285 |
| topic: advertising | 35 | 7,538 | .001 | [-.054, .056] | -.291, .293 |
| topic: non-advertising | 72 | 12,573 | -.004 | [-.045, .037] | -.288, .280 |
| initial attitude: neutral | 33 | 5,271 | -.018 | [-.087, .050] | -.371, .340 |
| audience education: some college | 77 | 11,972 | -.020 | [-.065, .025] | -.352, .317 |
| counterargument availability: high | 70 | 13,232 | -.001 | [-.038, .036] | -.251, .249 |
| two-sided order: support-oppose | 18 | 2,583 | .022 | [-.045, .090] | -.227, .268 |
| two-sided order: oppose-support | 13 | 1,525 | -.024 | [-.097, .050] | -.222, .176 |
| two-sided order: interwoven | 35 | 6,828 | .000 | [-.074, .074] | -.392, .392 |
| Appeal framing (gain vs. loss) |  |  |  |  |  |
| topic: disease detection | 34 | 7,112 | -.027 | [-.072, .018] | -.222, .170 |
| topic: disease prevention: | 74 | 16,255 | .046 | [.015, .078] | -.145, .234 |
| topic: other health | 10 | 1,430 | -.038 | [-.092, .016] | -.101, .026 |
| topic: consumer advertising | 25 | 3,805 | -.013 | [-.073, .048] | -.268, .243 |
| topic: other | 19 | 21,754 | .060 | [.006, .113] | -.136, .252 |
| gain kernels: desirable | 36 | 23,277 | .022 | [-.018, .063] | -.152, .195 |
| gain kernels: undesirable | 19 | 9,431 | -.006 | [-.054, .042] | -.162, .151 |
| gain kernels: combination | 65 | 9,540 | -.002 | [-.036, .033] | -.202, .198 |
| loss kernels: desirable | 12 | 1,679 | .097 | [-.006, .198] | -.267, .437 |
| loss kernels: undesirable | 49 | 31,917 | -.012 | [-.039, .014] | -.105, .082 |
| loss kernels: combination | 61 | 8,934 | .007 | [-.031, .044] | -.212, .225 |
| Victim description (identifiable vs. non-identifiable) |  |  |  |  |  |
| age: adult | 13 | 665 | -.010 | [-.136, .117] | -.414, .397 |
| age: child | 28 | 15,302 | .073 | [.018, .128] | -.140, .279 |
| cause: disease-injury | 22 | 1,782 | .022 | [-.057, .102] | -.278, .318 |
| cause: poverty | 19 | 14,185 | .084 | [.010, .157] | -.176, .333 |
| group: in-group | 26 | 2,141 | .038 | [-.034, .110] | -.258, .327 |
| group: out-group | 15 | 13,826 | .075 | [-.009, .157] | -.194, .333 |
| identified victim: single | 33 | 2,715 | .095 | [.033, .156] | -.176, .353 |
| location: non-U.S. | 31 | 15,230 | .033 | [-.022, .089] | -.187, .250 |
| location: U.S. | 10 | 737 | .116 | [-.002, .232] | -.241, .446 |
| monetary goal: absent | 33 | 15,466 | .056 | [.003, .109] | -.163, .270 |
| picture: not present | 12 | 772 | .014 | [-.110, .138] | -.378, .401 |
| picture: present | 29 | 15,195 | .065 | [.008, .121] | -.157, .281 |
| responsibility: not responsible | 36 | 15,708 | .061 | [.009, .113] | -.158, .274 |
| sex: female-only | 13 | 1,263 | .088 | [-.013, .187] | -.231, .390 |
| sex: male-only | 13 | 998 | .034 | [-.062, .129] | -.258, .321 |
| unidentified victim: group | 26 | 14,952 | .014 | [-.042, .071] | -.189, .215 |
| unidentified victim: single | 15 | 1,015 | .127 | [.028, .223] | -.208, .435 |
| urgency: absent | 27 | 2,156 | .056 | [-.014, .126] | -.226, .329 |
| urgency: present | 14 | 13,811 | .046 | [-.039, .130] | -.225, .310 |
| Visual material (text-plus-visual vs. text-only) |  |  |  |  |  |
| visual type: photograph | 14 | 2,065 | .078 | [.007, .148] | -.152, .300 |
| visual valence: neutral | 10 | 991 | .043 | [-.085, .168] | -.349, .422 |
| Narrative (narrative vs. non-narrative) |  |  |  |  |  |
| behavior: prevention | 21 | 3,443 | .076 | [.027, .125] | -.094, .242 |
| medium: print | 22 | 5,434 | .055 | [.005, .106] | -.127, .234 |
| participants: students | 14 | 2,154 | .077 | [.009, .145] | -.142, .289 |
| participants: nonstudents | 20 | 7,176 | .060 | [.012, .108] | -.110, .227 |
| participant sex: both | 22 | 5,713 | .055 | [.011, .099] | -.085, .193 |
| participant sex: female | 12 | 3,617 | .085 | [.013, .157] | -.161, .321 |
| Metaphorical (vs. non-metaphorical) |  |  |  |  |  |
| metaphor type: conceptual | 36 | 14,667 | .114 | [.075, .153] | -.093, .311 |
| metaphor type: linguistic | 55 | 20,116 | .041 | [.014, .067] | -.116, .196 |
| policy: domestic | 63 | 24,764 | .065 | [.039, .090] | -.104, .230 |
| policy: foreign | 28 | 10,019 | .083 | [.033, .132] | -.164, .320 |
| topic: economics | 31 | 15,229 | .059 | [.025, .093] | -.103, .218 |
| topic: health and environment | 32 | 11,820 | .069 | [.022, .115] | -.167, .298 |
| topic: science and education | 17 | 3,980 | .029 | [-.003, .060] | -.005, .063 |
| topic: security and justice | 28 | 11,039 | .050 | [.021, .079] | -.067, .165 |
| Cultural tailoring (deep-tailored vs. not-tailored) |  |  |  |  |  |
| region: North America | 22 | 2,256 | .096 | [.010, .181] | -.270, .438 |
| region: Europe | 20 | 1,829 | .034 | [-.032, .100] | -.195, .259 |
| region: Asia-Pacific | 19 | 1,609 | .124 | [.020, .225] | -.302, .509 |
| value: individualism-collectivism | 41 | 4,406 | .105 | [.046, .163] | -.223, .411 |
| value: masculinity-femininity | 13 | 950 | .015 | [-.075, .105] | -.250, .278 |
| Depicted threat severity (high vs. low) |  |  |  |  |  |
| images: absent | 26 | 4,307 | .120 | [.058, .180] | -.170, .390 |
| images: present | 29 | 4,507 | .114 | [.071, .157] | -.063, .284 |
| Humor (humorous vs. non-humorous) |  |  |  |  |  |
| channel: audiovisual | 21 | 3,711 | .094 | [.012, .174] | -.260, .426 |
| channel: print | 10 | 1,620 | .041 | [-.147, .227] | -.583, .635 |
| channel: visual | 15 | 3,041 | .187 | [.056, .311] | -.345, .628 |
| delay: no delay | 53 | 9,322 | .123 | [.058, .186] | -.319, .521 |
| involvement: high | 20 | 2,835 | .106 | [-.020, .230] | -.444, .598 |
| involvement: low | 35 | 6,629 | .130 | [.060, .200] | -.266, .489 |
| sample: students | 50 | 8,574 | .110 | [.045, .174] | -.315, .498 |
| source sex: male | 21 | 3,129 | .181 | [.062, .294] | -.361, .631 |
| style: parody | 12 | 1,848 | .221 | [.100, .336] | -.233, .596 |
| style: satire | 12 | 1,950 | .027 | [-.065, .118] | -.282, .331 |
| style: surprise | 11 | 2,493 | .059 | [-.090, .205] | -.468, .555 |
| topic: health | 17 | 3,703 | .028 | [-.089, .145] | -.446, .489 |
| topic: marketing | 19 | 3,639 | .241 | [.161, .318] | -.112, .540 |
| topic: politics | 17 | 2,574 | .035 | [-.047, .116] | -.272, .336 |
| Vividness (vivid vs. pallid) |  |  |  |  |  |
| congruency: congruent | 21 | 2,448 | .146 | [.064, .226] | -.199, .459 |
| valence: positive | 11 | 1,836 | .276 | [.182, .364] | -.041, .542 |
| valence: negative | 15 | 1,573 | .080 | [-.009, .168] | -.222, .368 |
| manipulation: concrete | 21 | 3,032 | .135 | [.068, .202] | -.138, .389 |
| topic: commercial | 19 | 2,643 | .171 | [.093, .247] | -.144, .454 |
| topic: health | 15 | 1,581 | .116 | [.045, .185] | -.092, .314 |
| “That’s not all” (included vs. omitted) |  |  |  |  |  |
| behavior: product purchase: | 14 | 687 | .230 | [.133, .323] | -.011, .446 |
| negotiation message: included | 10 | 699 | .197 | [.063, .323] | -.232, .562 |
| Depicted threat vulnerability (high vs. low) |  |  |  |  |  |
| images: absent | 23 | 3,186 | .217 | [.143, .289] | -.130, .517 |
| “But you are free” (included vs. omitted) |  |  |  |  |  |
| requester: present | 32 | 13,434 | .203 | [.168, .237] | .057, .341 |
| requester: absent | 10 | 8,799 | .088 | [.028, .148] | -.104, .274 |
| beneficiary: selfish | 19 | 11,347 | .195 | [.149, .241] | .026, .353 |
| beneficiary: pro-social | 21 | 6,334 | .166 | [.113, .217] | -.039, .357 |
| Argument strength (strong vs. weak) |  |  |  |  |  |
| processing route: central | 13 | 842 | .312 | [.209, .409] | -.034, .591 |
| processing route: peripheral | 13 | 842 | .112 | [-.004, .226] | -.266, .460 |
| Disrupt-then-reframe (vs. reframe-only) |  |  |  |  |  |
| context: nonprofit | 10 | 731 | .321 | [.253, .386] | .241, .397 |

^a^Note: *k*: number of effect sizes; CI: confidence interval; PI: prediction interval.

**APPENDIX 4**

ALL TWO-MODERATOR EFFECT SIZES:

MEANS, CONFIDENCE INTERVALS, AND PREDICTION INTERVALS^a^

| Variable | *k* | *N* | mean *r* | [95% CI] | 95% PI |
| --- | --- | --- | --- | --- | --- |
| Sidedness (two-sided vs. one-sided) |  |  |  |  |  |
| attitude: neutral and counterargument availability: high | 27 | 4,498 | -.008 | [-.084, .067] | -.367, .353 |
| attitude: neutral and education: some college | 19 | 3,319 | -.057 | [-.156, .043] | -.468, .374 |
| counterargument availability: high and two-sided order: interwoven | 19 | 4,456 | -.010 | [-.106, .086] | -.395, .378 |
| counterargument availability: high and two-sided order: support-oppose | 16 | 2,279 | .022 | [-.052, .095] | -.231, .272 |
| education: some college and counterargument availability: high | 49 | 7,024 | -.019 | [-.071. .032] | -.317, .283 |
| education: some college and two-sided order: interwoven | 26 | 4,185 | -.031 | [-.129, .068] | -.482, .433 |
| education: some college and two-sided order: oppose-support | 10 | 630 | -.049 | [-.165, .069] | -.370, .282 |
| education: some college and two-sided order: support-oppose | 12 | 1,530 | .044 | [-.027, .116] | -.158, .243 |
| topic: advertising and attitude: neutral | 26 | 4,471 | -.015 | [-.091, .062] | -.374, .348 |
| topic: advertising and counterargument availability: high | 33 | 7,458 | -.008 | [-.064, .048] | -.293, .279 |
| topic: advertising and education: some college | 15 | 2,990 | -.042 | [-.151, .067] | -.459, .390 |
| topic: nonadvertising and counterargument availability: high | 37 | 5,774 | .006 | [-.043, .055] | -.210, .221 |
| topic: nonadvertising and education: some college | 62 | 8,982 | -.014 | [-.063, .035] | -.333, .308 |
| topic: nonadvertising and two-sided order: interwoven | 27 | 5,549 | .009 | [-.068, .087] | -.348, .363 |
| topic: nonadvertising and two-sided order: oppose-support | 10 | 779 | -.029 | [-.138, .081] | -.327, .275 |
| topic: nonadvertising and two-sided order: support-oppose | 10 | 1,146 | .029 | [-.040, .099] | -.121, .178 |
| two-sided type: nonrefutational and attitude: neutral | 21 | 3,346 | -.057 | [-.153, .040] | -.465, .371 |
| two-sided type: nonrefutational and counterargument availability: high | 47 | 10,591 | -.038 | [-.081, .006] | -.281, .210 |
| two-sided type: nonrefutational and education: some college | 46 | 7,174 | -.071 | [-.131, -.011] | -.416, .291 |
| two-sided type: nonrefutational and topic: advertising | 26 | 6,099 | -.020 | [-.084, .044] | -.315, .279 |
| two-sided type: nonrefutational and topic: nonadvertising | 39 | 7,847 | -.070 | [-.126, -.014] | -.359, .232 |
| two-sided type: nonrefutational and two-sided order: interwoven | 23 | 5,375 | -.058 | [-.153, .039] | -.474, .380 |
| two-sided type: nonrefutational and two-sided order: support-oppose | 11 | 1,509 | -.031 | [-.125, .063] | -.325, .268 |
| two-sided type: refutational and attitude: neutral | 12 | 1,925 | .042 | [-.044, .127] | -.243, .320 |
| two-sided type: refutational and counterargument availability: high | 23 | 2,641 | .085 | [.018, .152] | -.185, .343 |
| two-sided type: refutational and education: some college | 31 | 4,798 | .062 | [.007, .116] | -.176, .293 |
| two-sided type: refutational and topic: nonadvertising | 33 | 4,726 | .082 | [.035, .127] | -.106, .264 |
| two-sided type: refutational and two-sided order: interwoven | 12 | 1,453 | .105 | [.036, .172] | -.070, .274 |
| Appeal framing (gain vs. loss) |  |  |  |  |  |
| gain kernels: combined and loss kernels: combined | 48 | 6,672 | -.010 | [-.054, .033] | -.239, .220 |
| gain kernels: combined and loss kernels: undesirable | 15 | 2,458 | -.008 | [-.056, .040] | -.118, .102 |
| gain kernels: combined and topic: detection | 14 | 1,651 | -.011 | [-.090, .068] | -.259. .239 |
| gain kernels: combined and topic: prevention | 34 | 4,751 | .031 | [-.017, .080] | -.177, .236 |
| gain kernels: desirable and loss kernels: desirable | 10 | 1,269 | .090 | [-.038, .216] | -.346, .494 |
| gain kernels: desirable and loss kernels: undesirable | 17 | 20,568 | -.007 | [-.058, .044] | -.166, .152 |
| gain kernels: desirable and topic: consumer | 14 | 2,201 | .023 | [-.048, .093] | -.195, .239 |
| gain kernels: desirable and topic: prevention | 11 | 1,291 | .078 | [.004, .151] | -.103, .254 |
| gain kernels: undesirable and loss kernels: undesirable | 17 | 8,891 | -.025 | [-.075, .025] | -.169, .120 |
| gain kernels: undesirable and topic: prevention | 12 | 8,168 | .036 | [-.022, .093] | -.119, .189 |
| loss kernels: combined and topic: consumer | 10 | 1,153 | -.060 | [-.165, .047] | -.388, .282 |
| loss kernels: combined and topic: detection | 12 | 1,431 | -.025 | [-.116, .066] | -.299, .253 |
| loss kernels: combined and topic: prevention | 31 | 4,759 | .051 | [-.003, .104] | -.186, .283 |
| loss kernels: undesirable and topic: consumer | 11 | 1,944 | -.040 | [-.111, .032] | -.242, .165 |
| loss kernels: undesirable and topic: prevention | 22 | 9,073 | .023 | [-.021, .067] | -.100, .145 |
| Victim description (identifiable vs. non-identifiable) |  |  |  |  |  |
| age: adult and cause: disease | 11 | 571 | -.007 | [-.138, .124] | -.398, .386 |
| age: adult and group: in-group | 11 | 571 | .018 | [-.125, .160] | -.420, .449 |
| age: adult and identified victim: single | 11 | 551 | .021 | [-.121, .162] | -.412, .447 |
| age: adult and location: non-U.S. | 13 | 665 | -.010 | [-.136, .117] | -.414, .397 |
| age: adult and monetary goal: absent | 13 | 665 | -.010 | [-.136, .117] | -.414, .397 |
| age: adult and picture: not present | 10 | 500 | -.002 | [-.159, .154] | -.474, .471 |
| age: adult and urgency: absent | 11 | 571 | -.007 | [-.138, .124] | -.398, .386 |
| age: child and cause: disease | 11 | 1,211 | .046 | [-.056, .148] | -.273, .356 |
| age: child and cause: poverty | 17 | 14,091 | .093 | [.018, .166] | -.153, .328 |
| age: child and group: in-group | 15 | 1,595 | .050 | [-.034, .133] | -.234, .326 |
| age: child and group: out-group | 13 | 13,707 | .104 | [.015, .192] | -.173, .366 |
| age: child and identified victim: single | 22 | 2,164 | .121 | [.055, .187] | -.126, .354 |
| age: child and location: non-U.S. | 18 | 14,565 | .054 | [-.010, .117] | -.157, .260 |
| age: child and location: U.S. | 10 | 737 | .116 | [-.002, .232] | -.241, .446 |
| age: child and monetary goal: absent | 20 | 14,801 | .083 | [.023, .143] | -.126, .285 |
| age: child and picture: present | 26 | 15,030 | .073 | [.014, .132] | -.151, .290 |
| age: child and responsibility: not responsible | 28 | 15,302 | .073 | [.018, .128] | -.140, .279 |
| age: child and sex: female-only | 11 | 1,169 | .102 | [.003, .200] | -.195, .382 |
| age: child and unidentified victim: group | 19 | 14,579 | .036 | [-.026, .097] | -.162, .232 |
| age: child and urgency: absent | 16 | 1,585 | .092 | [.009, .173] | -.183, .354 |
| age: child and urgency: present | 12 | 13,717 | .054 | [-.033, .139] | -.206, .307 |
| cause: disease and group: in-group | 20 | 1,663 | .039 | [-.044, .121] | -.265, .336 |
| cause: disease and identified victim: single | 17 | 1,472 | .074 | [-.011, .158] | -.215, .351 |
| cause: disease and location: non-U.S. | 22 | 1,782 | .022 | [-.057, .102] | -.278, .318 |
| cause: disease and monetary goal: absent | 16 | 1,393 | .018 | [-.072, .107] | -.271, .304 |
| cause: disease and picture: picture | 14 | 1,376 | .033 | [-.058, .123] | -.262, .322 |
| cause: disease and responsibility: not responsible | 19 | 1,617 | .030 | [-.056, .116] | -.282, .336 |
| cause: disease and sex: male-only | 11 | 726 | .022 | [-.094, .138] | -.327, .366 |
| cause: disease and unidentified victim: single | 13 | 875 | .102 | [-.006, .208] | -.248, .428 |
| cause: disease and urgency: absent | 12 | 1,003 | -.002 | [-.109, .104] | -.314, .310 |
| cause: disease and urgency: present | 10 | 779 | .049 | [-.076, .173] | -.343, .427 |
| cause: poverty and group: out-group | 13 | 13,707 | .104 | [.015, .192] | -.173, .366 |
| cause: poverty and identified victim: single | 16 | 1,243 | .116 | [.024, .206] | -.200, .411 |
| cause: poverty and location: U.S. | 10 | 737 | .116 | [-.002, .232] | -.241, .446 |
| cause: poverty and monetary goal: absent | 17 | 14,073 | .089 | [.012, .164] | -.166, .333 |
| cause: poverty and picture: present | 15 | 13,819 | .096 | [.012, .180] | -.183, .360 |
| cause: poverty and responsibility: not responsible | 17 | 14,091 | .093 | [.018, .166] | -.153, .328 |
| cause: poverty and sex: female-only | 12 | 831 | .096 | [-.023, .213] | -.286, .452 |
| cause: poverty and unidentified victim: group | 17 | 14,045 | .062 | [-.012, .136] | -.174, .291 |
| cause: poverty and urgency: absent | 15 | 1,153 | .100 | [.006, .191] | -.211, .393 |
| group: in-group and identified victim: single | 21 | 1,833 | .080 | [.002, .156] | -.209, .356 |
| group: in-group and location: non-U.S. | 26 | 2,141 | .038 | [-.034, .110] | -.258, .327 |
| group: in-group and monetary goal: absent | 18 | 1,640 | .040 | [-.042, .121] | -.245, .319 |
| group: in-group and picture: absent | 10 | 653 | .048 | [-.089, .183] | -.365, .445 |
| group: in-group and picture: present | 16 | 1,488 | .033 | [-.055, .120] | -.264, .324 |
| group: in-group and responsibility: not responsible | 21 | 1,882 | .052 | [-.026, .129] | -.236, .332 |
| group: in-group and sex: male-only | 13 | 998 | .034 | [-.062, .129] | -.258, .321 |
| group: in-group and unidentified victim: group | 13 | 1,271 | -.039 | [-.126, .049] | -.291, .218 |
| group: in-group and unidentified victim: single | 13 | 870 | .124 | [.016, .230] | -.227, .447 |
| group: in-group and urgency: absent | 13 | 1,132 | .024 | [-.074, .121] | -.261, .305 |
| group: in-group and urgency: present | 13 | 1,009 | .051 | [-.061, .161] | -.326, .414 |
| group: out-group and identified victim: single | 12 | 882 | .121 | [.015, .224] | -.213, .429 |
| group: out-group and location: U.S. | 10 | 737 | .116 | [-.002, .232] | -.241, .446 |
| group: out-group and monetary goal: absent | 15 | 13,826 | .075 | [-.009, .157] | -.194, .333 |
| group: out-group and picture: present | 13 | 13,707 | .104 | [.015, .192] | -.173, .366 |
| group: out-group and responsibility: not responsible | 15 | 13,826 | .075 | [-.009, .157] | -.194, .333 |
| group: out-group and sex: female-only | 10 | 737 | .116 | [-.002, .232] | -.241, .446 |
| group: out-group and unidentified victim: group | 13 | 13,681 | .064 | [-.024, .151] | -.203, .322 |
| group: out-group and urgency: absent | 14 | 1,024 | .085 | [-.018, .187] | -.265, .415 |
| identified victim: single and location: non-U.S. | 23 | 1,978 | .085 | [.011, .158] | -.202, .358 |
| identified victim: single and location: U.S. | 10 | 737 | .116 | [-.002, .232] | -.241, .446 |
| identified victim: single and monetary goal: absent | 29 | 2,466 | .076 | [.010, .142] | -.205, .345 |
| identified victim: single and picture: not present | 10 | 658 | .051 | [-.084, .184] | -.356, .442 |
| identified victim: single and picture: present | 23 | 2,057 | .111 | [.041, .179] | -.152, .360 |
| identified victim: single and responsibility: not responsible | 28 | 2,456 | .112 | [.049, .175] | -.147, .357 |
| identified victim: single and sex: female-only | 13 | 1,263 | .088 | [-.013, .187] | -.231, .390 |
| identified victim: single and sex: male-only | 13 | 998 | .034 | [-.062, .129] | -.258, .321 |
| identified victim: single and unidentified victim: group | 18 | 1,700 | .069 | [-.010, .148] | -.202, .330 |
| identified victim: single and unidentified victim: single | 15 | 1,015 | .127 | [.028, .223] | -.208, .435 |
| identified victim: single and urgency: absent | 23 | 1,902 | .088 | [.013, .161] | -.192, .354 |
| identified victim: single and urgency: present | 10 | 813 | .109 | [-.007, .222] | -.246, .439 |
| location: non-U.S. and monetary goal: absent | 23 | 14,729 | .033 | [-.028, .093] | -.173, .236 |
| location: non-U.S. and picture: not present | 12 | 772 | .014 | [-.110, .138] | -.378, .401 |
| location: non-U.S. and picture: present | 19 | 14,458 | .041 | [-.024, .106] | -.169, .248 |
| location: non-U.S. and responsibility: not responsible | 26 | 14,971 | .042 | [-.016, .100] | -.172, .252 |
| location: non-U.S. and sex: male-only | 13 | 998 | .034 | [-.062, .129] | -.258, .321 |
| location: non-U.S. and unidentified victim: group | 16 | 14,215 | -.033 | [-.095, .029] | -.197, .132 |
| location: non-U.S. and unidentified victim: single | 15 | 1,015 | .127 | [.028, .223] | -.208, .435 |
| location: non-U.S. and urgency: absent | 17 | 1,419 | .021 | [-.066, .108] | -.266, .305 |
| location: non-U.S. and urgency: present | 14 | 13,811 | .046 | [-.039, .130] | -.225, .310 |
| location: U.S. and monetary goal: absent | 10 | 737 | .116 | [-.002, .232] | -.241, .446 |
| location: U.S. and picture: present | 10 | 737 | .116 | [-.002, .232] | -.241, .446 |
| location: U.S. and responsibility: not responsible | 10 | 737 | .116 | [-.002, .232] | -.241, .446 |
| location: U.S. and sex: female-only | 10 | 737 | .116 | [-.002, .232] | -.241, .446 |
| location: U.S. and unidentified victim: group | 10 | 737 | .116 | [-.002, .232] | -.241, .446 |
| location: U.S. and urgency: absent | 10 | 737 | .116 | [-.002, .232] | -.241, .446 |
| monetary goal: absent and picture: not present | 12 | 772 | .014 | [-.110, .138] | -.378, .401 |
| monetary goal: absent and picture: present | 21 | 14,694 | .073 | [.011, .134] | -.135, .275 |
| monetary goal: absent and responsibility: not responsible | 28 | 15,207 | .067 | [.011, .123] | -.146, .274 |
| monetary goal: absent and sex: female-only | 13 | 1,263 | .088 | [-.013, .187] | -.231, .390 |
| monetary goal: absent and sex: male-only | 13 | 998 | .034 | [-.062, .129] | -.258, .321 |
| monetary goal: absent and unidentified victim: group | 22 | 14,700 | .041 | [-.019, .101] | -.155, .234 |
| monetary goal: absent and unidentified victim: single | 11 | 766 | .088 | [-.037, .211] | -.313, .462 |
| monetary goal: absent and urgency: absent | 25 | 2,044 | .058 | [-.014, .130] | -.226, .333 |
| picture: not present and responsibility: not responsible | 10 | 678 | .022 | [-.106, .149] | -.356, .394 |
| picture: present and responsibility: not responsible | 26 | 15,030 | .073 | [.014, .132] | -.151, .290 |
| picture: present and sex: female-only | 11 | 1,169 | .102 | [.003, .200] | -.195, .382 |
| picture: present and unidentified victim: group | 20 | 14,472 | .023 | [-.040, .086] | -.175, .219 |
| picture: present and urgency: absent | 18 | 1,614 | .082 | [.001, .162] | -.197, .349 |
| picture: present and urgency: present | 11 | 13,581 | .041 | [-.051, .132] | -.233, .309 |
| responsibility: not responsible and sex: female-only | 11 | 1,169 | .102 | [.003, .200] | -.195, .382 |
| responsibility: not responsible and sex: male-only | 10 | 833 | .050 | [-.060, .158] | -.270, .360 |
| responsibility: not responsible and unidentified victim: group | 21 | 14,693 | .021 | [-.039, .081] | -.175, .215 |
| responsibility: not responsible and unidentified victim: single | 15 | 1,015 | .127 | [.028, .223] | -.208, .435 |
| responsibility: not responsible and urgency: absent | 24 | 1,991 | .066 | [-.009, .140] | -.227, .348 |
| responsibility: not responsible and urgency: present | 12 | 13,717 | .054 | [-.033, .139] | -.206, .307 |
| sex: female-only and unidentified victim: group | 13 | 1,263 | .088 | [-.013, .187] | -.231, .390 |
| sex: female-only and urgency: absent | 11 | 1,169 | .102 | [.003, .200] | -.195, .382 |
| unidentified victim: group and urgency: absent | 19 | 1,724 | .032 | [-.045, .107] | -.226, .286 |
| Narrative (narrative vs. non-narrative) |  |  |  |  |  |
| behavior: prevention and medium: print | 14 | 1,566 | .075 | [.003, .146] | -.145, .288 |
| behavior: prevention and participants: nonstudents | 10 | 1,554 | .053 | [-.020, .125] | -.130, .233 |
| behavior: prevention and participants: students | 11 | 1,889 | .095 | [.025, .165] | -.122, .304 |
| behavior: prevention and participant sex: both | 14 | 1,789 | .068 | [.003, .132] | -.115, .246 |
| medium: print and participants: nonstudents | 12 | 4,090 | .024 | [-.042, .089] | -.162, .209 |
| medium: print and participants: students | 10 | 1,344 | .094 | [.016, .171] | -.131, .310 |
| medium: print and participant sex: both | 17 | 4,912 | .048 | [-.006, .101] | -.126, .219 |
| participants: nonstudents and participant sex: both | 14 | 4,724 | .045 | [-.004, .094] | -.086, .175 |
| Metaphorical (vs. non-metaphorical) |  |  |  |  |  |
| metaphor type: conceptual and policy: domestic | 23 | 10,644 | .125 | [.071, .179] | -.118, .354 |
| metaphor type: conceptual and policy: foreign | 13 | 4,023 | .095 | [.047, .143] | -.054, .240 |
| metaphor type: conceptual and topic: economics | 14 | 7,539 | .110 | [.046, .173] | -.128, .336 |
| metaphor type: conceptual and topic: health and environment | 15 | 4,864 | .077 | [.022, .132] | -.113, .262 |
| metaphor type: conceptual and topic: security and justice | 12 | 4,759 | .075 | [.025, .124] | -.076, .223 |
| metaphor type: linguistic and policy: domestic | 40 | 14,120 | .021 | [.004, .037] | .004, .038 |
| metaphor type: linguistic and policy: foreign | 15 | 5,996 | .074 | [-.007, .153] | -.246, .379 |
| metaphor type: linguistic and topic: economics | 17 | 7,690 | .016 | [-.006, .039] | -.009, .041 |
| metaphor type: linguistic and topic: health and environment | 17 | 6,956 | .064 | [-.008, .135] | -.235, .352 |
| metaphor type: linguistic and topic: science and education | 12 | 3,142 | .019 | [-.016, .055] | -.022, .060 |
| metaphor type: linguistic and topic: security and justice | 16 | 6,280 | .029 | [-.003, .061] | -.047, .105 |
| policy: domestic and topic: economics | 22 | 12,944 | .060 | [.019, .101] | -.119, .236 |
| policy: domestic and topic: health and environment | 21 | 6,985 | .055 | [.013, .098] | -.113, .220 |
| policy: domestic and topic: science and education | 11 | 1,933 | .034 | [-.011, .079] | -.018, .086 |
| policy: domestic and topic: security and justice | 20 | 7,745 | .030 | [-.001, .061] | -.070, .129 |
| policy: foreign and topic: health and environment | 11 | 4,835 | .093 | [-.011, .196] | -.289, .450 |
| topic: economics and topic: health and environment | 10 | 3,460 | .045 | [-.014, .105] | -.146, .233 |
| topic: health and environment and topic: science and education | 12 | 3,030 | .026 | [-.010, .061] | -.014, .066 |
| Cultural tailoring (deep-tailored vs. not-tailored) |  |  |  |  |  |
| region: Asia-Pacific and value: individualism-collectivism | 17 | 1,509 | .123 | [.011, .233] | -.328, .528 |
| region: Europe and value: masculinity-femininity | 13 | 950 | .015 | [-.075, .105] | -.250, .278 |
| region: North American and value: individualism-collectivism | 19 | 1,860 | .125 | [.039, .210] | -.217, .440 |
| Humor (humorous vs. non-humorous) |  |  |  |  |  |
| channel: audiovisual and delay: no delay | 20 | 3,675 | .087 | [.004, .168] | -.269, .422 |
| channel: audiovisual and involvement: low | 11 | 2,260 | .045 | [-.045, .135] | -.262, .343 |
| channel: audiovisual and sample: students | 16 | 2,393 | .088 | [-.007, .181] | -.278, .432 |
| channel: visual and delay: no delay | 15 | 3,041 | .187 | [.056, .311] | -.345, .628 |
| channel: visual and sample: students | 15 | 3,041 | .187 | [.056, .311] | -.345, .628 |
| delay: no delay and involvement: high | 19 | 2,799 | .097 | [-.033, .223] | -.454, .594 |
| delay: no delay and involvement: low | 32 | 5,651 | .145 | [.067, .221] | -.282, .524 |
| delay: no delay and sample: students | 45 | 7,498 | .113 | [.041, .184] | -.339, .522 |
| delay: no delay and source sex: male | 18 | 2,887 | .182 | [.051, .307] | -.389, .652 |
| delay: no delay and style: parody | 12 | 1,848 | .221 | [.100, .336] | -.233, .596 |
| delay: no delay and style: surprise | 11 | 2,493 | .059 | [-.090, .205] | -.468, .555 |
| delay: no delay and topic: health | 16 | 2,961 | .027 | [-.105, .158] | -.492, .532 |
| delay: no delay and topic: marketing | 19 | 3,639 | .241 | [.161, .318] | -.112, .540 |
| delay: no delay and topic: political | 13 | 2,240 | .023 | [-.071, .116] | -.309, .350 |
| involvement: high and sample: students | 19 | 2,651 | .092 | [-.037, .217] | -.452, .586 |
| involvement: low and sample: students | 28 | 4,989 | .126 | [.048, .203] | -.270, .486 |
| involvement: low and source sex: male | 16 | 2,681 | .167 | [.036, .291] | -.370, .620 |
| involvement: low and style: satire | 10 | 1,360 | -.006 | [-.096, .084] | -.290, .279 |
| involvement: low and topic: marketing | 12 | 2,351 | .242 | [.120, .356] | -.232, .623 |
| involvement: low and topic: political | 13 | 2,326 | .002 | [-.074, .079] | -.249, .253 |
| sample: students and source sex: male | 18 | 2,158 | .202 | [.074, .323] | -.350, .650 |
| sample: students and style: parody | 10 | 1,477 | .239 | [.091, .377] | -.298, .661 |
| sample: students and topic: health | 15 | 3,375 | -.021 | [-.127, .086] | -.420, .385 |
| sample students and topic: marketing | 17 | 3,277 | .243 | [.156, .326] | -.127, .554 |
| sample: students and topic: political | 13 | 1,440 | .034 | [-.073, .139] | -.333, .392 |
| style: satire and topic: political | 12 | 1,950 | .027 | [-.065, .118] | -.282, .331 |
| Vividness (vivid vs. pallid) |  |  |  |  |  |
| congruency: congruent and topic: commercial | 11 | 1,259 | .147 | [.025, .265] | -.279, .525 |
| manipulation: concrete and topic: commercial | 10 | 1,707 | .189 | [.104, .272] | -.081, .433 |
| manipulation: concrete and valence: negative | 10 | 1,161 | .077 | [-.035, .186] | -.281, .416 |
| topic: commercial and valence: positive | 11 | 1,836 | .276 | [.182, .364] | -.041, .542 |
| topic: health and valence: negative | 13 | 1,369 | .109 | [.029, .187] | -.128, .334 |
| “But you are free” (included vs. omitted) |  |  |  |  |  |
| requester: present and beneficiary: prosocial | 14 | 4,317 | .196 | [.153, .239] | .089, .299 |
| requester: present and beneficiary: selfish | 18 | 9,117 | .204 | [.153, .255] | .020, .374 |

^a^Note: *k*: number of effect sizes; CI: confidence interval; PI: prediction interval.

**APPENDIX 5**

LARGEST ONE-MODERATOR AND TWO-MODERATOR EFFECT SIZES:

MEAN EFFECT SIZES, CONFIDENCE INTERVALS, AND PREDICTION INTERVALS^a^

| Message variation | *k* | *N* | mean *r* | [95% CI] | 95% PI |
| --- | --- | --- | --- | --- | --- |
| **Sidedness (two-sided vs. one-sided)** |  |  |  |  |  |
| one moderator: two-sided refutational | 42 | 6165 | .076 | [.031, .119] | -.140, .285 |
| two moderators: two-sided refutational and interwoven two-sided order | 12 | 1,453 | .105 | [.036, .172] | -.070, .274 |
| **Appeal framing (gain vs. loss)** |  |  |  |  |  |
| one moderator: desirable loss kernels | 12 | 1,679 | .097 | [-.006, .198] | -.267, .437 |
| two moderators: desirable loss kernels and desirable gain kernels | 10 | 1,269 | .090 | [-.038, .216] | -.346, .494 |
| **Victim description (identifiable vs. non-identifiable)** |  |  |  |  |  |
| one moderator: single unidentified victim | 15 | 1,015 | .127 | [.028, .223] | -.208, .435 |
| two moderators: single unidentified victim and non-US location (or single identified victim or victim not responsible) | 15 | 1,015 | .127 | [.028, .223] | -.208, .435 |
| **Visual material (text-plus-visual vs. text-only)** |  |  |  |  |  |
| one moderator: photograph | 14 | 2,065 | .078 | [.007, .148] | -.152, .300 |
| **Narrative (narrative vs. non-narrative)** |  |  |  |  |  |
| one moderator: female participants | 12 | 3,617 | .085 | [.013, .157] | -.161, .321 |
| two moderators: prevention behavior and student participants | 11 | 1,889 | .095 | [.025, .165] | -.122, .304 |
| **Metaphorical (vs. non-metaphorical)** |  |  |  |  |  |
| one moderator: conceptual metaphor | 36 | 14,667 | .114 | [.075, .153] | -.093, .311 |
| two moderators: conceptual metaphor and domestic policy | 23 | 10,644 | .125 | [.071, .179] | -.118, .354 |
| **Cultural tailoring (deep-tailored vs. not-tailored)** |  |  |  |  |  |
| one moderator: Asia-Pacific region | 19 | 1,609 | .124 | [.020, .225] | -.302, .509 |
| two moderators: North American region and individualism-collectivism values | 19 | 1,860 | .125 | [.039, .210] | -.217, .440 |
| **Depicted threat severity (high vs. low)** |  |  |  |  |  |
| one moderator: images absent | 26 | 4,307 | .120 | [.058, .180] | -.170, .390 |
| **Humor (humorous vs. non-humorous)** |  |  |  |  |  |
| one moderator: marketing topic | 19 | 3,639 | .241 | [.161, .318] | -.112, .540 |
| two moderators: marketing topic and student sample | 17 | 3,277 | .243 | [.156, .326] | -.127, .554 |
| **Vividness (vivid vs. pallid)** |  |  |  |  |  |
| one moderator: positive valence | 11 | 1,836 | .276 | [.182, .364] | -.041, .542 |
| two moderators: positive valence and commercial topic | 11 | 1,836 | .276 | [.182, .364] | -.041, .542 |
| **“That’s not all” (included vs. omitted)** |  |  |  |  |  |
| one moderator: product purchase behavior | 14 | 687 | .230 | [.133, .323] | -.011, .446 |
| **Depicted threat vulnerability (high vs. low)** |  |  |  |  |  |
| one moderator: images absent | 23 | 3,186 | .217 | [.143, .289] | -.130, .517 |
| **“But you are free” (included vs. omitted)** |  |  |  |  |  |
| one moderator: requester present | 32 | 13,434 | .203 | [.168, .237] | .057, .341 |
| two moderators: requester present and selfish beneficiary | 18 | 9,117 | .204 | [.153, .255] | .020, .374 |
| **Argument strength (strong vs. weak)** |  |  |  |  |  |
| one moderator: central-route processing | 13 | 842 | .312 | [.209, .409] | -.034, .591 |
| **Disrupt-then-reframe (vs. reframe-only)** |  |  |  |  |  |
| one moderator: nonprofit context | 10 | 731 | .321 | [.253, .386] | .241, .397 |

^a^Note: *k*: number of effect sizes; CI: confidence interval; PI: prediction interval.

**APPENDIX 6**

STATISTICALLY SIGNIFICANT ONE-MODERATOR EFFECT SIZES:

MEAN EFFECT SIZES, CONFIDENCE INTERVALS, AND PREDICTION INTERVALS^a^

| Message variation | *k* | *N* | mean *r* | [95% CI] | 95% PI |
| --- | --- | --- | --- | --- | --- |
| **Sidedness (two-sided vs. one-sided)** |  |  |  |  |  |
| two-sided type: nonrefutational | 65 | 13,946 | -.049 | [-.091, -.007] | -.335, .245 |
| two-sided type: refutational | 42 | 6,165 | .076 | [.031, .119] | -.140, .285 |
| **Appeal framing (gain vs. loss)** |  |  |  |  |  |
| topic: disease prevention: | 74 | 16,255 | .046 | [.015, .078] | -.145, .234 |
| topic: other | 19 | 21,754 | .060 | [.006, .113] | -.136, .252 |
| **Victim description (identifiable vs. non-identifiable)** |  |  |  |  |  |
| age: child | 28 | 15,302 | .073 | [.018, .128] | -.140, .279 |
| cause: poverty | 19 | 14,185 | .084 | [.010, .157] | -.176, .333 |
| identified victim: single | 33 | 2,715 | .095 | [.033, .156] | -.176, .353 |
| monetary goal: absent | 33 | 15,466 | .056 | [.003, .109] | -.163, .270 |
| picture: present | 29 | 15,195 | .065 | [.008, .121] | -.157, .281 |
| responsibility: not responsible | 36 | 15,708 | .061 | [.009, .113] | -.158, .274 |
| unidentified victim: single | 15 | 1,015 | .127 | [.028, .223] | -.208, .435 |
| **Visual material (text-plus-visual vs. text-only)** |  |  |  |  |  |
| visual type: photograph | 14 | 2,065 | .078 | [.007, .148] | -.152, .300 |
| **Narrative (narrative vs. non-narrative)** |  |  |  |  |  |
| behavior: prevention | 21 | 3,443 | .076 | [.027, .125] | -.094, .242 |
| medium: print | 22 | 5,434 | .055 | [.005, .106] | -.127, .234 |
| participants: students | 14 | 2,154 | .077 | [.009, .145] | -.142, .289 |
| participants: nonstudents | 20 | 7,176 | .060 | [.012, .108] | -.110, .227 |
| participant sex: both | 22 | 5,713 | .055 | [.011, .099] | -.085, .193 |
| participant sex: female | 12 | 3,617 | .085 | [.013, .157] | -.161, .321 |
| **Metaphorical (vs. non-metaphorical)** |  |  |  |  |  |
| metaphor type: conceptual | 36 | 14,667 | .114 | [.075, .153] | -.093, .311 |
| metaphor type: linguistic | 55 | 20,116 | .041 | [.014, .067] | -.116, .196 |
| policy: domestic | 63 | 24,764 | .065 | [.039, .090] | -.104, .230 |
| policy: foreign | 28 | 10,019 | .083 | [.033, .132] | -.164, .320 |
| topic: economics | 31 | 15,229 | .059 | [.025, .093] | -.103, .218 |
| topic: health and environment | 32 | 11,820 | .069 | [.022, .115] | -.167, .298 |
| topic: security and justice | 28 | 11,039 | .050 | [.021, .079] | -.067, .165 |
| **Cultural tailoring (deep-tailored vs. not-tailored)** |  |  |  |  |  |
| region: North America | 22 | 2,256 | .096 | [.010, .181] | -.270, .438 |
| region: Asia-Pacific | 19 | 1,609 | .124 | [.020, .225] | -.302, .509 |
| value: individualism-collectivism | 41 | 4,406 | .105 | [.046, .163] | -.223, .411 |
| **Depicted threat severity (high vs. low)** |  |  |  |  |  |
| images: absent | 26 | 4,307 | .120 | [.058, .180] | -.170, .390 |
| images: present | 29 | 4,507 | .114 | [.071, .157] | -.063, .284 |
| **Humor (humorous vs. non-humorous)** |  |  |  |  |  |
| channel: audiovisual | 21 | 3,711 | .094 | [.012, .174] | -.260, .426 |
| channel: visual | 15 | 3,041 | .187 | [.056, .311] | -.345, .628 |
| delay: no delay | 53 | 9,322 | .123 | [.058, .186] | -.319, .521 |
| involvement: low | 35 | 6,629 | .130 | [.060, .200] | -.266, .489 |
| sample: students | 50 | 8,574 | .110 | [.045, .174] | -.315, .498 |
| source sex: male | 21 | 3,129 | .181 | [.062, .294] | -.361, .631 |
| style: parody | 12 | 1,848 | .221 | [.100, .336] | -.233, .596 |
| topic: marketing | 19 | 3,639 | .241 | [.161, .318] | -.112, .540 |
| **Vividness (vivid vs. pallid)** |  |  |  |  |  |
| congruency: congruent | 21 | 2,448 | .146 | [.064, .226] | -.199, .459 |
| valence: positive | 11 | 1,836 | .276 | [.182, .364] | -.041, .542 |
| manipulation: concrete | 21 | 3,032 | .135 | [.068, .202] | -.138, .389 |
| topic: commercial | 19 | 2,643 | .171 | [.093, .247] | -.144, .454 |
| topic: health | 15 | 1,581 | .116 | [.045, .185] | -.092, .314 |
| **“That’s not all” (included vs. omitted)** |  |  |  |  |  |
| behavior: product purchase: | 14 | 687 | .230 | [.133, .323] | -.011, .446 |
| negotiation message: included | 10 | 699 | .197 | [.063, .323] | -.232, .562 |
| **Depicted threat vulnerability (high vs. low)** |  |  |  |  |  |
| images: absent | 23 | 3,186 | .217 | [.143, .289] | -.130, .517 |
| **“But you are free” (included vs. omitted)** |  |  |  |  |  |
| requester: present | 32 | 13,434 | .203 | [.168, .237] | .057, .341 |
| requester: absent | 10 | 8,799 | .088 | [.028, .148] | -.104, .274 |
| beneficiary: selfish | 19 | 11,347 | .195 | [.149, .241] | .026, .353 |
| beneficiary: pro-social | 21 | 6,334 | .166 | [.113, .217] | -.039, .357 |
| **Argument strength (strong vs. weak)** |  |  |  |  |  |
| processing route: central | 13 | 842 | .312 | [.209, .409] | -.034, .591 |
| **Disrupt-then-reframe (vs. reframe-only)** |  |  |  |  |  |
| context: nonprofit | 10 | 731 | .321 | [.253, .386] | .241, .397 |

^a^Note: *k*: number of effect sizes; CI: confidence interval; PI: prediction interval.

**APPENDIX 7**

STATISTICALLY SIGNIFICANT TWO-MODERATOR EFFECT SIZES:

MEAN EFFECT SIZES, CONFIDENCE INTERVALS, AND PREDICTION INTERVALS^a^

| Message variation | *k* | *N* | mean *r* | [95% CI] | 95% PI |
| --- | --- | --- | --- | --- | --- |
| **Sidedness (two-sided vs. one-sided)** |  |  |  |  |  |
| two-sided type: refutational and counterargument availability: high | 23 | 2,641 | .085 | [.018, .152] | -.185, .343 |
| two-sided type: refutational and education: some college | 31 | 4,798 | .062 | [.007, .116] | -.176, .293 |
| two-sided type: refutational and topic: nonadvertising | 33 | 4,726 | .082 | [.035, .127] | -.106, .264 |
| two-sided type: refutational and two-sided order: interwoven | 12 | 1,453 | .105 | [.036, .172] | -.070, .274 |
| **Appeal framing (gain vs. loss)** |  |  |  |  |  |
| gain kernels: desirable and topic: prevention | 11 | 1,291 | .078 | [.004, .151] | -.103, .254 |
| **Victim description (identifiable vs. non-identifiable)** |  |  |  |  |  |
| age: child and cause: poverty | 17 | 14,091 | .093 | [.018, .166] | -.153, .328 |
| age: child and group: out-group | 13 | 13,707 | .104 | [.015, .192] | -.173, .366 |
| age: child and identified victim: single | 22 | 2,164 | .121 | [.055, .187] | -.126, .354 |
| age: child and monetary goal: absent | 20 | 14,801 | .083 | [.023, .143] | -.126, .285 |
| age: child and picture: present | 26 | 15,030 | .073 | [.014, .132] | -.151, .290 |
| age: child and responsibility: not responsible | 28 | 15,302 | .073 | [.018, .128] | -.140, .279 |
| age: child and sex: female-only | 11 | 1,169 | .102 | [.003, .200] | -.195, .382 |
| age: child and urgency: absent | 16 | 1,585 | .092 | [.009, .173] | -.183, .354 |
| cause: poverty and group: out-group | 13 | 13,707 | .104 | [.015, .192] | -.173, .366 |
| cause: poverty and identified victim: single | 16 | 1,243 | .116 | [.024, .206] | -.200, .411 |
| cause: poverty and monetary goal: absent | 17 | 14,073 | .089 | [.012, .164] | -.166, .333 |
| cause: poverty and picture: present | 15 | 13,819 | .096 | [.012, .180] | -.183, .360 |
| cause: poverty and responsibility: not responsible | 17 | 14,091 | .093 | [.018, .166] | -.153, .328 |
| cause: poverty and urgency: absent | 15 | 1,153 | .100 | [.006, .191] | -.211, .393 |
| group: in-group and identified victim: single | 21 | 1,833 | .080 | [.002, .156] | -.209, .356 |
| group: in-group and unidentified victim: single | 13 | 870 | .124 | [.016, .230] | -.227, .447 |
| group: out-group and identified victim: single | 12 | 882 | .121 | [.015, .224] | -.213, .429 |
| group: out-group and picture: present | 13 | 13,707 | .104 | [.015, .192] | -.173, .366 |
| identified victim: single and location: non-U.S. | 23 | 1,978 | .085 | [.011, .158] | -.202, .358 |
| identified victim: single and monetary goal: absent | 29 | 2,466 | .076 | [.010, .142] | -.205, .345 |
| identified victim: single and picture: present | 23 | 2,057 | .111 | [.041, .179] | -.152, .360 |
| identified victim: single and responsibility: not responsible | 28 | 2,456 | .112 | [.049, .175] | -.147, .357 |
| identified victim: single and unidentified victim: single | 15 | 1,015 | .127 | [.028, .223] | -.208, .435 |
| identified victim: single and urgency: absent | 23 | 1,902 | .088 | [.013, .161] | -.192, .354 |
| location: non-U.S. and unidentified victim: single | 15 | 1,015 | .127 | [.028, .223] | -.208, .435 |
| monetary goal: absent and picture: present | 21 | 14,694 | .073 | [.011, .134] | -.135, .275 |
| monetary goal: absent and responsibility: not responsible | 28 | 15,207 | .067 | [.011, .123] | -.146, .274 |
| picture: present and responsibility: not responsible | 26 | 15,030 | .073 | [.014, .132] | -.151, .290 |
| picture: present and sex: female-only | 11 | 1,169 | .102 | [.003, .200] | -.195, .382 |
| picture: present and urgency: absent | 18 | 1,614 | .082 | [.001, .162] | -.197, .349 |
| responsibility: not responsible and sex: female-only | 11 | 1,169 | .102 | [.003, .200] | -.195, .382 |
| responsibility: not responsible and unidentified victim: single | 15 | 1,015 | .127 | [.028, .223] | -.208, .435 |
| sex: female-only and urgency: absent | 11 | 1,169 | .102 | [.003, .200] | -.195, .382 |
| **Narrative (narrative vs. non-narrative)** |  |  |  |  |  |
| behavior: prevention and medium: print | 14 | 1,566 | .075 | [.003, .146] | -.145, .288 |
| behavior: prevention and participants: students | 11 | 1,889 | .095 | [.025, .165] | -.122, .304 |
| behavior: prevention and participant sex: both | 14 | 1,789 | .068 | [.003, .132] | -.115, .246 |
| medium: print and participants: students | 10 | 1,344 | .094 | [.016, .171] | -.131, .310 |
| **Metaphorical (vs. non-metaphorical)** |  |  |  |  |  |
| metaphor type: conceptual and policy: domestic | 23 | 10,644 | .125 | [.071, .179] | -.118, .354 |
| metaphor type: conceptual and policy: foreign | 13 | 4,023 | .095 | [.047, .143] | -.054, .240 |
| metaphor type: conceptual and topic: economics | 14 | 7,539 | .110 | [.046, .173] | -.128, .336 |
| metaphor type: conceptual and topic: health and environment | 15 | 4,864 | .077 | [.022, .132] | -.113, .262 |
| metaphor type: conceptual and topic: security and justice | 12 | 4,759 | .075 | [.025, .124] | -.076, .223 |
| metaphor type: linguistic and policy: domestic | 40 | 14,120 | .021 | [.004, .037] | .004, .038 |
| policy: domestic and topic: economics | 22 | 12,944 | .060 | [.019, .101] | -.119, .236 |
| policy: domestic and topic: health and environment | 21 | 6,985 | .055 | [.013, .098] | -.113, .220 |
| **Cultural tailoring (deep-tailored vs. not-tailored)** |  |  |  |  |  |
| region: Asia-Pacific and value: individualism-collectivism | 17 | 1,509 | .123 | [.011, .233] | -.328, .528 |
| region: North American and value: individualism-collectivism | 19 | 1,860 | .125 | [.039, .210] | -.217, .440 |
| **Humor (humorous vs. non-humorous)** |  |  |  |  |  |
| channel: audiovisual and delay: no delay | 20 | 3,675 | .087 | [.004, .168] | -.269, .422 |
| channel: visual and delay: no delay | 15 | 3,041 | .187 | [.056, .311] | -.345, .628 |
| channel: visual and sample: students | 15 | 3,041 | .187 | [.056, .311] | -.345, .628 |
| delay: no delay and involvement: low | 32 | 5,651 | .145 | [.067, .221] | -.282, .524 |
| delay: no delay and sample: students | 45 | 7,498 | .113 | [.041, .184] | -.339, .522 |
| delay: no delay and source sex: male | 18 | 2,887 | .182 | [.051, .307] | -.389, .652 |
| delay: no delay and style: parody | 12 | 1,848 | .221 | [.100, .336] | -.233, .596 |
| delay: no delay and topic: marketing | 19 | 3,639 | .241 | [.161, .318] | -.112, .540 |
| involvement: low and sample: students | 28 | 4,989 | .126 | [.048, .203] | -.270, .486 |
| involvement: low and source sex: male | 16 | 2,681 | .167 | [.036, .291] | -.370, .620 |
| involvement: low and topic: marketing | 12 | 2,351 | .242 | [.120, .356] | -.232, .623 |
| sample: students and source sex: male | 18 | 2,158 | .202 | [.074, .323] | -.350, .650 |
| sample: students and style: parody | 10 | 1,477 | .239 | [.091, .377] | -.298, .661 |
| sample students and topic: marketing | 17 | 3,277 | .243 | [.156, .326] | -.127, .554 |
| **Vividness (vivid vs. pallid)** |  |  |  |  |  |
| congruency: congruent and topic: commercial | 11 | 1,259 | .147 | [.025, .265] | -.279, .525 |
| manipulation: concrete and topic: commercial | 10 | 1,707 | .189 | [.104, .272] | -.081, .433 |
| topic: commercial and valence: positive | 11 | 1,836 | .276 | [.182, .364] | -.041, .542 |
| topic: health and valence: negative | 13 | 1,369 | .109 | [.029, .187] | -.128, .334 |
| **“But you are free” (included vs. omitted)** |  |  |  |  |  |
| requester: present and beneficiary: prosocial | 14 | 4,317 | .196 | [.153, .239] | .089, .299 |
| requester: present and beneficiary: selfish | 18 | 9,117 | .204 | [.153, .255] | .020, .374 |

^a^Note: *k*: number of effect sizes; CI: confidence interval; PI: prediction interval.

**APPENDIX 8**

MOST CONSISTENT MODERATOR EFFECTS:

MEAN EFFECT SIZES, CONFIDENCE INTERVALS, AND PREDICTION INTERVALS^a^

| Message variation | *k* | *N* | mean *r* | [95% CI] | 95% PI |
| --- | --- | --- | --- | --- | --- |
| **Sidedness (two-sided vs. one-sided)** |  |  |  |  |  |
| one moderator: two-sided order: oppose-support | 13 | 1,525 | -.024 | [-.097, .050] | -.222, .176 |
| two moderators: topic: nonadvertising and two-sided order: support-oppose | 10 | 1,146 | .029 | [-.040, .099] | -.121, .178 |
| **Appeal framing (gain vs. loss)** |  |  |  |  |  |
| one moderator: topic: other health | 10 | 1,430 | -.038 | [-.092, .016] | -.101, .026 |
| two moderators: combined gain kernels and undesirable loss kernels | 15 | 2,458 | -.008 | [-.056, .040] | -.118, .102 |
| **Victim description (identifiable vs. non-identifiable)** |  |  |  |  |  |
| one moderator: group unidentified victim | 26 | 14,952 | .014 | [-.042, .071] | -.189, .215 |
| two moderators: non-U.S. location and group unidentified victim | 16 | 14,215 | -.033 | [-.095, .029] | -.197, .132 |
| **Visual material (text-plus-visual vs. text-only)** |  |  |  |  |  |
| one moderator: photograph | 14 | 2,065 | .078 | [.007, .148] | -.152, .300 |
| **Narrative (narrative vs. non-narrative)** |  |  |  |  |  |
| one moderator: participant sex: both | 22 | 5,713 | .055 | [.011, .099] | -.085, .193 |
| two moderators: participants: nonstudents and participant sex: both | 14 | 4,724 | .045 | [-.004, .094] | -.086, .175 |
| **Metaphorical (vs. non-metaphorical)** |  |  |  |  |  |
| one moderator: topic: science and education | 17 | 3,980 | .029 | [-.003, .060] | -.005, .063 |
| two moderators: metaphor type: linguistic and policy: domestic | 40 | 14,120 | .021 | [.004, .037] | .004, .038 |
| **Cultural tailoring (deep-tailored vs. not-tailored)** |  |  |  |  |  |
| one moderator: Europe region | 20 | 1,829 | .034 | [-.032, .100] | -.195, .259 |
| two moderators: Europe region and masculinity-femininity value | 13 | 950 | .015 | [-.075, .105] | -.250, .278 |
| **Depicted threat severity (high vs. low)** |  |  |  |  |  |
| one moderator: images absent | 26 | 4,307 | .120 | [.058, .180] | -.170, .390 |
| **Humor (humorous vs. non-humorous)** |  |  |  |  |  |
| one moderator: political topic | 17 | 2,574 | .035 | [-.047, .116] | -.272, .336 |
| two moderators: low involvement and political topic | 13 | 2,326 | .002 | [-.074, .079] | -.249, .253 |
| **Vividness (vivid vs. pallid)** |  |  |  |  |  |
| one moderator: health topic | 15 | 1,581 | .116 | [.045, .185] | -.092, .314 |
| two moderators: health topic and negative valence | 13 | 1,369 | .109 | [.029, .187] | -.128, .334 |
| **“That’s not all” (included vs. omitted)** |  |  |  |  |  |
| one moderator: product purchase behavior | 14 | 687 | .230 | [.133, .323] | -.011, .446 |
| **Depicted threat vulnerability (high vs. low)** |  |  |  |  |  |
| one moderator: images absent | 23 | 3,186 | .217 | [.143, .289] | -.130, .517 |
| **“But you are free” (included vs. omitted)** |  |  |  |  |  |
| one moderator: requester present | 32 | 13,434 | .203 | [.168, .237] | .057, .341 |
| two moderators: requester present and prosocial beneficiary | 14 | 4,317 | .196 | [.153, .239] | .089, .299 |
| **Argument strength (strong vs. weak)** |  |  |  |  |  |
| one moderator: central-route processing | 13 | 842 | .312 | [.209, .409] | -.034, .591 |
| **Disrupt-then-reframe (vs. reframe-only)** |  |  |  |  |  |
| one moderator: nonprofit context | 10 | 731 | .321 | [.253, .386] | .241, .397 |

^a^Note: *k*: number of effect sizes; CI: confidence interval; PI: prediction interval.
